# Snail mediates repression of the Dlk1-Dio3 locus in lung tumor-infiltrating immune cells

**DOI:** 10.18632/oncotarget.25965

**Published:** 2018-08-17

**Authors:** Svenja Groeneveld, Julien Faget, Nadine Zangger, Etienne Meylan

**Affiliations:** ^1^ Swiss Institute for Experimental Cancer Research, School of Life Sciences, Ecole Polytechnique Fédérale de Lausanne, CH-1015 Lausanne, Switzerland; ^2^ Bioinformatics Core Facility, Swiss Institute of Bioinformatics, CH-1015 Lausanne, Switzerland

**Keywords:** Snail, Dlk1-Dio3 locus, lung adenocarcinoma, microenvironment, miRNA

## Abstract

The epithelial-mesenchymal transition-inducing transcription factor Snail contributes to tumor progression in different malignancies. In the present study, we used a transcriptomics approach to elucidate the mechanism of Snail-mediated tumor growth promotion in a *Kras^LSL-G12D/+^;p53^fl/fl^* mouse model of lung adenocarcinoma. We discovered that Snail mediated the downregulation of the imprinted Dlk1-Dio3 locus, a complex genomic region containing protein-coding genes and non-coding RNAs that has been linked to tumor malignancy in lung cancer patients. The Dlk1-Dio3 locus repression mediated by Snail was found to occur specifically in several populations of tumor-infiltrating immune cells. It could be reproduced in primary splenocytes upon *ex vivo* culture with conditioned medium from Snail-expressing cancer cell lines, which suggests that a Snail-induced soluble factor secreted by the cancer cells mediates the Dlk1-Dio3 locus repression in immune cells, particularly in lymphocytes. Our findings furthermore point towards the contribution of Snail to an inflammatory tumor microenvironment, which is in line with our previous report of the Snail-mediated recruitment of pro-tumorigenic neutrophils to the lung tumors. This underlines an important role for Snail in influencing the immune compartment of lung tumors and thus contributing to disease progression.

## INTRODUCTION

Lung cancer is the leading cause of cancer-related deaths worldwide [1] and the majority of non-small cell lung cancer (NSCLC) patients is diagnosed with metastatic disease [2]. The epithelial-to-mesenchymal transition (EMT) is a developmental program that has been implicated in the formation of metastases [3], and accumulating evidence points towards an important role for EMT in lung cancer malignancy as well [4]. The expression of the key EMT transcription factor Snail is correlated with a poor prognosis in NSCLC patients [5–7], while it is unclear whether Snail contributes to disease progression by actually facilitating metastasis formation.

To elucidate the role of Snail in NSCLC, we have previously used overexpression and silencing approaches to modulate the endogenous levels of Snail in the *Kras^LSL-G12D/+^;p53^fl/fl^* (KP) mouse model of lung adenocarcinoma [8]. We have demonstrated that Snail contributes to the malignant progression of the murine KP lung tumors [5]. In line with other reports that Snail can influence the tumor microenvironment and thereby favor disease progression [9–11], we have shown that Snail engages in a vicious cycle with tumor-infiltrating neutrophils, which contributes to the formation of a pro-tumorigenic tumor microenvironment. We furthermore found that, while Snail mediates an increased infiltration of the tumors with neutrophils, neutrophil depletion did not reduce the increased tumor growth rate caused by Snail [5]. This suggests that the growth-promoting effect of Snail at least partially relies on a mechanism independent of neutrophils.

The imprinted DLK1-DIO3 locus is located on chromosome 14q32 and 12qF1 in human and mouse, respectively. Accordingly, its genes are expressed in a monoallelic fashion depending on the parent-of-origin. The protein-coding genes *delta-like homolog (DLK1), retrotransposon-like protein 1 (RTL1)* and *thyroxine 5-deiodinase (DIO3)* are paternally expressed. Numerous non-coding RNAs (ncRNAs), such as *antisense RTL1* and the long ncRNAs (lncRNAs) *maternally expressed gene 3 (MEG3) and MEG8*, the latter two also termed *gene trap locus 2 (Gtl2)* and *RNA imprinted and accumulated in nucleus (Rian)* in mice, are exclusively expressed from the maternal allele. Besides numerous other ncRNAs, including small nucleolar (sno) RNAs, PIWI-interacting (pi) RNAs and lncRNAs, the DLK1-DIO3 locus contains 54 micro RNAs (miRNAs; 53 in mice) the largest known miRNA cluster in the human and mouse genome, respectively. The allele-specific expression is mainly orchestrated by imprinting control regions (ICRs). The primary, DLK1-MEG3 intergenic differentially methylated region (Ig-DMR) is germline-derived, while the secondary MEG3-DMR is established post-fertilization and resides in the MEG3 promoter. Both DMRs are hypermethylated on the paternal and hypomethylated on the maternal allele [12]. Correct imprinting and allele-specific expression of the DLK1-DIO3 locus members is crucial during embryo development [13, 14]. Furthermore, the DLK1-DIO3 locus members have been implicated in diverse human diseases, including cancer, schizophrenia and diabetes [12]. Strikingly, recent reports have linked the DLK1-DIO3 locus to lung cancer [15].

In the present study, we aimed to elucidate how Snail contributes to lung tumor progression in a murine model of lung adenocarcinoma. We discovered that the Dlk1-Dio3 locus is repressed by Snail in KP lung tumors. Intriguingly, Snail mediates the Dlk1-Dio3 locus repression specifically in tumor-infiltrating immune cells in a paracrine fashion *via* the secretion of a soluble factor by epithelial tumor cells.

## RESULTS

### Snail mediates repression of the imprinted Dlk1-Dio3 locus in KP lung tumors

**Figure 1:**
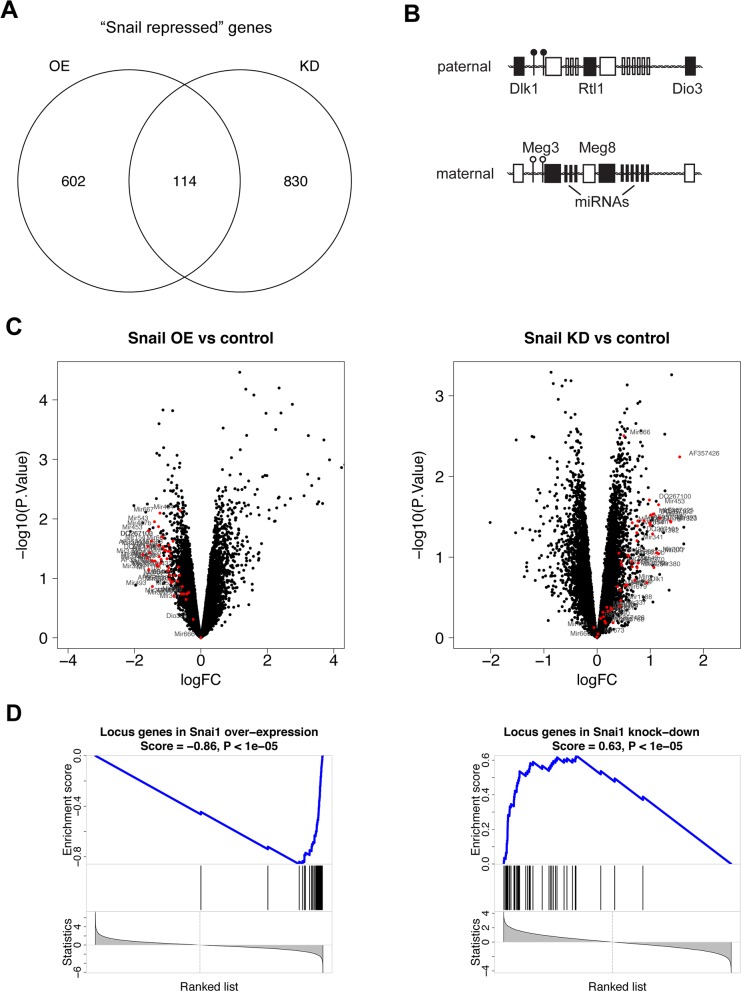
Snail represses the Dlk1-Dio3 locus in Kras-driven lung adenocarcinomas **(A)** Venn diagrams representing the intersection between differentially expressed genes (p-value < 0.1), downregulated (log fold change < 0) in individual Snail overexpressing (n = 4) compared to control (n = 4) tumors and upregulated (log fold change > 0) in individual Snail knockdown (n = 5) relative to control (n = 6) tumors, containing “Snail repressed” genes, based on microarray analysis. **(B)** The imprinted DLK1-DIO3 locus on human chromosome 14q32 and mouse chromosome 12qF1 contains the largest miRNA cluster of the genome and numerous further ncRNAs, which are all expressed from the maternal allele. The protein-coding genes *DLK1, RTL1* and *DIO3* are paternally expressed. Two differentially methylated regions (DMRs) control the imprinting and are hypermethylated on the paternal and hypomethylated on the maternal allele. *Black boxes*: expressed; *white boxes*: not expressed; *black circle*: hypermethylated DMR; *white circle*: hypomethylated DMR. **(C)** Volcano plots depicting the expression of all genes in (*left*) Snail OE and (*right*) Snail KD tumors relative to the respective controls. Each dot represents one gene in function of fold change and p-value. Genes located within the Dlk1-Dio3 locus are highlighted in red. **(D)** Enrichment analysis of a gene set comprising all Dlk1-Dio3 locus genes in (*left*) Snail OE and (*right*) Snail KD tumors. *OE*: overexpression, *KD*: knockdown.

In the KP mouse model of lung adenocarcinoma, we aimed to elucidate the mechanism of Snail-mediated tumor progression. We therefore performed Snail overexpression (OE) or knockdown of endogenous Snail (KD) in the lung tumors via a doxycycline-inducible system or constitutive shRNA expression, respectively (Supplementary Figure 1A). To use an unbiased transcriptomics approach, we performed microarray analyses of individually dissected KP tumors with confirmed Snail overexpression or knockdown (Supplementary Figure 1B). As Snail exerts well-characterized transcriptional repressor functions [16], we focused on the differentially expressed genes that were downregulated in Snail OE tumors (602 + 114 genes; Supplementary Table 1) and upregulated in Snail KD tumors (830 + 114 genes; Supplementary Table 2), relative to the respective control samples. We termed the 114 genes within this intersection the “Snail repressed” genes (Figure 1A and Supplementary Table 3). Interestingly, the intersection included numerous genes located within the imprinted Dlk1-Dio3 locus (Figure 1B), which accounted for 23% of the genes. Among them were many genes coding for miRNAs, such as *Mir377, Mir485, Mir453, Mir323* and *Mir300*, the lncRNA *Meg8* (*Rian)* and *Meg3* (Supplementary Figure 1C). We therefore considered the possibility that Snail affects the activity of the Dlk1-Dio3 locus as a whole rather than impacting the transcription of individual genes located within this region. Further analysis of all genes in the Dlk1-Dio3 locus captured by the microarray confirmed that indeed all of them displayed decreased expression upon Snail induction and were upregulated upon Snail knockdown (Figure 1C). Although the expression change of each gene considered individually was rather modest and did not reach statistical significance in most instances, we found a highly significant enrichment among the downregulated and upregulated genes in Snail OE and KD tumors, respectively, by encompassing all genes of the Dlk1-Dio3 locus in a gene set (Figure 1D). This confirms that Snail mediates the repression of the imprinted Dlk1-Dio3 locus in murine lung adenocarcinomas.

Beyond the Dlk1-Dio3 locus, the intersection of “Snail repressed” genes contained genes coding for the tight junction proteins claudin (CLDN) 2 and 3. The related CLDN1 [17] CLDN3, 4 and 7 [18] are transcriptionally repressed by Snail and the expression of CLDN1 and 5 in human lung tumors is inversely correlated with Snail [19]. Consistently, we found that the expression of both CLDN2 and 3 were downregulated in human and murine lung cancer cells expressing Snail (Supplementary Figure 1D, 1E).

Overrepresentation analysis (ORA) of the “Snail repressed” intersection yielded the top pathways G2M CHECKPOINT and FATTY ACID METABOLISM (Supplementary Table 4).

### Snail mediates Dlk1-Dio3 locus repression in tumor-infiltrating immune cells

**Figure 2:**
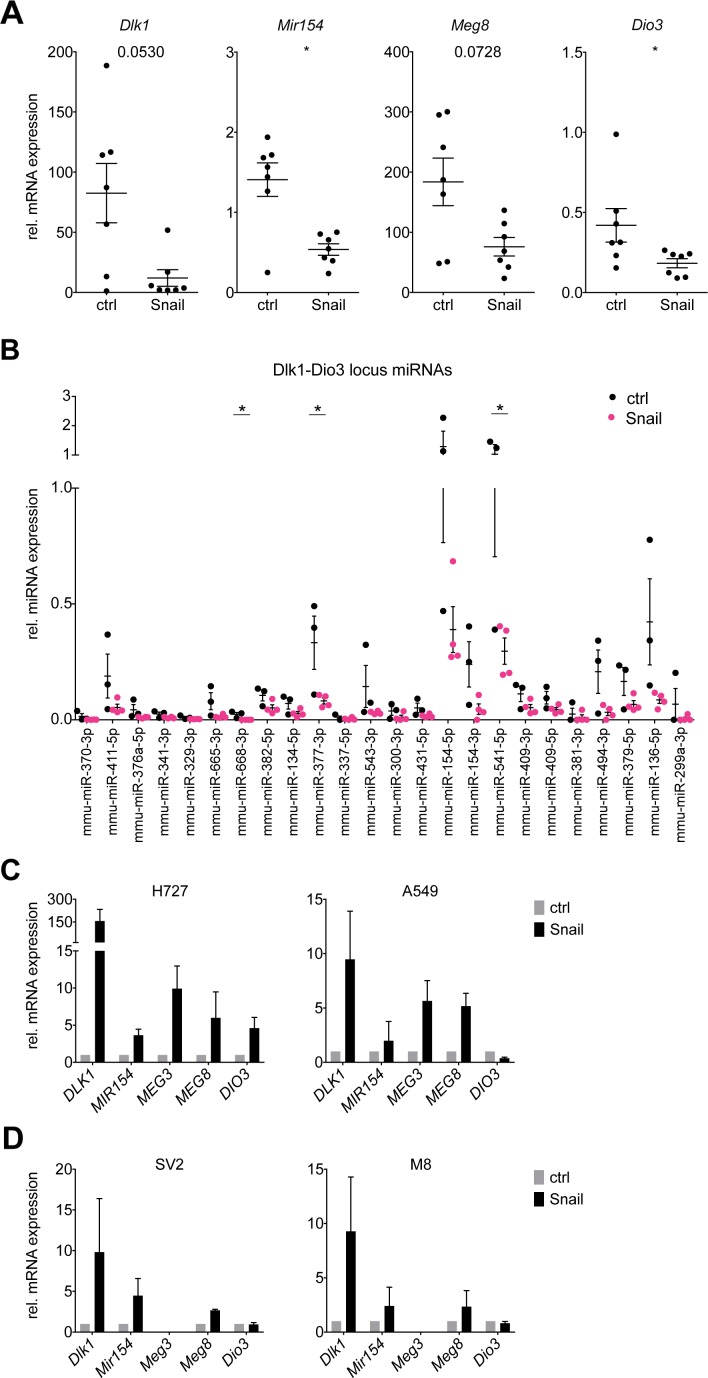
Snail represses Dlk1-Dio3 locus genes and mature miRNAs in KP lung tumors **(A)** Real time PCR analysis of mRNA expression of *Dlk1, Mir154, Meg8* and *Dio3* in individual whole control KP (n = 7) and Snail overexpressing KPR (n = 7) tumors. **(B)** Real time PCR analysis of the expression of mature miRNAs encoded by the Dlk1-Dio3 locus in individual whole control KP (n = 3) and Snail overexpressing KPR (n = 4) tumors. **(C, D)** Real time PCR analysis of mRNA expression of (C) *DLK1, MIR154, MEG3, MEG8* and *DIO3* in the human NSCLC cell lines H727 and A549 (n = 3) and (D) *Dlk1, Mir154, Meg3, Meg8* and *Dio3* in the murine KP cell lines SV2 and M8 (n = 3). The cells were stably transduced with a Tet-On system and treated or untreated during 6 days with doxycycline. Gene expression is normalized to the non-induced condition. Graphs shows mean with SEM. Statistical analysis based on (A) Mann-Whitney test, (B) Multiple *t*-tests: ^*^: p < 0.05; not indicated: not significant.

To investigate the Dlk1-Dio3 locus repression by Snail in detail, we selected several locus members whose expression was assessed as a surrogate for the total locus activity. Real-time PCR analysis of the mRNA expression for the protein-coding genes *Dlk1* and *Dio3*, the miRNA-coding *Mir154* and the lncRNA *Meg8* confirmed the microarray results and their downregulation in Snail overexpressing tumors (Figure 2A). In addition to the reduced transcription of the Dlk1-Dio3 locus members, the expression levels of 37 locus miRNAs were analyzed to determine whether the generation of mature miRNAs was likewise suppressed by Snail. Twenty-two miRNAs could be detected in the tumors and for most of them, a trend towards lower expression was observed in Snail-induced tumors, which reached statistical significance only in 3 instances due to a high variability in miRNA levels (Figure 2B).

We next sought to investigate the mechanism of Snail-mediated Dlk1-Dio3 locus repression *in vitro*. To that end, the expression of several locus members was assessed in Snail-inducible human NSCLC (H727 and A549) cell lines. Surprisingly and in contrast to our *in vivo* findings, the mRNA expression of *DLK1, MIR154, MEG3, MEG8* and *DIO3* was consistently upregulated in the human cell lines upon Snail overexpression (Figure 2C). To ensure that this discrepancy between *in vivo* and *in vitro* results did not originate in interspecies differences in Dlk1-Dio3 locus regulation, we generated cell lines from lung tumors of the KP model and likewise transduced them to enable doxycycline-induced Snail expression. However, as for the human cell lines, the Dlk1-Dio3 locus genes were consistently upregulated upon Snail induction in the mouse lung tumor cell lines (Figure 2D). Thus, a cell-intrinsic mechanism as explanation for the Snail-mediated Dlk1-Dio3 locus downregulation observed *in vivo* does not seem likely.

We have previously reported that Snail overexpression in lung tumors alters the composition of tumor-infiltrating immune cells, namely an increase in infiltrating neutrophils and a decrease in B cells [5]. Analysis of the “Snail activated” genes, i.e. those upregulated in Snail OE tumors and downregulated in Snail KD tumors, revealed many immune response genes, which accounted for 49% of the genes in the intersection (Supplementary Figure 2A). Among those genes, many are involved in inflammation and particularly participate in interferon and tumor necrosis factor (TNF) signaling and the list of “Snail activated” genes suggests that Snail stimulates an inflammatory response involving both the adaptive and innate immune system (Supplementary Figure 2B). Indeed, ORA of the intersection of “Snail activated” genes confirmed that the majority of the significantly enriched pathways (FDR < 0.5) of the Hallmark collection are pathways of immunological and particularly of inflammatory processes (Table 1). However, as the Snail overexpressing tumors grow faster [5], this implies that instead of mounting an effective tumor control, the changes in the immune microenvironment rather give rise to chronic inflammation, ultimately favoring tumor progression.

**Table 1:** Overrepresented pathways from the Hallmark collection among the genes of the intersection of “Snail activated” genes (n = 113)

Gene Set	p-value	FDR
INTERFERON GAMMA RESPONSE	5.16E-18	2.58E-16
TNFA SIGNALING VIA NFKB	4.55E-13	5.68E-12
INFLAMMATORY RESPONSE	4.55E-13	5.68E-12
ALLOGRAFT REJECTION	4.55E-13	5.68E-12
IL2-STAT5 SIGNALING	1.12E-06	1.12E-05
IL6-JAK-STAT3 SIGNALING	2.13E-06	1.78E-05
KRAS SIGNALING UP	9.27E-06	6.62E-05
INTERFERON ALPHA RESPONSE	5.28E-05	3.30E-04
EPITHELIAL MESENCHYMAL TRANSITION	2.50E-03	1.39E-02
HYPOXIA	1.20E-02	5.00E-02
COMPLEMENT	1.20E-02	5.00E-02
P53 PATHWAY	1.20E-02	5.00E-02

*FDR:* false discovery rate

“Snail activated” genes are involved in inflammatory processes. Overrepresentation analysis using Fisher's exact test was computed of pathways as defined by the Hallmark biological process collection of MSigDB among the genes in the “Snail activated” intersection (n = 113) from Supplementary Figure 2A (cutoff: FDR > 0.05).

**Figure 3:**
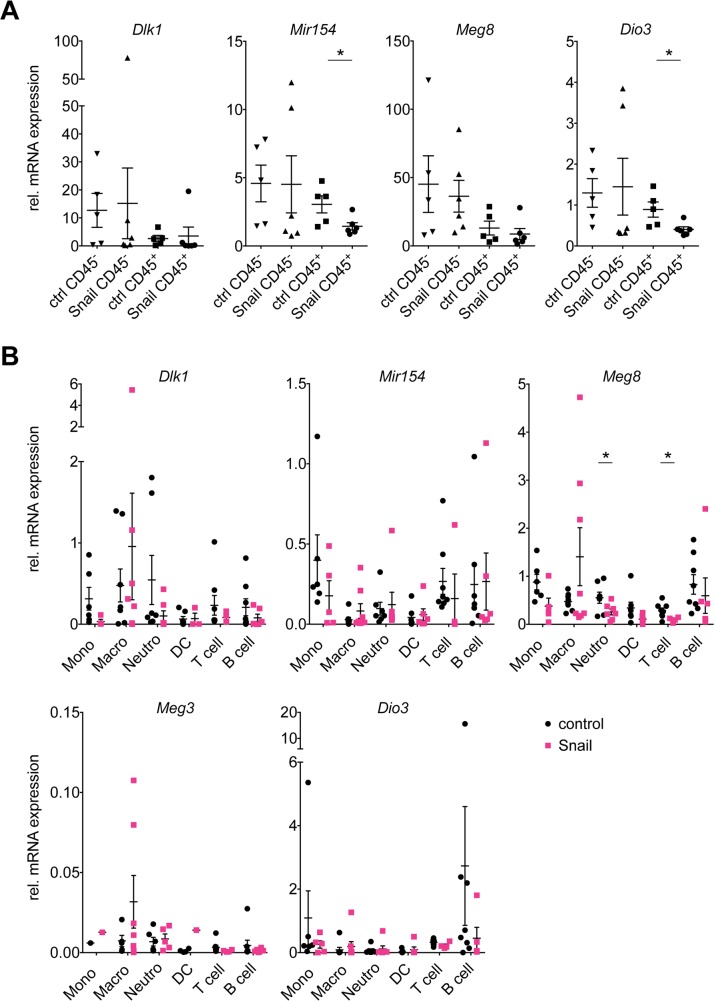
Snail represses the Dlk1-Dio3 locus in lung tumor infiltrating immune cells Real time PCR analysis of mRNA expression of *Dlk1, Mir154, Meg8* and *Dio3* in **(A)** CD45^−^ tumor (ctrl: n = 5, Snail: n = 6) and CD45^+^ immune (ctrl: n = 5, Snail: n = 6) cell fractions isolated from individual control KP and Snail overexpressing tumors using magnetic beads and **(B)** in addition *Meg3* in six immune populations, i.e. monocytes (Mono), macrophages (Macro), neutrophils (Neutro), dendritic cells (DC), T and B lymphocytes (T and B cell, respectively), that were FACS-sorted from individual control KP (n = 11) and Snail overexpressing KPR (n = 8). (A, B) Graphs show mean with SEM. Statistical analysis based on (A) Mann-Whitney test and (B) Multiple *t*-tests: ^*^: p < 0.05; not indicated: not significant.

As the microarray analysis had been performed on whole tumor samples, we wanted to dissect the contribution of the tumor epithelial cells and the tumor immune compartment to the gene expression results. We therefore sorted individual control or Snail overexpressing KP lung tumors into CD45^−^ and CD45^+^ cell fractions using magnetic bead isolation with the CD45^+^ fraction comprising the tumor-infiltrating immune cells and the CD45^−^ fraction containing all other cells, including tumor epithelial cells, stromal cells and endothelial cells. The *Snai1* overexpression could be clearly detected in the respective CD45^−^ fraction. While *Snai1* mRNA levels in the CD45^+^immune cells were generally lower, the *Snai1* expression of the immune fraction derived from the OE tumors was increased (possibly due to some contamination with CD45^−^ non-immune cells) (Supplementary Figure 3A). Interestingly, the Dlk1-Dio3 locus downregulation could be observed only among the sorted CD45^+^ cells (Figure 3A). This discrepancy of the findings in the CD45^−^ cells compared to those of the cultured cell lines (Figure 2C, 2D) might be explained by the contribution of cell types other than carcinoma cells to the CD45^−^ fractions. Furthermore, previous observations point towards difficulties of studying the Dlk1-Dio3 locus gene expression *in vitro*, due to a global, but reversible downregulation under cell culture conditions [20].

According to previous immune profiling of the KP lung tumors [5], the contribution of immune cells to the total cellular content of the tumors averages around 15-20%. A Snail-mediated repression of the Dlk1-Dio3 locus predominantly in tumor-infiltrating immune cells would hence affect only a minor proportion of the cells comprising the total tumor mass, although it would probably still be detectable by microarray analysis of whole tumor mRNA. This is in line with the relatively modest, although consistent, locus downregulation observed in whole tumor lysates of Snail OE tumors and upregulation upon Snail KD (Figure 1C).

Our results thus indicate that the Snail mediated Dlk1-Dio3 locus repression rather occurs in the tumor-infiltrating immune cells than in the tumor epithelial cells. This might be explained by two possible scenarios: First, Snail might favor the preferential infiltration of the tumors with immune cells lowly expressing the Dlk1-Dio3 locus, leading to a *de facto* lower locus expression in the immune compartment. Second, Snail might repress the Dlk1-Dio3 locus expression broadly across several immune cell types. To evaluate these possibilities, we FACS-sorted six immune cell populations from control or Snail overexpressing KP lung tumors, namely monocytes, macrophages, neutrophils, dendritic cells (DCs), T and B lymphocytes. Differences of the *Snai1* mRNA levels were observed between the different populations with the highest expression in neutrophils, followed by monocytes, macrophages and DCs, and the lowest in T and B lymphocytes. Interestingly, neutrophils and macrophages sorted from Snail OE tumors exhibited higher *Snai1* mRNA levels compared to those sorted from the non-induced tumors (Supplementary Figure 3B). Analysis of mRNA levels revealed that the expression of the Dlk1-Dio3 locus genes was within the same range among the different immune cell types (Figure 3B). However, the expression of *Mir154*, *Meg8* and *Dio3* was higher in B cells compared to neutrophils, while we have previously reported a decreased B cell and increased neutrophil infiltration in Snail OE tumors [5]. Furthermore, the expression of the locus genes was affected by Snail in almost all of the immune cell types examined: in monocytes, *Dlk1, Mir154* and *Meg8* expression was decreased, whereas in neutrophils, only *Dlk1* and *Meg8* were reduced and in DCs, only *Meg8* was affected. In T and B lymphocytes, *Dlk1, Meg8, Meg3* and *Dio3* were decreased, in T lymphocytes additionally *Mir154*. Macrophages on the other hand did not show reduction in any of the genes. Due to high variation of the expression values between the individual samples, statistical significance was only reached for *Meg8* in neutrophils and B cells, whereas otherwise only a trend was observed. The Snail-mediated Dlk1-Dio3 locus repression therefore appears to be a general mechanism affecting multiple immune cell types. In conclusion, both a differential infiltration of immune cells with different levels of Dlk1-Dio3 locus expression and actual Dlk1-Dio3 locus downregulation across different immune cells might contribute to the observed Dlk1-Dio3 locus repression in whole Snail OE tumors (Figure 2A) and in CD45^+^ immune cell fractions (Figure 3A). While we observed a global impact on the Dlk1-Dio3 locus expression, the analysis of the different immune populations revealed that distinct subsets of genes were downregulated, suggesting a cell-type specific fine-tuning of the Dlk1-Dio3 locus expression in immune cells. Other immune populations, beyond the herein FACS-sorted, might likewise have contributed to the phenotype.

### A secreted soluble factor mediates the repression of the Dlk1-Dio3 locus in immune cells by Snail

As the Snail levels were modulated in the tumor epithelial cells and the Dlk1-Dio3 locus expression changes occurred in the tumor immune compartment, we aimed to understand how the Snail-mediated Dlk1-Dio3 locus repression in immune cells might be regulated in terms of cellular communication. It has been reported previously that Snail-expressing cancer cells can influence immune cells *via* the secretion of mediator molecules. Snail-expressing melanoma cells can cause impaired DC function *via* the secretion of thrombospondin 1 [11]. Furthermore, Snail can favor the recruitment of tumor-associated macrophages *via* CCL2 and 5 production in several cancer cell types [9] and mast cells *via* SCF-1 release by pancreatic ductal adenocarcinoma cells [10]. We therefore hypothesized that a soluble factor secreted by Snail-expressing lung cancer cells likewise mediates the Dlk1-Dio3 locus repression in immune cells.

**Figure 4:**
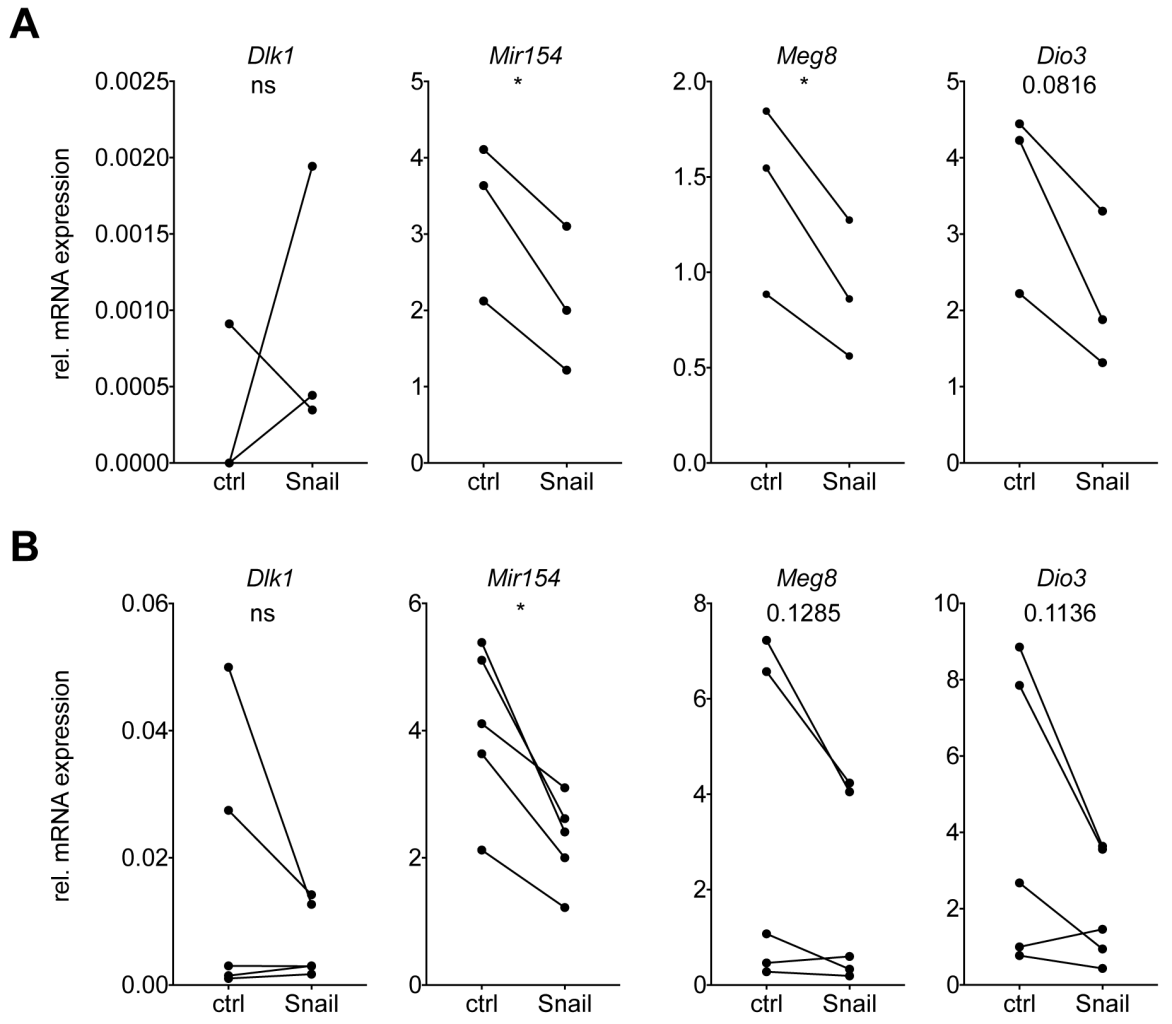
Snail mediates Dlk1-Dio3 locus repression in immune cells *via* the secretion of a soluble factor Real time PCR analysis of mRNA expression of *Dlk1, Mir154, Meg8* and *Dio3*, relative to *Rpl30*, in primary splenocytes isolated from healthy mice and incubated during 48 h with CM from control or Snail overexpressing **(A)** murine M8 (n = 3) or **(B)** human H2122 (n = 5) cells. Statistical analysis based on paired *t*-test, ^*^: p < 0.05; ns: not significant.

We therefore collected conditioned medium (CM) from Snail-inducible human or murine lung cancer cell lines and cultured primary murine splenocytes isolated from healthy mice *ex vivo* during 48 hrs. Intriguingly, the expression of several Dlk1-Dio3 locus genes was downregulated in the splenocytes following culture with CM from Snail-expressing murine or human lung cancer cells (Figure 4A and 4B). This result is in accordance with the possible secretion of a soluble factor by Snail-expressing cancer cells mediating the Dlk1-Dio3 locus downregulation in immune cells. As the spleen is predominantly composed of T and B lymphocytes, this is consistent with the finding that those populations, upon being sorted from Snail overexpressing tumors, exhibited the most consistent downregulation of Dlk1-Dio3 locus genes (Figure 3B).

**Figure 5:**
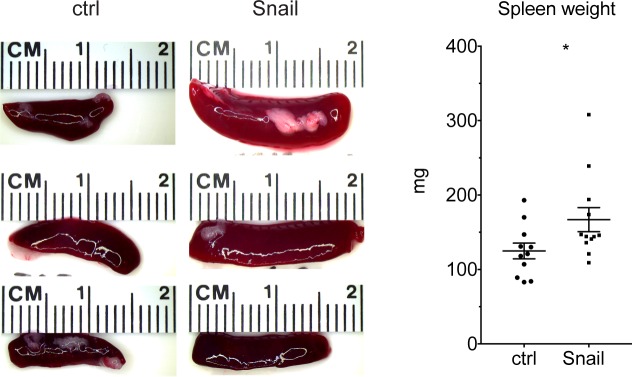
Snail expression in lung tumors leads to splenomegaly Representative photographs of spleens isolated from control (n = 11) or Snail overexpressing (n = 12) mice. Graph shows quantification of spleen weight at the endpoint. Graph shows mean with SEM. Statistical analysis based on Mann-Whitney test: ^*^: p < 0.05.

We have reported previously that Snail can enhance the infiltration of the KP lung tumors with neutrophils, possibly by activating a feed-forward loop of Cxcl2-mediated neutrophil recruitment. We show herein that Snail-expressing cancer cells can repress the Dlk1-Dio3 locus activity across several immune populations. These findings, together with the above-mentioned reports from other groups, point towards a paracrine effect of Snail-expressing cancer cells on immune cells. Therefore, we wanted to monitor if Snail overexpression has a remote effect on secondary lymphoid organs in tumor-bearing mice. Indeed, we discovered that the tumor-bearing, Snail overexpressing mice displayed splenomegaly (Figure 5), which corroborates a potentially long-range communication of Snail expression in lung tumor epithelial cells on immune cells and secondary lymphoid organs.

Exosomes are involved in the regulation of numerous biological processes and recently have gained attention in the cancer field, where they have been implicated in metastasis, angiogenesis and immune regulation [21]. To determine if the release of exosomes is involved in the Snail-mediated Dlk1-Dio3 locus repression, we isolated the exosome fraction from CM following a modified protocol adapted from Kowal *et al* [22], while preserving the exosome-free CM for comparison. Treatment of primary murine splenocytes *ex vivo* with either exosome-free CM or exosome preparations, diluted in fresh medium to the appropriate concentrations, indicated that exosomes are probably not required for the Dlk1-Dio3 locus repression. The downregulation of Dlk1-Dio3 locus genes occurred exclusively upon culture with exosome-free CM (Supplementary Figure 4A) from Snail-overexpressing cells, while it was not observed after treatment with the corresponding exosome preparation (Supplementary Figure 4B). In conclusion, the Dlk1-Dio3 repression in Snail-expressing lung tumors seems to be mediated by a soluble factor secreted from Snail-expressing cancer cells, which does not reside in exosomes.

As we had observed gene expression changes in Snail overexpressing tumors indicative of an inflammatory tumor microenvironment (Supplementary Figure 2), we considered the possibility that the Snail-mediated induction of inflammatory cytokines could cause the Dlk1-Dio3 locus repression in immune cells. Both *Il6* and *Tnf* expression was increased in Snail overexpressing tumors (Supplementary Figure 5A, 5B), whereas no increase in *Ifnb1* or *Ifng* was observed, raising the possibility that the interferon-inducible gene signature might have been activated *via* another pathway in response to Snail overexpression. In the human lung cancer cells (Supplementary Figure 5C), from which the CM was derived for the *ex vivo* culture (Figure 4B and Supplementary Figure 4A), *TNF, IL6* and *IFNB1* were upregulated upon Snail OE, while *IFNG* was undetectable. In the corresponding murine KP lung adenocarcinoma cell lines, the expression of *Il6* and *Ifng* was increased. This suggests that while certain species-specific differences exist as to which cytokine is upregulated, both human and murine lung cancer cells induce inflammatory mediator production in response to Snail. Treatment of isolated primary murine splenocytes with two different concentrations of IL6 or TNF did however not decrease the expression of Dlk1-Dio3 locus genes but, contrarily, increased it (Supplementary Figure 5D). Potentially elevated secretion of IL6 and TNF by Snail-expressing cancer cells can therefore not explain the observed Dlk1-Dio3 locus repression in tumor-infiltrating immune cells.

In conclusion, these results point towards a non-cell autonomous regulation of the Dlk1-Dio3 locus in immune cells by a soluble factor secreted by Snail expressing cancer cells that does not reside in exosomes. Further studies aimed at identifying this secreted factor will be very informative to elucidate the nature of the molecular pathways involved in the paracrine Snail-mediated Dlk1-Dio3 locus regulation and their functional consequences in immune cells.

## DISCUSSION

In this study, we provide evidence that the EMT-inducing transcription factor Snail represses Dlk1-Dio3 locus gene expression in KP lung tumors. An increasing body of literature links the DLK1-DIO3 locus to lung cancer and has been recently reviewed by Enterina *et al* [15]. DLK1-DIO3 locus members are involved in lung development. In murine models, Dlk1 stimulates pulmonary branching and alveolar morphogenesis at the fetal stage through Notch signaling [23]. In both human and mouse, several DLK1-DIO3 locus miRNAs, including miR-134, miR-154, miR-299, miR-323, miR-337, miR-368 and miR-370, are highly expressed in the developing lung and downregulated in the adult organ [24]. Mice with maternal deletion of *Meg3* die within a month postnatally due to impaired development of pulmonary alveoli [13].

In the K mouse model of lung adenocarcinoma, the Dlk1-Dio3 locus, including the miRNA cluster and *Rtl1, Meg3* and *Dlk1*, was found upregulated in lung tumors compared to normal lung tissue [20]. Likewise, in human lung adenocarcinoma, the DLK1-DIO3 locus is reportedly upregulated compared to healthy lung tissue [20, 25]. Nadal *et al* reported that high DLK1-DIO3 locus miRNA expression was furthermore correlated with a significantly reduced overall survival in patients. Particularly the expression of miR-370, miR-441 and miR-376a predicted a poor clinical outcome [25]. Another class of small noncoding RNAs, piRNAs, is also encoded by the DLK1-DIO3 locus. Incorporating piRNA expression into the prognostic miRNA signature described by Nadal *et al* improved the predictive performance further [26]. In NSCLC patients, a smoking-related change in methylation pattern of the DLK1-DIO3 locus was described, which was not associated with chronic obstructive pulmonary disease (COPD), but occurred across both lung adenocarcinoma and squamous cell carcinoma [27]. In contrast to the miRNA cluster, *MEG3* expression predicts a favorable outcome in NSCLC patients [28]. *MEG3* acts as a tumor suppressor *via* multiple mechanisms and its expression is decreased across several cancer types, possibly by promoter or Ig-DMR hypermethylation [29]. Additionally, miR-134 also displays tumor suppressor functions and inhibits proliferation in human NSCLC cells [30].

The above-mentioned studies suggest an involvement of the Dlk1-Dio3 locus in the pathology of lung cancer. This is intriguing, as in KP lung tumors, Snail inhibits Dlk1-Dio3 locus gene expression while we have previously demonstrated that it enhances malignant progression in the KP mouse model of lung adenocarcinoma [5].

Our results suggest that the downregulation of the Dlk1-Dio3 locus genes occurs in the tumor-infiltrating immune cells rather than in the carcinoma cells expressing Snail. This is a novel perspective, as the available reports on DLK1-DIO3 locus deregulation in human NSCLC are based on expression or methylation data derived from bulk tumors [20, 25–27]. In contrast, in murine lung adenocarcinomas, Valdmanis *et al* demonstrated that the Dlk1-Dio3 locus upregulation was specific to Tomato-labelled sorted *Kras^G12D^* mutant cancer cells [20]. However, the general Dlk1-Dio3 locus upregulation during lung tumorigenesis does not contradict a Snail-mediated locus repression in tumor-infiltrating immune cells, but points towards two possibly independent regulatory mechanisms. Herein, we have demonstrated that Snail-expressing cancer cells release a soluble factor that does not reside in exosomes and is capable of downregulating the Dlk1-Dio3 locus in immune cells. Future investigation aimed at identifying the unknown factor should be conducted.

How the expression of the DLK1-DIO3 locus is controlled is not yet completely understood and appears to involve an intertwined regulatory mechanism. A 210 kb polycistron, spanning from *Meg3* to *Mirg* and encompassing numerous miRNA genes, is transcribed from a single promoter in the *Meg3* gene *en bloc,* which is regulated by binding of the AF4/FMR family protein Aff3 to the *Meg3* proximal enhancer of the unmethylated maternal allele [31]. This provides an explanation as to why the expression of numerous DLK1-DIO3 locus members is commonly affected simultaneously. Accordingly, genetic deletion of the maternal, but not the paternal, lncRNA *Meg3* abolished the expression of the downstream maternal genes and furthermore caused methylation of the maternal Ig-DMR while it activated the expression of previously silenced paternal genes [14]. Polycomb repressive complex 2 (PRC2) reportedly inhibits the methylation of the maternal Ig-DMR enhancer *via* the binding of enhancer of zeste homolog 2 (EZH2) to *Meg3* and sequestering the DNA (cytosine-5)-methyltransferase 3B (DMNT3) [32]. On the other hand, several Dlk1-Dio3 locus miRNAs have been proposed to target PRC2 components and thus to enhance the locus expression *via* a positive feedback loop [33]. The miR-127 / miR-136 cluster on the locus has been proposed to regulate the expression of *Rtl1* [12] and six miRNAs supposedly target *Dlk1* [34].

As the imprinting of the DLK1-DIO3 locus is governed by allele-specific differential methylation of the DMRs, alterations in their methylation pattern might contribute to the observed changes in gene expression upon Snail overexpression. Loss of imprinting (LOI) is frequently observed in cancer and represents an early event in tumorigenesis [35]. In the case of LOI, we would expect to observe a downregulation of maternally, with simultaneous increase in paternally, expressed genes or vice versa. However, upon Snail overexpression, both paternal genes and maternal ncRNAs were concomitantly downregulated, arguing against the involvement of LOI in favor of a rather general repressive mechanism. In support of this, Valdmanis *et al* found no change in DMR methylation in murine lung tumors compared to normal lung tissue, suggesting that another mechanism of transcriptional regulation is involved [20].

Repression of the miR-379 / miR-656 cluster in glioblastoma multiforme was accompanied by locus hypermethylation [36]. In ductal breast carcinoma, hypermethylation of CpG islands upstream of the DLK1-DIO3 locus has been proposed to repress its expression [37]. Upregulation of DLK1 in NSCLC has furthermore been reported upon hypomethylation [27, 38]. Knockout mice lacking expression of the lncRNA *Hotair*, which directs H3K27 methylation and H3K4 demethylation for the silencing of target genes by binding to PRC2 and lysine-specific histone demethylase 1A (LSD1) complexes, display a de-repression of both alleles of the Dlk1-Dio3 locus and other imprinted loci [39]. Intriguingly, Snail has been found to interact with EZH2 *via Hotair* and thereby regulate gene expression in hepatocytes [40]. Thus, had we observed the Snail-mediated Dlk1-Dio3 locus repression cell-intrinsically, it could have potentially been attributed to a Snail and *Hotair* mediated targeting of chromatin repressive complexes such as PRC2 to the locus. However, as the herein described Dlk1-Dio3 locus repression caused by Snail occurred *via* a paracrine mechanism, it can probably not be attributed to a Snail-mediated recruitment of repressive chromatin modifiers. In any case, it will be interesting to assess potential alterations in the Dlk1-Dio3 locus methylation beyond merely the DMRs across the affected immune populations in Snail overexpressing tumors by sequencing approaches.

While an increasing body of literature contributes to our understanding of the Dlk1-Dio3 locus in cancer cells, its role in tumor-infiltrating immune cells remains elusive and reports on its functions in immune cells in general are sparse. In B cell malignancies, miR-377 reportedly targets BCL-xL and the locus miRNAs were downregulated upon chemotherapy treatment of chronic lymphocytic leukemia (CLL) patients [41]. In schizophrenia patients, DLK1-DIO3 locus miRNAs were found downregulated in peripheral blood mononuclear cells (PBMCs), which recapitulated changes in miRNA expression in the entorhinal cortex, a region associated with schizophrenia. The functional consequences of this observation however remained elusive [42]. Strikingly, across three genetic mouse models of systemic lupus erythematosus, in which the inflammatory autoimmune reaction is characterized by aberrant autoantibody production, numerous Dlk1-Dio3 locus miRNAs were progressively upregulated in splenic T and B lymphocytes during lupus development [43]. In a subsequent study, this upregulation was attributed to DNA hypomethylation in splenocytes. Interestingly, the silencing of several Dlk1-Dio3 locus miRNAs in primary splenocytes prevented the induction of inflammatory cytokines, including Ifnγ, Il1β, Il6 and furthermore Il10, upon LPS stimulation [44].

While contrasting with our results of Snail leading to Dlk1-Dio3 repression in immune cells and simultaneously inducing an inflammatory microenvironment, these findings clearly illustrate a role of Dlk1-Dio3 locus miRNAs in regulating immune cell function, which might be context dependent. This was observed by Dai *et al* particularly in T and B lymphocytes [44], which were also the populations with the most striking locus downregulation in Snail overexpressing KP tumors. The analysis of the methylation status of the Dlk1-Dio3 locus in sorted immune populations from Snail overexpressing KP lung tumors might provide insight into the mechanism of transcriptional repression. How the Snail-mediated downregulation of the Dlk1-Dio3 locus affects the activity of lymphocytes and other immune populations in a cancer context needs to be evaluated in functional studies.

In conclusion, our results shed light on a connection between the EMT-inducing transcription factor Snail that contributes to malignant progression in lung cancer, with the Dlk1-Dio3 locus, recently discovered to be involved in lung cancer malignancy and correlated with clinical outcome in lung cancer patients. The Snail-mediated Dlk1-Dio3 locus repression in tumor-infiltrating immune cells might contribute to a disease-promoting, chronic inflammatory microenvironment.

## MATERIALS AND METHODS

### Mouse experimentation

*Kras^LSL-G12D/WT^* and *p53^FL/FL^* mice in a C57BL6/J background were bred to obtain *Kras^LSL-G12D/WT^; p53^FL/FL^* (KP) mice. KP mice were bred with *CCSP-rtTA* (R) mice in the same background to generate KPR mice. K, P and R mice were purchased from The Jackson Laboratory. All mouse experiments were performed with the permission of the Veterinary Authority of the Canton de Vaud, Switzerland (license number VD2391). The tumors were initiated upon infection of lung epithelial cells with a viral vector delivering Cre recombinase to activate oncogenic *Kras^G12D^* and delete *p53* [8]. Twelve-to-fourteen week old mice were instilled intratracheally with 2.000 Cre-active lentiviral units as described by DuPage *et al* [45]. We have previously described the generation of the lentiviral constructs allowing for doxycycline-inducible Snail expression and the Snail induction modalities in the KP lung tumors [5]. Briefly, the lenti-Pgk:Cre_TRE:Snai1-FLAG *in vivo* construct was generated by amplifying the mouse *Snai1* sequence from cDNA purchased from Thermo Scientific and the polymerase chain reaction (PCR) product was cloned downstream to tetracycline response element (TRE) repeats into a dual-promoter lentiviral vector that expresses Cre recombinase from the Pgk promoter. The shRNAs against *Snai1* and *p53* as *in vivo* control shRNA were designed using the pSICOLIGOMAKER 1.5 program (http://web.mit.edu/jacks-lab/protocols/pSico.html; created by A. Ventura, Memorial Sloan-Kettering Cancer Center). The oligonucleotides were annealed and ligated into a dual-promoter lentiviral vector downstream of a U6 promoter that expresses Cre recombinase from the Pgk promoter. For Snail induction, the mice were fed diet containing 0.625 g/kg doxycycline (Provimi Kliba) from the day of tumor initiation until the day of sacrifice. The mice were sacrificed by intraperitoneal pentobarbital injection when they reached the endpoint, which was between 16 to 33 weeks post infection.

Individual KP lung tumors were dissected under a stereoscope to avoid contamination with healthy tissue and processed for downstream applications. Single cell suspensions from individual tumors were generated using a GentleMACS tissue octo dissociator (Miltenyi). Magnetic cell sorting to purify total immune cells was performed using Miltenyi anti-CD45 MicroBeads. Fluorescence activated cell sorting was performed using the MoFlow ASTRIOS EQ cell sorter. Before sorting, immune cells were enriched using CD45 magnetic isolation. Neutrophils (CD11b^+^ Ly6G^+^), monocytes (CD11b^+^, CD11c^−^, F4/80^−^, CD3^−^, B220^−^), T cells (CD3^+^), B cells (B220^+^, CD11c^−^), macrophages (CD11b^+/int^, F4/80^+^) and DCs (CD11c^+^ F4/80^−^, CD11b^+/int^, Ly6G^−^) were sorted simultaneously among CD45^+^ DAPI^−^ viable cells from the same sample. We have previously described these procedures in detail [5].

### Cell culture

The human NSCLC cell lines A549, NCI-H727 and NCI-H2122 were purchased from ATCC and cultured in RPMI medium. The murine cell lines M8 and SV2 were generated in our laboratory from single cell suspensions obtained each from a KP lung tumor and cultured for at least 25 passages before experimentation. All cell culture media were supplemented with 10 % fetal bovine serum (FBS) and 1 % PenStep at 21 % O_2_ and 5 % CO_2_. Mycoplasma tests using the Mycoplasma Detection Kit (SouthernBiotech) were performed regularly to ensure mycoplasma-free cell cultures. Where indicated, the cells were treated with 10 ng/mL recombinant human TGF-β2 (PeproTech), 20 or 100 ng/mL IL6 or TNF (Chimerigen Laboratories) for the described duration. The generation of stably transfected Snail-inducible cell lines has been described previously [5]. Primary splenocytes were derived from spleens from tumor-free mice by mechanical dissociation followed by red blood cell lysis (BD Pharm Lyse, BD Biosciences). The cells were counted and 4 × 10^6^ cells diluted in 500 μL complete DMEM medium.

The conditioned medium (CM) was generated from transduced cells that had been under doxycycline treatment for 7 days. To that end, 9×10^6^cells were plated into 15 cm culture dishes in 15 mL of complete medium without blasticidin with or without doxycycline for 48 h. The CM was collected, 0.22 μm filtered and aliquots stored at −80°C. The exosome preparations were generated according to a protocol adapted from Kowal *et al* [22]. Briefly, fresh CM was sequentially centrifuged for 5min at 500 x g, 5 min at 200 x g and 20 min at 4.600 g and the supernatant was each time transferred to a fresh tube. Finally, ultracentrifugation was performed at 26.000 rpm during 1 h 10 min using a 32 rotor. The supernatant was collected as “exosome-free CM” and the pellet, containing the exosomes, resuspended in PBS. Both preparations were aliquoted and stored at −80°C.

### Real time PCR analysis

Total cellular RNA was extracted using TRIzol reagent (Life Technologies) from individual tumors, cellular fractions or cultured cells. One μg of RNA was reverse-transcribed into cDNA using the High-Capacity Reverse Transcription Kit (Applied Biosystems). Real time PCR amplifications were performed using 10 ng of cDNA that were loaded onto 384-well plates using a Hamilton Microlab Star liquid handling platform and analyzed on QuantStudio or 7900HT Fast qPCR instruments. Taqman universal PCR master mix (Thermo Fisher Scientific) and commercially available Taqman probes (Applied Biosystems; the recommended version “Best Coverage”) were used. The comparative C_t_ method was used for data analysis [46]. Gene expression was normalized to *GAPDH* or *Rpl30* for human and mouse samples, respectively. The expression of mature miRNAs was determined using tailored Taqman Advanced miRNA cards. The samples were analyzed using Taqman Fast Advanced Master mix and Taqman Array Micro Fluidic Cards, preloaded with a customized assortment of Taqman Advanced miRNA Assays, including 37 miRNAs encoded by the Dlk1-Dio3 locus and the housekeeping miRNA hsa-miR-191-5p, recommended by Life Technologies, upon amplification on a 7900HT Fast qPCR instrument.

### Statistical data analysis

For all mouse experiments, we used a minimum of 3 mice per condition and n generally refers to the number of tumors analyzed, unless otherwise indicated. All results are represented as mean ± SEM if not stated otherwise. For *in vitro* experiments, n refers to the number of biological replicates. Comparisons between groups were made as stated in the figure legends. Statistical significance was indicated as ^*^ (p < 0.05), ^**^ (p < 0.01), ^***^ (p < 0.001), ^****^ (p < 0.0001) and ns: not significant, based on Mann-Whitney test, where not indicated otherwise. Statistical analysis was performed using Prism 7 software.

### Microarray analysis

Gene expression profiles of Snail overexpressing (4 samples overexpressing Snail and 4 control samples) and Snail knockdown (5 sh-Snail and 6 sh-control samples) individual tumors were measured on Affymetrix MoGene 2.0 ST Array. Raw expression data were normalized using RMA (oligo package). Filtering was applied to form a filtered set of genes: control probe sets and probe sets with mean log2 expression below 5 were removed. One probe set with highest variance was kept for each gene; uncharacterized genes were removed to form a filtered set of 18522 genes for Snail-KD and 16988 genes for Snail-OE experiment. Differential expression was computed using limma package [47].

### Data deposition

Microarrray data have been deposited in the public repository GEO (GSE110910).

### Gene set enrichment analysis and overrepresentation analysis

All genes located in the Dlk1-Dio3 locus were used as one gene set to perform Gene Set Enrichment Analysis [48] in Snail overexpression and Snail knockdown experiments. We investigated pathways as defined in the Hallmark biological process collection of MSigDB [48]. Overrepresentation analysis (ORA; using Fisher's exact test) was computed of pathways among the genes in the respective intersection.

## SUPPLEMENTARY MATERIALS FIGURES AND TABLES









## Abbreviations

CLL: chronic lymphocytic leukemia
CM: conditioned medium
COPD: chronic obstructive pulmonary disease
DC: dendritic cell
DIO3: thyroxine 5-deiodinase
DLK1: delta-like homolog
DMNT3: DNA (cytosine-5)-methyltransferase 3B
EMT: epithelial-to-mesenchymal transition
EZH2: enhancer of zeste homolog 2
Gtl2: gene trap locus 2
ICR: imprinting control region
Ig-DMR: intergenic differentially methylated region
KD: knockdown
KPR: *Kras^LSL-G12D/+^*; *p53^fl/fl^*; *CCSP-rtTA*
lncRNA: long non-coding RNA
LOI: loss of imprinting
LSD1: Lysine-specific histone demethylase 1A
MEG: maternally expressed gene
miRNA: micro RNA
ncRNA: non-coding RNA
NSCLC: non-small cell lung cancer
OE: overexpression
ORA: overrepresentation analysis
piRNA: PIWI-interacting RNA
PRC2: polycomb repressive complex 2
PBMC: peripheral blood mononuclear cells
Rian: RNA imprinted and accumulated in nucleus
RTL1: retrotransposon-like protein 1
snoRNA: small nucleolar RNA
TCGA: The Cancer Genome Atlas
TNF: tumor necrosis factor
