# Supplementary material for: Snail mediates repression of the Dlk1-Dio3 locus in lung tumor-infiltrating immune cells
Source: Oncotarget. 2018 Aug 17;9(64):32331–45. doi: 10.18632/oncotarget.25965 (PMC6122344; doi:10.18632/oncotarget.25965)
Supplement: Supplementary file 1 [file oncotarget-09-32331-s001.pdf]

Snail mediates repression of the Dlk1-Dio3 locus in lung tumor-infiltrating immune cells

SUPPLEMENTARY MATERIALS

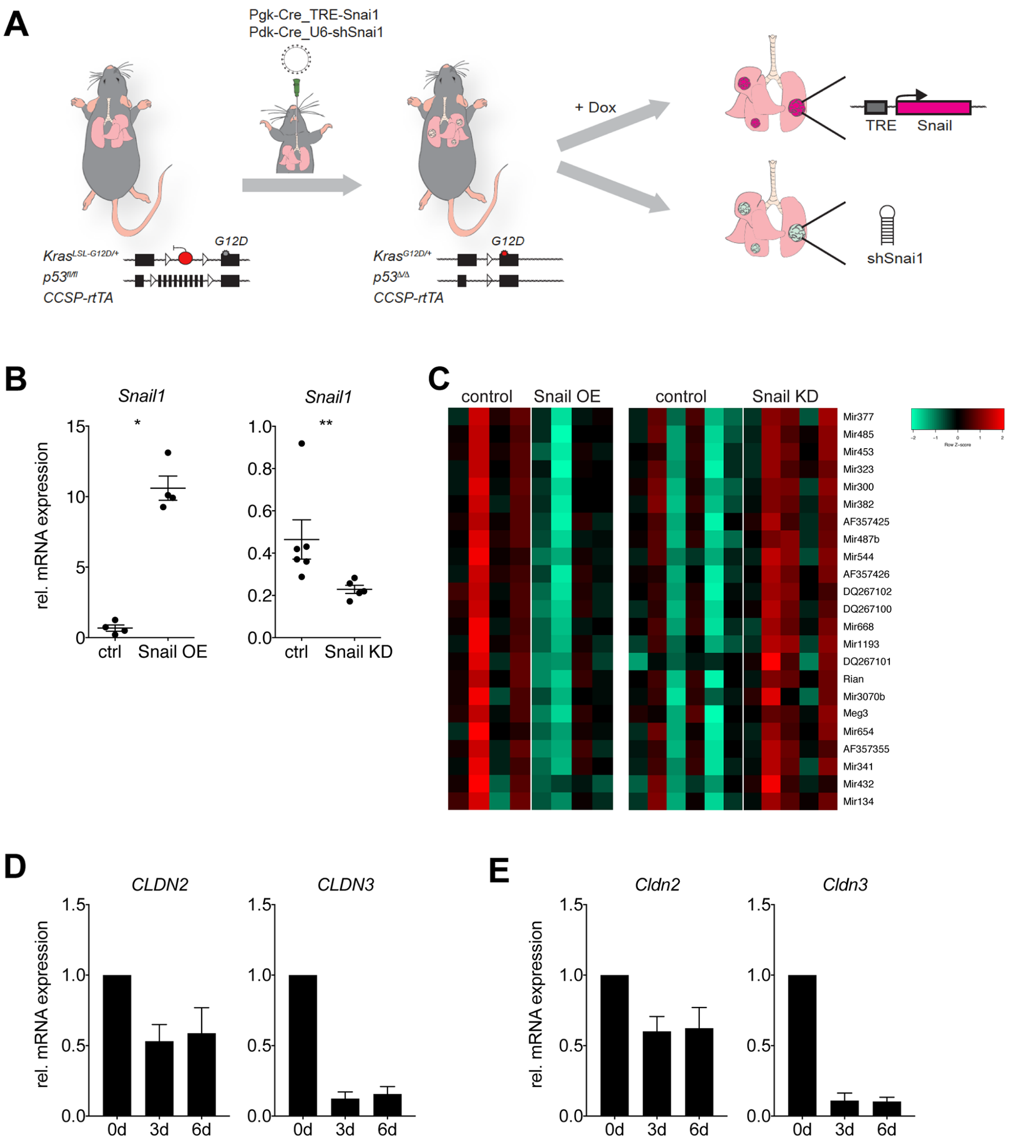

Supplementary Figure 1: The Dlk1-Dio3 locus is downregulated in Snail OE and upregulated in Snail KD tumors. (A) Experimental scheme of Snail overexpression (OE) and knockdown (KD) in the KP lung adenocarcinoma model. Lentiviral vectors were used to facilitate doxycycline-induced Snail overexpression or silencing of Snail mediated by constitutive shRNA expression in the KP lung tumors. **(B)** Dot plots represent Snai1 mRNA expression in the *(left)* Snail OE and *(right)* Snail KD tumors, which were individually dissected and analysed by microarray. **(C)** Heatmaps depicting the expression levels of the genes located within the Dlk1-Dio3 locus, which represent 23 % of the "Snail repressed" genes downregulated in individual Snail overexpressing (n = 4) compared to control (n = 4) tumors and upregulated in individual Snail knockdown (n = 5) relative to control (n = 6) tumors, based on the microarray analysis. *OE*: overexpression, *KD*: knockdown. **(D)** Real time PCR analysis of the mRNA expression of *CLDN2* and *3*, normalized to *GAPDH*, in the Snail-inducible human NSCLC cell line H2122 treated for 0, 3 or 6 days with doxycycline, relative to the non-induced condition (n = 3). **(E)** Real time PCR analysis of the mRNA expression of *Cldn2* and *3*, normalized to *Rpl30*, in the Snail-inducible murine lung adenocarcinoma cell line SV2 treated for 0, 3 or 6 days with doxycycline, relative to the non-induced condition (n = 3).

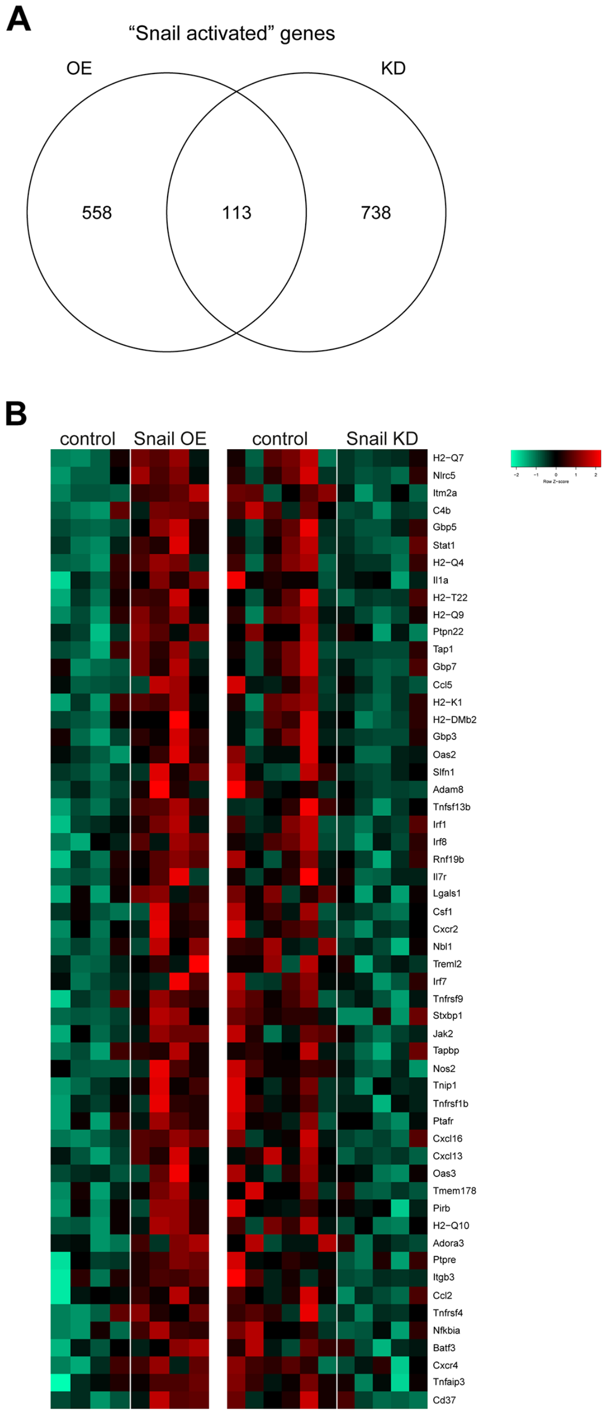

Supplementary Figure 2: “Snail activated” genes contain numerous proinflammatory genes. (A) Venn diagrams representing the intersection between differentially expressed genes (p-value < 0.1), upregulated (log fold change > 0) in individual Snail overexpressing (n = 4) compared to control (n = 4) tumors and downregulated (log fold change < 0) in individual Snail knockdown (n = 5) relative to control (n = 6) tumors, containing “Snail activated” genes, based on microarray analysis. **(B)** Heatmaps depicting the expression levels of the genes annotated with the GO term “immunological process”, including many genes involved in interferon and TNF signaling, which represent 49 % of the “Snail activated” genes upregulated in individual Snail overexpressing (n = 4) compared to control (n = 4) tumors and downregulated in individual Snail knockdown (n = 5) relative to control (n = 6) tumors, based on microarray analysis. *OE*: overexpression, *KD*: knockdown.

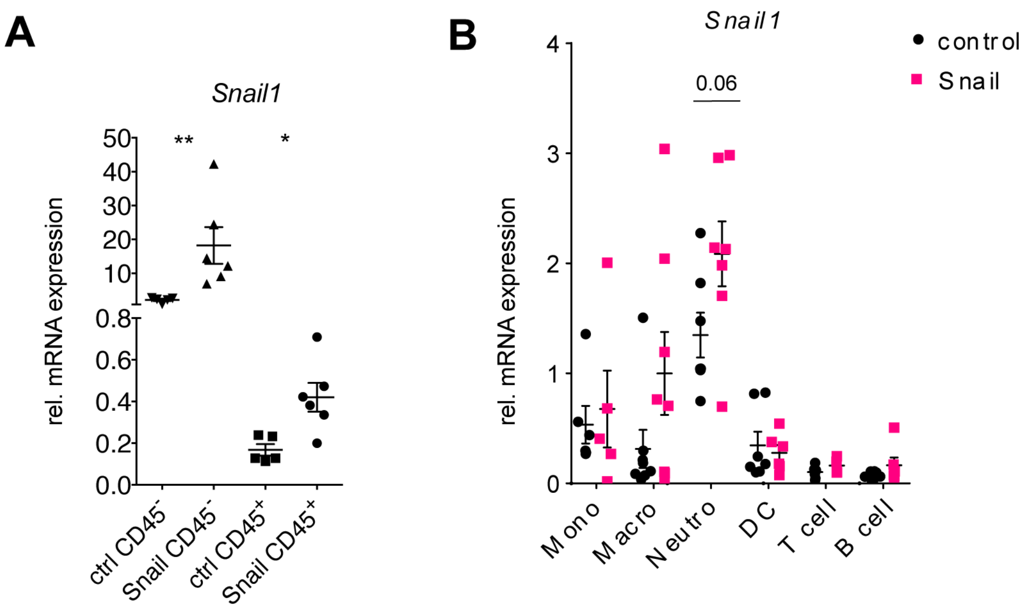

Supplementary Figure 3: Snail expression in lung tumor infiltrating immune cells. Real time PCR analysis of mRNA expression of *Snai1* in **(A)** CD45$^-$ tumor (ctrl: n = 5, Snail: n = 6) and CD45$^+$ immune (ctrl: n = 5, Snail: n = 6) cell fractions isolated from individual control KP and Snail overexpressing tumors using magnetic beads and **(B)** in six immune populations, i.e. monocytes (Mono), macrophages (Macro), neutrophils (Neutro), dendritic cells (DC), T and B lymphocytes (T and B cell, respectively), that were FACS-sorted from individual control KP (n = 11) and Snail overexpressing KPR (n = 8). (A, B) Graphs show mean with SEM. Statistical analysis based on (A) Mann-Whitney test and (B) Multiple *t*-tests: *: $p < 0.05$; not indicated: not significant.

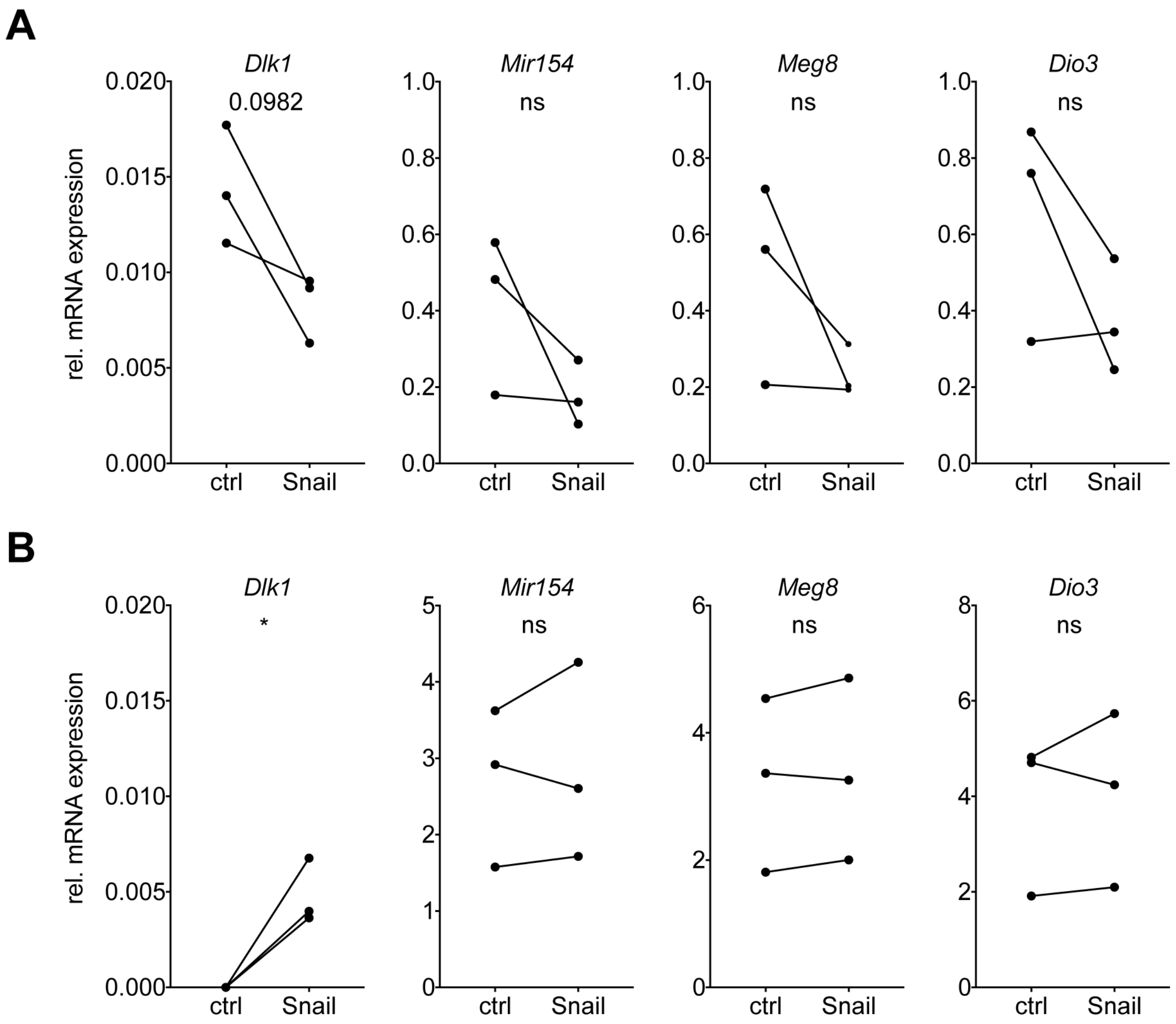

Supplementary Figure 4: Exosomes are not required in Snail-mediated Dlk1-Dio3 locus repression. Real time PCR analysis of mRNA expression of *Dlk1, Mir154, Meg8* and *Dio3* in primary splenocytes isolated from healthy mice and incubated during 48 h with **(A)** CM from control or Snail overexpressing H2122 cells, which had been subjected to sequential centrifugation to remove exosomes or **(B)** fresh medium containing the in (A) isolated exosomes diluted to a 5X concentration. Graphs show mean with SEM. Statistical analysis based on paired *t*-test: *: $p < 0.05$, ns: not significant.

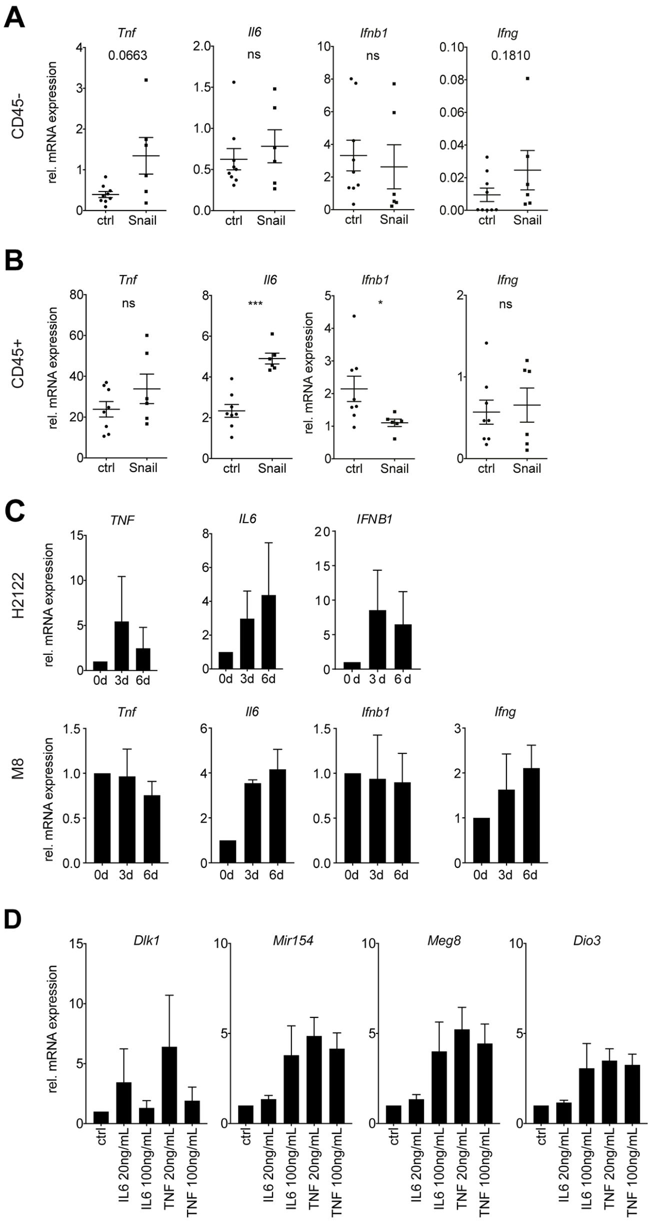

Supplementary Figure 5: IL6 and TNF do not downregulate the Dlk1-Dio3 locus in immune cells. Real time PCR analysis of the mRNA expression of **(A, B)** *Il6* and *Tnf*, normalized to *Rpl30*, in (A) $CD45^-$ tumor and (B) $CD45^+$ immune cell fractions isolated from control KP (n = 9) and Snail overexpressing (n = 6) tumors using magnetic beads. **(C)** Real time PCR analysis of the mRNA expression of *IL6, TNF*, *IFNB1* and *IFNG*, normalized to *GAPDH* or *Rpl30*, in the Snail-inducible human NSCLC cell line H2122 or the murine KP lung adenocarcinoma cell line M8, treated for 0, 3 or 6 days with doxycycline, relative to the non-induced condition (n = 3). **(D)** Real time PCR analysis of mRNA expression of *Dlk1, Mir154, Meg8* and *Dio3* in primary murine splenocytes, untreated or treated *ex vivo* with IL6 or TNF (20 or 100 ng/mL) for 24 hours. Graphs show mean with SEM. (A,B) Statistical analysis based on Mann-Whitney test: ***: $p < 0.001$, ns: not significant.

Supplementary Table 1: List of 602 downregulated genes in Snail OE tumors from Figure 1A.

See Supplementary File 1

Supplementary Table 2: List of 830 upregulated genes in Snail KD tumors from Figure 1A.

See Supplementary File 2

Supplementary Table 3: List of 114 genes in the intersection of “Snail repressed” genes from Figure 1A.

See Supplementary File 3

Supplementary Table 4: Overrepresented pathways from the Hallmark collection among the genes of the intersection of “Snail repressed” genes (n = 114)

Gene Set	p-value	FDR
G2M CHECKPOINT	2.01E-02	1
FATTY ACID METABOLISM	4.87E-02	1
PEROXISOME	1.01E-01	1
MITOTIC SPINDLE	2.77E-01	1
ESTROGEN RESPONSE EARLY	2.77E-01	1
COMPLEMENT	2.77E-01	1
E2F TARGETS	2.77E-01	1
MYC TARGETS V1	2.77E-01	1
GLYCOLYSIS	2.77E-01	1
IL2 STAT5 SIGNALING	2.77E-01	1
CHOLESTEROL HOMEOSTASIS	3.19E-01	1
PROTEIN SECRETION	3.93E-01	1

FDR: false discovery rate.
