# Supplementary material for: Snail mediates repression of the Dlk1-Dio3 locus in lung tumor-infiltrating immune cells
Source: Oncotarget. 2018 Aug 17;9(64):32331–45. doi: 10.18632/oncotarget.25965 (PMC6122344; doi:10.18632/oncotarget.25965)
Supplement: Supplementary file 2 [file oncotarget-09-32331-s002.docx]

**Supplementary Table 1: List of 602 downregulated genes in Snail OE tumors from Figure 1A**

| **Symbol** | **Description** | **fc** | **p-value** |
| --- | --- | --- | --- |
| Xist | inactive X specific transcripts | -48.4 | 5.00E-03 |
| Cym | chymosin | -4.4 | 1.60E-02 |
| Tmem27 | transmembrane protein 27 | -4 | 6.00E-03 |
| Trpv6 | transient receptor potential cation channel, subfamily V, member 6 | -3.4 | 1.20E-02 |
| Gdpd2 | glycerophosphodiester phosphodiesterase domain containing 2 | -3.2 | 7.70E-03 |
| Tmed6 | transmembrane emp24 protein transport domain containing 6 | -3.1 | 5.60E-03 |
| Mir380 | microRNA 380 | -3 | 2.90E-02 |
| P2rx2 | purinergic receptor P2X, ligand-gated ion channel, 2 | -2.9 | 1.50E-02 |
| Dlk1 | delta-like 1 homolog (Drosophila) | -2.9 | 3.70E-02 |
| Mir154 | microRNA 154 | -2.8 | 2.40E-02 |
| Mir376b | microRNA 376b | -2.8 | 5.00E-02 |
| Cpn1 | carboxypeptidase N, polypeptide 1 | -2.7 | 1.40E-02 |
| Mir1927 | microRNA 1927 | -2.7 | 2.80E-02 |
| Mir543 | microRNA 543 | -2.6 | 1.10E-02 |
| Mirg | miRNA containing gene | -2.6 | 5.10E-02 |
| Bex4 | brain expressed gene 4 | -2.5 | 4.60E-03 |
| Entpd3 | ectonucleoside triphosphate diphosphohydrolase 3 | -2.5 | 7.90E-04 |
| Foxp2 | forkhead box P2 | -2.4 | 2.50E-04 |
| Cldn6 | claudin 6 | -2.4 | 2.40E-02 |
| Atp6v0e2 | ATPase, H+ transporting, lysosomal V0 subunit E2 | -2.4 | 8.50E-04 |
| Gpc3 | glypican 3 | -2.4 | 2.80E-02 |
| Mir667 | microRNA 667 | -2.3 | 8.00E-03 |
| Crlf1 | cytokine receptor-like factor 1 | -2.3 | 1.70E-02 |
| Mir329 | microRNA 329 | -2.3 | 4.80E-02 |
| Hecw1 | HECT, C2 and WW domain containing E3 ubiquitin protein ligase 1 | -2.3 | 1.60E-02 |
| Shh | sonic hedgehog | -2.3 | 3.20E-02 |
| Gpc6 | glypican 6 | -2.2 | 1.80E-02 |
| Tdrkh | tudor and KH domain containing protein | -2.2 | 1.60E-02 |
| Scel | sciellin | -2.2 | 3.70E-02 |
| Nrk | Nik related kinase | -2.2 | 6.40E-04 |
| Tmeff2 | transmembrane protein with EGF-like and two follistatin-like domains 2 | -2.2 | 8.10E-03 |
| Vil1 | villin 1 | -2.2 | 4.10E-02 |
| BC068157 | cDNA sequence BC068157 | -2.1 | 3.40E-03 |
| Rerg | RAS-like, estrogen-regulated, growth-inhibitor | -2.1 | 2.90E-02 |
| Slc15a1 | solute carrier family 15 (oligopeptide transporter), member 1 | -2.1 | 3.60E-02 |
| Cpm | carboxypeptidase M | -2.1 | 2.10E-02 |
| Ros1 | Ros1 proto-oncogene | -2.1 | 7.80E-02 |
| C87198 | expressed sequence C87198 | -2 | 9.00E-03 |
| Veph1 | ventricular zone expressed PH domain-containing 1 | -2 | 2.90E-02 |
| Ppp1r9a | protein phosphatase 1, regulatory (inhibitor) subunit 9A | -2 | 1.80E-02 |
| Mir496a | microRNA 496a | -2 | 4.70E-02 |
| LOC102632404 | uncharacterized LOC102632404 | -2 | 6.30E-02 |
| Zmat4 | zinc finger, matrin type 4 | -2 | 6.20E-02 |
| Slc4a5 | solute carrier family 4, sodium bicarbonate cotransporter, member 5 | -2 | 6.20E-03 |
| Jpx | Jpx transcript, Xist activator (non-protein coding) | -2 | 1.20E-03 |
| Sgce | sarcoglycan, epsilon | -2 | 2.60E-02 |
| BC026585 | cDNA sequence BC026585 | -2 | 2.20E-03 |
| Mir1188 | microRNA 1188 | -2 | 7.40E-02 |
| Slc1a1 | solute carrier family 1 (neuronal/epithelial high affinity glutamate transporter, system Xag), member 1 | -2 | 7.80E-02 |
| Mir758 | microRNA 758 | -1.9 | 2.80E-02 |
| Lgi3 | leucine-rich repeat LGI family, member 3 | -1.9 | 2.90E-02 |
| Chst4 | carbohydrate (chondroitin 6/keratan) sulfotransferase 4 | -1.9 | 8.80E-02 |
| Slain1 | SLAIN motif family, member 1 | -1.9 | 3.20E-02 |
| Mir679 | microRNA 679 | -1.9 | 3.50E-02 |
| Trbj2-3 | T cell receptor beta joining 2-3 | -1.9 | 7.80E-02 |
| Ephb2 | Eph receptor B2 | -1.9 | 4.70E-02 |
| Kdm6a | lysine (K)-specific demethylase 6A | -1.9 | 1.10E-02 |
| Slc23a3 | solute carrier family 23 (nucleobase transporters), member 3 | -1.9 | 8.20E-03 |
| Tinag | tubulointerstitial nephritis antigen | -1.9 | 3.10E-02 |
| Peg3 | paternally expressed 3 | -1.9 | 3.40E-02 |
| Hgfac | hepatocyte growth factor activator | -1.9 | 3.40E-03 |
| Mvb12b | multivesicular body subunit 12B | -1.9 | 5.70E-03 |
| Fmnl2 | formin-like 2 | -1.9 | 9.90E-02 |
| Syne1 | spectrin repeat containing, nuclear envelope 1 | -1.9 | 9.90E-02 |
| D10Bwg1379e | DNA segment, Chr 10, Brigham & Womens Genetics 1379 expressed | -1.9 | 6.00E-02 |
| Egfl6 | EGF-like-domain, multiple 6 | -1.9 | 7.00E-02 |
| Atp6v1c2 | ATPase, H+ transporting, lysosomal V1 subunit C2 | -1.9 | 4.30E-02 |
| Klhdc8b | kelch domain containing 8B | -1.8 | 2.40E-03 |
| Ctnnd2 | catenin (cadherin associated protein), delta 2 | -1.8 | 7.90E-03 |
| Mycn | v-myc myelocytomatosis viral related oncogene, neuroblastoma derived (avian) | -1.8 | 7.70E-02 |
| Acsl3 | acyl-CoA synthetase long-chain family member 3 | -1.8 | 3.90E-02 |
| Tmem59l | transmembrane protein 59-like | -1.8 | 1.30E-02 |
| Upk1a | uroplakin 1A | -1.8 | 2.20E-02 |
| Rhox4e | reproductive homeobox 4E | -1.8 | 5.90E-02 |
| Liph | lipase, member H | -1.8 | 4.80E-02 |
| Duxbl2 | doubl homeobox B-like 2 | -1.8 | 1.90E-03 |
| Vsig1 | V-set and immunoglobulin domain containing 1 | -1.8 | 5.40E-02 |
| Trabd2b | TraB domain containing 2B | -1.8 | 3.50E-03 |
| Gja3 | gap junction protein, alpha 3 | -1.8 | 1.50E-04 |
| Apoa1 | apolipoprotein A-I | -1.8 | 9.80E-02 |
| Rab15 | RAB15, member RAS oncogene family | -1.8 | 8.70E-02 |
| Mir665 | microRNA 665 | -1.8 | 2.40E-02 |
| Slc16a12 | solute carrier family 16 (monocarboxylic acid transporters), member 12 | -1.8 | 8.80E-02 |
| Rec8 | REC8 meiotic recombination protein | -1.8 | 4.30E-02 |
| Rab27a | RAB27A, member RAS oncogene family | -1.8 | 5.70E-02 |
| Syne4 | spectrin repeat containing, nuclear envelope family member 4 | -1.8 | 5.40E-02 |
| Pdgfd | platelet-derived growth factor, D polypeptide | -1.8 | 3.30E-03 |
| Fat2 | FAT tumor suppressor homolog 2 (Drosophila) | -1.8 | 3.60E-02 |
| Sel1l3 | sel-1 suppressor of lin-12-like 3 (C. elegans) | -1.8 | 5.20E-02 |
| Clic6 | chloride intracellular channel 6 | -1.8 | 7.50E-02 |
| Kcnc3 | potassium voltage gated channel, Shaw-related subfamily, member 3 | -1.8 | 1.60E-02 |
| Grhl1 | grainyhead-like 1 (Drosophila) | -1.7 | 1.20E-02 |
| Plxna3 | plexin A3 | -1.7 | 1.40E-03 |
| Mir532 | microRNA 532 | -1.7 | 2.20E-02 |
| Amot | angiomotin | -1.7 | 8.70E-03 |
| Myo5c | myosin VC | -1.7 | 4.20E-02 |
| Eif2s3x | eukaryotic translation initiation factor 2, subunit 3, structural gene X-linked | -1.7 | 2.20E-02 |
| Id2 | inhibitor of DNA binding 2 | -1.7 | 4.50E-02 |
| Dtna | dystrobrevin alpha | -1.7 | 3.50E-02 |
| Fstl4 | follistatin-like 4 | -1.7 | 6.80E-02 |
| St14 | suppression of tumorigenicity 14 (colon carcinoma) | -1.7 | 8.70E-03 |
| Ildr1 | immunoglobulin-like domain containing receptor 1 | -1.7 | 3.30E-02 |
| Pcyox1l | prenylcysteine oxidase 1 like | -1.7 | 3.80E-03 |
| Tlr5 | toll-like receptor 5 | -1.7 | 4.40E-02 |
| Ap1m2 | adaptor protein complex AP-1, mu 2 subunit | -1.7 | 7.10E-02 |
| Chrnb1 | cholinergic receptor, nicotinic, beta polypeptide 1 (muscle) | -1.7 | 5.90E-03 |
| Adam4 | a disintegrin and metallopeptidase domain 4 | -1.7 | 5.30E-02 |
| Parm1 | prostate androgen-regulated mucin-like protein 1 | -1.7 | 2.50E-02 |
| Ttll10 | tubulin tyrosine ligase-like family, member 10 | -1.7 | 1.70E-02 |
| Vcpkmt | valosin containing protein lysine (K) methyltransferase | -1.7 | 3.50E-02 |
| Epha4 | Eph receptor A4 | -1.7 | 3.00E-03 |
| Swsap1 | SWIM type zinc finger 7 associated protein 1 | -1.7 | 6.20E-02 |
| Efna5 | ephrin A5 | -1.7 | 2.70E-03 |
| Slc26a9 | solute carrier family 26, member 9 | -1.6 | 5.60E-02 |
| Kdm5c | lysine (K)-specific demethylase 5C | -1.6 | 8.50E-04 |
| Dpep2 | dipeptidase 2 | -1.6 | 5.40E-02 |
| Mansc1 | MANSC domain containing 1 | -1.6 | 1.40E-02 |
| Samd5 | sterile alpha motif domain containing 5 | -1.6 | 2.00E-02 |
| Zfp266 | zinc finger protein 266 | -1.6 | 4.90E-02 |
| Lrrc8b | leucine rich repeat containing 8 family, member B | -1.6 | 1.60E-02 |
| Kif4 | kinesin family member 4 | -1.6 | 1.10E-02 |
| Celsr1 | cadherin, EGF LAG seven-pass G-type receptor 1 (flamingo homolog, Drosophila) | -1.6 | 3.40E-02 |
| Sec16b | SEC16 homolog B (S. cerevisiae) | -1.6 | 4.30E-02 |
| Gramd1b | GRAM domain containing 1B | -1.6 | 3.70E-02 |
| Ftx | Ftx transcript, Xist regulator (non-protein coding) | -1.6 | 6.20E-03 |
| Mir717 | microRNA 717 | -1.6 | 2.30E-02 |
| Alpk3 | alpha-kinase 3 | -1.6 | 5.40E-03 |
| Rps6ka6 | ribosomal protein S6 kinase polypeptide 6 | -1.6 | 2.20E-02 |
| Grik5 | glutamate receptor, ionotropic, kainate 5 (gamma 2) | -1.6 | 1.20E-02 |
| Scnn1g | sodium channel, nonvoltage-gated 1 gamma | -1.6 | 3.60E-02 |
| Tekt5 | tektin 5 | -1.6 | 2.80E-02 |
| Aif1l | allograft inflammatory factor 1-like | -1.6 | 3.80E-02 |
| Bmp4 | bone morphogenetic protein 4 | -1.6 | 2.80E-02 |
| Tenm4 | teneurin transmembrane protein 4 | -1.6 | 4.90E-02 |
| Ralgps2 | Ral GEF with PH domain and SH3 binding motif 2 | -1.6 | 5.60E-02 |
| Neurl1b | neuralized homolog 1b (Drosophila) | -1.6 | 6.80E-03 |
| H1fx | H1 histone family, member X | -1.6 | 4.20E-03 |
| Zdhhc23 | zinc finger, DHHC domain containing 23 | -1.6 | 9.10E-03 |
| Pon3 | paraoxonase 3 | -1.6 | 6.20E-02 |
| Sorcs2 | sortilin-related VPS10 domain containing receptor 2 | -1.6 | 3.60E-02 |
| Pid1 | phosphotyrosine interaction domain containing 1 | -1.6 | 5.50E-03 |
| Sel1l | sel-1 suppressor of lin-12-like (C. elegans) | -1.6 | 4.90E-02 |
| Mpzl1 | myelin protein zero-like 1 | -1.6 | 7.60E-02 |
| Slc24a4 | solute carrier family 24 (sodium/potassium/calcium exchanger), member 4 | -1.6 | 9.00E-02 |
| Sorl1 | sortilin-related receptor, LDLR class A repeats-containing | -1.6 | 5.80E-02 |
| Kif23 | kinesin family member 23 | -1.6 | 9.70E-02 |
| Peg10 | paternally expressed 10 | -1.6 | 3.20E-02 |
| Ccnb2 | cyclin B2 | -1.6 | 5.50E-02 |
| Phxr4 | per-hexamer repeat gene 4 | -1.6 | 9.40E-03 |
| Zfp579 | zinc finger protein 579 | -1.6 | 1.40E-03 |
| Tmem139 | transmembrane protein 139 | -1.6 | 5.30E-02 |
| Arhgap6 | Rho GTPase activating protein 6 | -1.6 | 1.40E-02 |
| Atp2a3 | ATPase, Ca++ transporting, ubiquitous | -1.6 | 4.50E-02 |
| Lrcol1 | leucine rich colipase-like 1 | -1.6 | 2.50E-02 |
| Tspan7 | tetraspanin 7 | -1.6 | 1.20E-03 |
| LOC102639184 | uncharacterized LOC102639184 | -1.5 | 4.40E-02 |
| Hsbp1l1 | heat shock factor binding protein 1-like 1 | -1.5 | 1.20E-02 |
| Scnn1b | sodium channel, nonvoltage-gated 1 beta | -1.5 | 4.90E-02 |
| Mir494 | microRNA 494 | -1.5 | 7.20E-03 |
| Kif3c | kinesin family member 3C | -1.5 | 2.40E-02 |
| Zxdb | zinc finger, X-linked, duplicated B | -1.5 | 2.40E-02 |
| Bicd1 | bicaudal D homolog 1 (Drosophila) | -1.5 | 8.90E-02 |
| Mpp7 | membrane protein, palmitoylated 7 (MAGUK p55 subfamily member 7) | -1.5 | 7.20E-02 |
| Cacna1h | calcium channel, voltage-dependent, T type, alpha 1H subunit | -1.5 | 5.60E-02 |
| Noxo1 | NADPH oxidase organizer 1 | -1.5 | 5.90E-02 |
| Slc37a1 | solute carrier family 37 (glycerol-3-phosphate transporter), member 1 | -1.5 | 4.50E-02 |
| Ggcx | gamma-glutamyl carboxylase | -1.5 | 1.20E-02 |
| Igfbp5 | insulin-like growth factor binding protein 5 | -1.5 | 6.70E-02 |
| Exosc7 | exosome component 7 | -1.5 | 5.80E-02 |
| Stmn1 | stathmin 1 | -1.5 | 5.70E-02 |
| Pigb | phosphatidylinositol glycan anchor biosynthesis, class B | -1.5 | 9.10E-02 |
| Kif15 | kinesin family member 15 | -1.5 | 9.80E-02 |
| Stk39 | serine/threonine kinase 39 | -1.5 | 3.50E-02 |
| Pm20d1 | peptidase M20 domain containing 1 | -1.5 | 6.70E-02 |
| Cers6 | ceramide synthase 6 | -1.5 | 7.50E-02 |
| Epcam | epithelial cell adhesion molecule | -1.5 | 1.30E-02 |
| Spint1 | serine protease inhibitor, Kunitz type 1 | -1.5 | 2.80E-02 |
| Pdk3 | pyruvate dehydrogenase kinase, isoenzyme 3 | -1.5 | 7.50E-02 |
| Bcam | basal cell adhesion molecule | -1.5 | 2.40E-02 |
| Lpcat1 | lysophosphatidylcholine acyltransferase 1 | -1.5 | 9.00E-02 |
| Pkhd1 | polycystic kidney and hepatic disease 1 | -1.5 | 7.00E-02 |
| Chrnb2 | cholinergic receptor, nicotinic, beta polypeptide 2 (neuronal) | -1.5 | 2.80E-02 |
| Pus3 | pseudouridine synthase 3 | -1.5 | 7.60E-02 |
| Dynlt3 | dynein light chain Tctex-type 3 | -1.5 | 7.30E-02 |
| Zbtb38 | zinc finger and BTB domain containing 38 | -1.5 | 2.30E-02 |
| Filip1 | filamin A interacting protein 1 | -1.5 | 5.20E-02 |
| Nlrp4c | NLR family, pyrin domain containing 4C | -1.5 | 9.00E-02 |
| Adamts18 | a disintegrin-like and metallopeptidase (reprolysin type) with thrombospondin type 1 motif, 18 | -1.5 | 8.50E-02 |
| Alg9 | asparagine-linked glycosylation 9 (alpha 1,2 mannosyltransferase) | -1.5 | 9.10E-02 |
| Lyplal1 | lysophospholipase-like 1 | -1.5 | 1.10E-02 |
| Prokr1 | prokineticin receptor 1 | -1.5 | 3.40E-03 |
| Smagp | small cell adhesion glycoprotein | -1.5 | 4.60E-02 |
| Akap5 | A kinase (PRKA) anchor protein 5 | -1.5 | 4.90E-02 |
| Ercc6 | excision repair cross-complementing rodent repair deficiency, complementation group 6 | -1.5 | 4.00E-02 |
| Haus1 | HAUS augmin-like complex, subunit 1 | -1.5 | 6.50E-02 |
| F8a | factor 8-associated gene A | -1.5 | 1.60E-02 |
| Prnp | prion protein | -1.5 | 3.10E-02 |
| Fam213a | family with sequence similarity 213, member A | -1.5 | 4.20E-02 |
| Plk1 | polo-like kinase 1 | -1.5 | 6.20E-02 |
| Tagap1 | T cell activation GTPase activating protein 1 | -1.5 | 7.70E-03 |
| Sh3yl1 | Sh3 domain YSC-like 1 | -1.5 | 9.00E-02 |
| Fam120c | family with sequence similarity 120, member C | -1.5 | 1.10E-02 |
| Zc3hav1l | zinc finger CCCH-type, antiviral 1-like | -1.5 | 8.60E-02 |
| Adamts6 | a disintegrin-like and metallopeptidase (reprolysin type) with thrombospondin type 1 motif, 6 | -1.5 | 7.80E-02 |
| Bspry | B-box and SPRY domain containing | -1.5 | 4.90E-02 |
| Gjb2 | gap junction protein, beta 2 | -1.5 | 1.40E-02 |
| Ints7 | integrator complex subunit 7 | -1.5 | 2.60E-02 |
| Dtnb | dystrobrevin, beta | -1.5 | 9.40E-03 |
| Ovgp1 | oviductal glycoprotein 1 | -1.5 | 9.20E-02 |
| Ofd1 | oral-facial-digital syndrome 1 gene homolog (human) | -1.5 | 2.80E-02 |
| Prep | prolyl endopeptidase | -1.5 | 6.00E-02 |
| Klk14 | kallikrein related-peptidase 14 | -1.5 | 8.70E-02 |
| LOC102640749 | uncharacterized LOC102640749 | -1.5 | 4.40E-03 |
| Tnfaip8l1 | tumor necrosis factor, alpha-induced protein 8-like 1 | -1.5 | 4.30E-03 |
| Tmem164 | transmembrane protein 164 | -1.5 | 3.50E-02 |
| Chad | chondroadherin | -1.5 | 4.70E-02 |
| Nrg1 | neuregulin 1 | -1.5 | 1.70E-02 |
| St8sia6 | ST8 alpha-N-acetyl-neuraminide alpha-2,8-sialyltransferase 6 | -1.5 | 1.80E-02 |
| Tram2 | translocating chain-associating membrane protein 2 | -1.5 | 2.80E-02 |
| Rtkn2 | rhotekin 2 | -1.5 | 5.20E-02 |
| Cldn18 | claudin 18 | -1.5 | 7.10E-02 |
| Myct1 | myc target 1 | -1.5 | 1.00E-01 |
| Ap4s1 | adaptor-related protein complex AP-4, sigma 1 | -1.5 | 5.50E-02 |
| Ascl4 | achaete-scute complex homolog 4 (Drosophila) | -1.5 | 1.50E-02 |
| Fam71e1 | family with sequence similarity 71, member E1 | -1.5 | 6.60E-03 |
| Snai3 | snail family zinc finger 3 | -1.5 | 5.10E-02 |
| Tigd2 | tigger transposable element derived 2 | -1.5 | 9.10E-02 |
| Mthfs | 5, 10-methenyltetrahydrofolate synthetase | -1.5 | 6.90E-02 |
| Zfp185 | zinc finger protein 185 | -1.5 | 7.90E-02 |
| Kantr | Kdm5c adjacent non-coding transcript | -1.5 | 1.60E-02 |
| Zfp462 | zinc finger protein 462 | -1.5 | 6.40E-02 |
| Las1l | LAS1-like (S. cerevisiae) | -1.5 | 6.10E-02 |
| B4galt6 | UDP-Gal:betaGlcNAc beta 1,4-galactosyltransferase, polypeptide 6 | -1.5 | 1.40E-02 |
| Wnk3 | WNK lysine deficient protein kinase 3 | -1.5 | 9.10E-02 |
| Lamb2 | laminin, beta 2 | -1.5 | 8.20E-03 |
| Chd7 | chromodomain helicase DNA binding protein 7 | -1.5 | 1.30E-02 |
| Nkx2-1 | NK2 homeobox 1 | -1.5 | 1.50E-02 |
| Dpt | dermatopontin | -1.5 | 4.10E-02 |
| Rasgef1a | RasGEF domain family, member 1A | -1.5 | 3.60E-02 |
| Akr7a5 | aldo-keto reductase family 7, member A5 (aflatoxin aldehyde reductase) | -1.5 | 5.20E-03 |
| Zmat1 | zinc finger, matrin type 1 | -1.5 | 5.40E-03 |
| Cryab | crystallin, alpha B | -1.5 | 4.00E-02 |
| Atp13a5 | ATPase type 13A5 | -1.5 | 6.90E-02 |
| BC065397 | cDNA sequence BC065397 | -1.4 | 1.30E-02 |
| Fhit | fragile histidine triad gene | -1.4 | 1.70E-02 |
| Fam187b | family with sequence similarity 187, member B | -1.4 | 5.90E-03 |
| Plekhh1 | pleckstrin homology domain containing, family H (with MyTH4 domain) member 1 | -1.4 | 8.70E-02 |
| Mageh1 | melanoma antigen, family H, 1 | -1.4 | 8.20E-02 |
| Pank1 | pantothenate kinase 1 | -1.4 | 6.50E-02 |
| Casp2 | caspase 2 | -1.4 | 7.50E-02 |
| Kcnk9 | potassium channel, subfamily K, member 9 | -1.4 | 8.80E-03 |
| Plxna4 | plexin A4 | -1.4 | 2.30E-02 |
| Nrgn | neurogranin | -1.4 | 8.10E-02 |
| Creb3l1 | cAMP responsive element binding protein 3-like 1 | -1.4 | 9.70E-02 |
| Map3k9 | mitogen-activated protein kinase kinase kinase 9 | -1.4 | 6.40E-02 |
| Ndufb11 | NADH dehydrogenase (ubiquinone) 1 beta subcomplex, 11 | -1.4 | 6.60E-03 |
| Sos2 | son of sevenless homolog 2 (Drosophila) | -1.4 | 7.00E-02 |
| Npw | neuropeptide W | -1.4 | 4.80E-02 |
| Slc13a2 | solute carrier family 13 (sodium-dependent dicarboxylate transporter), member 2 | -1.4 | 5.60E-02 |
| Pbdc1 | polysaccharide biosynthesis domain containing 1 | -1.4 | 2.80E-02 |
| Ocrl | oculocerebrorenal syndrome of Lowe | -1.4 | 3.80E-02 |
| Reep4 | receptor accessory protein 4 | -1.4 | 2.50E-02 |
| Ezh2 | enhancer of zeste homolog 2 (Drosophila) | -1.4 | 7.00E-02 |
| Ebp | phenylalkylamine Ca2+ antagonist (emopamil) binding protein | -1.4 | 1.50E-02 |
| Kcnip2 | Kv channel-interacting protein 2 | -1.4 | 6.40E-02 |
| Adamts9 | a disintegrin-like and metallopeptidase (reprolysin type) with thrombospondin type 1 motif, 9 | -1.4 | 1.70E-02 |
| Kcnf1 | potassium voltage-gated channel, subfamily F, member 1 | -1.4 | 1.80E-02 |
| Grb7 | growth factor receptor bound protein 7 | -1.4 | 3.00E-02 |
| Sox9 | SRY (sex determining region Y)-box 9 | -1.4 | 2.80E-02 |
| Mis18bp1 | MIS18 binding protein 1 | -1.4 | 5.10E-02 |
| n-R5s204 | nuclear encoded rRNA 5S 204 | -1.4 | 3.00E-02 |
| Unc13b | unc-13 homolog B (C. elegans) | -1.4 | 3.70E-02 |
| LOC102634459 | uncharacterized LOC102634459 | -1.4 | 3.40E-02 |
| Ift46 | intraflagellar transport 46 | -1.4 | 7.10E-02 |
| Cdc25c | cell division cycle 25C | -1.4 | 4.40E-02 |
| Ubl5 | ubiquitin-like 5 | -1.4 | 2.80E-02 |
| Ano1 | anoctamin 1, calcium activated chloride channel | -1.4 | 7.50E-03 |
| Mir181c | microRNA 181c | -1.4 | 4.40E-02 |
| Slc44a2 | solute carrier family 44, member 2 | -1.4 | 3.00E-02 |
| Prdm16 | PR domain containing 16 | -1.4 | 5.10E-02 |
| Gnb5 | guanine nucleotide binding protein (G protein), beta 5 | -1.4 | 6.00E-02 |
| Zfp750 | zinc finger protein 750 | -1.4 | 2.70E-02 |
| Herc3 | hect domain and RLD 3 | -1.4 | 1.80E-02 |
| Mir5103 | microRNA 5103 | -1.4 | 6.80E-02 |
| Fut1 | fucosyltransferase 1 | -1.4 | 5.30E-02 |
| S1pr2 | sphingosine-1-phosphate receptor 2 | -1.4 | 2.40E-02 |
| Frat1 | frequently rearranged in advanced T cell lymphomas | -1.4 | 6.80E-02 |
| Ube2c | ubiquitin-conjugating enzyme E2C | -1.4 | 8.40E-02 |
| Col18a1 | collagen, type XVIII, alpha 1 | -1.4 | 5.70E-02 |
| Dennd5b | DENN/MADD domain containing 5B | -1.4 | 9.10E-02 |
| Scara3 | scavenger receptor class A, member 3 | -1.4 | 7.40E-02 |
| Rps26 | ribosomal protein S26 | -1.4 | 7.40E-02 |
| Rbm41 | RNA binding motif protein 41 | -1.4 | 3.40E-02 |
| St6gal1 | beta galactoside alpha 2,6 sialyltransferase 1 | -1.4 | 9.00E-02 |
| Pola1 | polymerase (DNA directed), alpha 1 | -1.4 | 1.70E-02 |
| Pigh | phosphatidylinositol glycan anchor biosynthesis, class H | -1.4 | 6.40E-02 |
| Xiap | X-linked inhibitor of apoptosis | -1.4 | 8.00E-02 |
| Reps2 | RALBP1 associated Eps domain containing protein 2 | -1.4 | 3.20E-02 |
| Bcl2l14 | BCL2-like 14 (apoptosis facilitator) | -1.4 | 6.20E-02 |
| Shroom4 | shroom family member 4 | -1.4 | 3.70E-02 |
| LOC102638005 | uncharacterized LOC102638005 | -1.4 | 2.80E-02 |
| Pdia6 | protein disulfide isomerase associated 6 | -1.4 | 3.30E-02 |
| Gyltl1b | glycosyltransferase-like 1B | -1.4 | 2.70E-02 |
| Plxnb1 | plexin B1 | -1.4 | 5.30E-02 |
| Mmgt2 | membrane magnesium transporter 2 | -1.4 | 5.50E-02 |
| Coa4 | cytochrome c oxidase assembly factor 4 | -1.4 | 5.40E-02 |
| Smox | spermine oxidase | -1.4 | 5.80E-02 |
| Lmnb1 | lamin B1 | -1.4 | 6.20E-02 |
| Brwd3 | bromodomain and WD repeat domain containing 3 | -1.4 | 9.10E-02 |
| Ncapd2 | non-SMC condensin I complex, subunit D2 | -1.4 | 5.80E-02 |
| Dos | downstream of Stk11 | -1.4 | 1.60E-02 |
| Tmed1 | transmembrane emp24 domain containing 1 | -1.4 | 6.80E-02 |
| Bcl2 | B cell leukemia/lymphoma 2 | -1.4 | 1.50E-02 |
| Abca5 | ATP-binding cassette, sub-family A (ABC1), member 5 | -1.4 | 8.30E-02 |
| Smpd2 | sphingomyelin phosphodiesterase 2, neutral | -1.4 | 5.80E-02 |
| LOC102636398 | uncharacterized LOC102636398 | -1.4 | 9.20E-02 |
| Hhex | hematopoietically expressed homeobox | -1.4 | 6.40E-02 |
| Tbc1d8 | TBC1 domain family, member 8 | -1.4 | 9.20E-02 |
| Ccnt2 | cyclin T2 | -1.4 | 3.60E-02 |
| Hdac6 | histone deacetylase 6 | -1.4 | 3.30E-02 |
| Entpd4 | ectonucleoside triphosphate diphosphohydrolase 4 | -1.4 | 6.30E-02 |
| Pomgnt2 | protein O-linked mannose beta 1,4-N-acetylglucosaminyltransferase 2 | -1.4 | 1.30E-02 |
| Wdr20 | WD repeat domain 20 | -1.4 | 7.30E-02 |
| Ccdc149 | coiled-coil domain containing 149 | -1.4 | 7.90E-02 |
| Poli | polymerase (DNA directed), iota | -1.4 | 4.20E-02 |
| Mrpl4 | mitochondrial ribosomal protein L4 | -1.4 | 4.30E-02 |
| Smtnl2 | smoothelin-like 2 | -1.4 | 3.70E-02 |
| Cox16 | cytochrome c oxidase assembly protein 16 | -1.4 | 7.10E-02 |
| Shroom2 | shroom family member 2 | -1.4 | 1.40E-02 |
| Zdhhc9 | zinc finger, DHHC domain containing 9 | -1.4 | 3.10E-02 |
| Tarsl2 | threonyl-tRNA synthetase-like 2 | -1.4 | 7.30E-02 |
| Il34 | interleukin 34 | -1.4 | 6.00E-03 |
| Sike1 | suppressor of IKBKE 1 | -1.4 | 5.50E-02 |
| Pvrl4 | poliovirus receptor-related 4 | -1.4 | 7.40E-02 |
| Carm1 | coactivator-associated arginine methyltransferase 1 | -1.4 | 3.30E-02 |
| Tmem91 | transmembrane protein 91 | -1.4 | 2.10E-02 |
| Syn3 | synapsin III | -1.4 | 4.00E-02 |
| Edn3 | endothelin 3 | -1.4 | 6.90E-02 |
| Sh3rf2 | SH3 domain containing ring finger 2 | -1.4 | 2.90E-02 |
| Alms1 | Alstrom syndrome 1 | -1.4 | 1.40E-02 |
| AI480526 | expressed sequence AI480526 | -1.4 | 2.20E-02 |
| Igsf9b | immunoglobulin superfamily, member 9B | -1.4 | 8.90E-02 |
| Fam76b | family with sequence similarity 76, member B | -1.4 | 8.30E-02 |
| Il23a | interleukin 23, alpha subunit p19 | -1.4 | 3.40E-02 |
| Mir186 | microRNA 186 | -1.4 | 6.00E-02 |
| Atp6v1g2 | ATPase, H+ transporting, lysosomal V1 subunit G2 | -1.4 | 3.70E-02 |
| Cln6 | ceroid-lipofuscinosis, neuronal 6 | -1.4 | 1.40E-02 |
| Olfm4 | olfactomedin 4 | -1.4 | 2.10E-02 |
| Snora28 | small nucleolar RNA, H/ACA box 28 | -1.4 | 2.80E-02 |
| Ralgapa1 | Ral GTPase activating protein, alpha subunit 1 | -1.4 | 4.30E-02 |
| BC030867 | cDNA sequence BC030867 | -1.4 | 2.60E-02 |
| Dapp1 | dual adaptor for phosphotyrosine and 3-phosphoinositides 1 | -1.4 | 4.60E-02 |
| Trbj2-6 | T cell receptor beta joining 2-6 | -1.4 | 7.10E-02 |
| Knstrn | kinetochore-localized astrin/SPAG5 binding | -1.4 | 8.10E-02 |
| LOC102636154 | uncharacterized LOC102636154 | -1.4 | 5.80E-02 |
| Gsap | gamma-secretase activating protein | -1.4 | 6.80E-02 |
| Scara5 | scavenger receptor class A, member 5 (putative) | -1.4 | 5.20E-02 |
| Krtap17-1 | keratin associated protein 17-1 | -1.4 | 5.80E-02 |
| Eef2k | eukaryotic elongation factor-2 kinase | -1.4 | 9.70E-02 |
| Vsig10 | V-set and immunoglobulin domain containing 10 | -1.4 | 5.10E-02 |
| Slc25a23 | solute carrier family 25 (mitochondrial carrier; phosphate carrier), member 23 | -1.4 | 9.40E-02 |
| Irak1 | interleukin-1 receptor-associated kinase 1 | -1.4 | 6.60E-02 |
| Armc10 | armadillo repeat containing 10 | -1.4 | 4.60E-02 |
| Itgb1bp2 | integrin beta 1 binding protein 2 | -1.4 | 2.90E-02 |
| Xk | Kell blood group precursor (McLeod phenotype) homolog | -1.4 | 2.30E-02 |
| Reps1 | RalBP1 associated Eps domain containing protein | -1.4 | 6.30E-02 |
| Suv39h1 | suppressor of variegation 3-9 homolog 1 (Drosophila) | -1.4 | 8.60E-02 |
| Lrfn3 | leucine rich repeat and fibronectin type III domain containing 3 | -1.4 | 9.20E-02 |
| Dcp1b | DCP1 decapping enzyme homolog B (S. cerevisiae) | -1.4 | 1.90E-02 |
| Mir32 | microRNA 32 | -1.4 | 4.40E-02 |
| Foxp1 | forkhead box P1 | -1.4 | 3.90E-02 |
| Fancm | Fanconi anemia, complementation group M | -1.4 | 2.50E-02 |
| Efcab12 | EF-hand calcium binding domain 12 | -1.4 | 6.40E-02 |
| Ccdc88c | coiled-coil domain containing 88C | -1.4 | 4.30E-02 |
| Tmem86b | transmembrane protein 86B | -1.4 | 5.40E-02 |
| Plekhh2 | pleckstrin homology domain containing, family H (with MyTH4 domain) member 2 | -1.4 | 3.20E-02 |
| Thsd4 | thrombospondin, type I, domain containing 4 | -1.4 | 3.80E-02 |
| Ncmap | noncompact myelin associated protein | -1.4 | 7.90E-02 |
| B3gnt7 | UDP-GlcNAc:betaGal beta-1,3-N-acetylglucosaminyltransferase 7 | -1.4 | 8.00E-02 |
| Map7 | microtubule-associated protein 7 | -1.4 | 7.10E-02 |
| Btc | betacellulin, epidermal growth factor family member | -1.4 | 5.40E-02 |
| LOC102641980 | uncharacterized LOC102641980 | -1.4 | 1.30E-02 |
| Igsf3 | immunoglobulin superfamily, member 3 | -1.4 | 8.10E-02 |
| Rnf43 | ring finger protein 43 | -1.4 | 5.40E-02 |
| Ccdc36 | coiled-coil domain containing 36 | -1.4 | 7.50E-02 |
| Pxylp1 | 2-phosphoxylose phosphatase 1 | -1.3 | 2.60E-02 |
| Ctsh | cathepsin H | -1.3 | 6.40E-02 |
| Araf | v-raf murine sarcoma 3611 viral oncogene homolog | -1.3 | 8.20E-03 |
| Caprin2 | caprin family member 2 | -1.3 | 1.70E-02 |
| Cdcp1 | CUB domain containing protein 1 | -1.3 | 8.90E-02 |
| Rnf144a | ring finger protein 144A | -1.3 | 2.00E-02 |
| Nfrkb | nuclear factor related to kappa B binding protein | -1.3 | 5.30E-02 |
| Zfp398 | zinc finger protein 398 | -1.3 | 5.50E-02 |
| Zfp467 | zinc finger protein 467 | -1.3 | 7.20E-02 |
| Fgd1 | FYVE, RhoGEF and PH domain containing 1 | -1.3 | 3.00E-02 |
| Nono | non-POU-domain-containing, octamer binding protein | -1.3 | 5.30E-02 |
| Tspan1 | tetraspanin 1 | -1.3 | 4.50E-02 |
| L2hgdh | L-2-hydroxyglutarate dehydrogenase | -1.3 | 3.70E-02 |
| Fam171a2 | family with sequence similarity 171, member A2 | -1.3 | 2.20E-02 |
| Taf1b | TATA box binding protein (Tbp)-associated factor, RNA polymerase I, B | -1.3 | 6.60E-02 |
| Snx14 | sorting nexin 14 | -1.3 | 3.00E-02 |
| Tssk4 | testis-specific serine kinase 4 | -1.3 | 9.30E-02 |
| Zan | zonadhesin | -1.3 | 8.30E-02 |
| Mbip | MAP3K12 binding inhibitory protein 1 | -1.3 | 9.90E-02 |
| Rgl3 | ral guanine nucleotide dissociation stimulator-like 3 | -1.3 | 6.00E-02 |
| Rad54l2 | RAD54 like 2 (S. cerevisiae) | -1.3 | 5.00E-02 |
| Elovl5 | ELOVL family member 5, elongation of long chain fatty acids (yeast) | -1.3 | 9.80E-02 |
| Mob3a | MOB kinase activator 3A | -1.3 | 4.20E-02 |
| Spata24 | spermatogenesis associated 24 | -1.3 | 6.60E-02 |
| Ip6k2 | inositol hexaphosphate kinase 2 | -1.3 | 3.40E-02 |
| Phf8 | PHD finger protein 8 | -1.3 | 2.70E-02 |
| Kif20b | kinesin family member 20B | -1.3 | 4.40E-02 |
| Dnah11 | dynein, axonemal, heavy chain 11 | -1.3 | 5.30E-02 |
| Tle2 | transducin-like enhancer of split 2, homolog of Drosophila E(spl) | -1.3 | 3.10E-02 |
| Arhgap39 | Rho GTPase activating protein 39 | -1.3 | 7.30E-02 |
| Plet1 | placenta expressed transcript 1 | -1.3 | 5.10E-02 |
| Scg5 | secretogranin V | -1.3 | 4.80E-02 |
| Dennd3 | DENN/MADD domain containing 3 | -1.3 | 2.50E-02 |
| Cpsf6 | cleavage and polyadenylation specific factor 6 | -1.3 | 7.70E-02 |
| Evpl | envoplakin | -1.3 | 2.90E-02 |
| Sestd1 | SEC14 and spectrin domains 1 | -1.3 | 6.60E-02 |
| Fam60a | family with sequence similarity 60, member A | -1.3 | 6.00E-02 |
| Ap1s3 | adaptor-related protein complex AP-1, sigma 3 | -1.3 | 4.00E-02 |
| Rpl10a-ps2 | ribosomal protein L10A, pseudogene 2 | -1.3 | 6.80E-02 |
| Chm | choroidermia | -1.3 | 5.00E-02 |
| Eif4ebp2 | eukaryotic translation initiation factor 4E binding protein 2 | -1.3 | 3.30E-02 |
| Lipg | lipase, endothelial | -1.3 | 9.90E-02 |
| Zfp251 | zinc finger protein 251 | -1.3 | 6.50E-02 |
| Zmym3 | zinc finger, MYM-type 3 | -1.3 | 6.10E-02 |
| Gpr126 | G protein-coupled receptor 126 | -1.3 | 8.30E-02 |
| Zswim5 | zinc finger SWIM-type containing 5 | -1.3 | 3.60E-02 |
| Faah | fatty acid amide hydrolase | -1.3 | 6.90E-02 |
| Pcyox1 | prenylcysteine oxidase 1 | -1.3 | 8.00E-02 |
| BC023719 | cDNA sequence BC023719 | -1.3 | 3.20E-02 |
| Exoc3l | exocyst complex component 3-like | -1.3 | 4.30E-02 |
| Bace1 | beta-site APP cleaving enzyme 1 | -1.3 | 5.50E-02 |
| Zfp26 | zinc finger protein 26 | -1.3 | 9.00E-02 |
| Crip2 | cysteine rich protein 2 | -1.3 | 4.10E-02 |
| Rpgrip1 | retinitis pigmentosa GTPase regulator interacting protein 1 | -1.3 | 9.00E-02 |
| Nfatc2 | nuclear factor of activated T cells, cytoplasmic, calcineurin dependent 2 | -1.3 | 2.30E-02 |
| Qprt | quinolinate phosphoribosyltransferase | -1.3 | 3.80E-02 |
| Acnat1 | acyl-coenzyme A amino acid N-acyltransferase 1 | -1.3 | 4.80E-02 |
| Mdm1 | transformed mouse 3T3 cell double minute 1 | -1.3 | 2.50E-02 |
| Diap3 | diaphanous homolog 3 (Drosophila) | -1.3 | 7.60E-02 |
| Samd10 | sterile alpha motif domain containing 10 | -1.3 | 8.90E-02 |
| Maged1 | melanoma antigen, family D, 1 | -1.3 | 5.10E-02 |
| Zfp839 | zinc finger protein 839 | -1.3 | 4.80E-02 |
| Slc26a2 | solute carrier family 26 (sulfate transporter), member 2 | -1.3 | 9.90E-02 |
| Acta2 | actin, alpha 2, smooth muscle, aorta | -1.3 | 4.20E-02 |
| Gen1 | Gen homolog 1, endonuclease (Drosophila) | -1.3 | 7.20E-02 |
| Tmem194b | transmembrane protein 194B | -1.3 | 6.70E-02 |
| Coro2b | coronin, actin binding protein, 2B | -1.3 | 3.60E-02 |
| Tsga10 | testis specific 10 | -1.3 | 5.10E-02 |
| Wdr45 | WD repeat domain 45 | -1.3 | 5.30E-02 |
| Raver1-fdx1l | Raver1-Fdx1l readthrough | -1.3 | 6.60E-02 |
| Lurap1 | leucine rich adaptor protein 1 | -1.3 | 8.00E-02 |
| Gpr142 | G protein-coupled receptor 142 | -1.3 | 3.80E-02 |
| Hs3st3a1 | heparan sulfate (glucosamine) 3-O-sulfotransferase 3A1 | -1.3 | 3.60E-02 |
| Zfp599 | zinc finger protein 599 | -1.3 | 7.30E-02 |
| Timp3 | tissue inhibitor of metalloproteinase 3 | -1.3 | 3.00E-02 |
| Rps4x | ribosomal protein S4, X-linked | -1.3 | 6.90E-02 |
| Pard6a | par-6 family cell polarity regulator alpha | -1.3 | 8.30E-02 |
| Tmem87b | transmembrane protein 87B | -1.3 | 9.50E-02 |
| Crtc1 | CREB regulated transcription coactivator 1 | -1.3 | 7.20E-02 |
| Ccdc15 | coiled-coil domain containing 15 | -1.3 | 7.20E-02 |
| Sigirr | single immunoglobulin and toll-interleukin 1 receptor (TIR) domain | -1.3 | 9.10E-02 |
| Hmg20a | high mobility group 20A | -1.3 | 9.30E-02 |
| Slc37a3 | solute carrier family 37 (glycerol-3-phosphate transporter), member 3 | -1.3 | 9.90E-02 |
| Cecr5 | cat eye syndrome chromosome region, candidate 5 | -1.3 | 7.70E-02 |
| Zfp827 | zinc finger protein 827 | -1.3 | 5.30E-02 |
| Gpr173 | G-protein coupled receptor 173 | -1.3 | 7.50E-02 |
| Gpr56 | G protein-coupled receptor 56 | -1.3 | 8.70E-02 |
| Slc27a1 | solute carrier family 27 (fatty acid transporter), member 1 | -1.3 | 6.60E-02 |
| Wdr27 | WD repeat domain 27 | -1.3 | 5.30E-02 |
| Mar9 | membrane-associated ring finger (C3HC4) 9 | -1.3 | 3.70E-02 |
| Rfx7 | regulatory factor X, 7 | -1.3 | 9.40E-02 |
| Med14 | mediator complex subunit 14 | -1.3 | 7.30E-02 |
| Tmtc4 | transmembrane and tetratricopeptide repeat containing 4 | -1.3 | 8.70E-02 |
| Gcfc2 | GC-rich sequence DNA binding factor 2 | -1.3 | 7.40E-02 |
| Col4a6 | collagen, type IV, alpha 6 | -1.3 | 1.40E-02 |
| Magi1 | membrane associated guanylate kinase, WW and PDZ domain containing 1 | -1.3 | 4.40E-02 |
| Smarca4 | SWI/SNF related, matrix associated, actin dependent regulator of chromatin, subfamily a, member 4 | -1.3 | 9.90E-02 |
| Mfsd6 | major facilitator superfamily domain containing 6 | -1.3 | 9.00E-02 |
| Gata6 | GATA binding protein 6 | -1.3 | 9.80E-02 |
| Foxi3 | forkhead box I3 | -1.3 | 7.30E-02 |
| Tgfbr1 | transforming growth factor, beta receptor I | -1.3 | 4.10E-02 |
| Paox | polyamine oxidase (exo-N4-amino) | -1.3 | 2.70E-02 |
| Impdh2 | inosine 5-phosphate dehydrogenase 2 | -1.3 | 8.20E-02 |
| Popdc2 | popeye domain containing 2 | -1.3 | 6.40E-02 |
| Cort | cortistatin | -1.3 | 9.10E-02 |
| Thumpd2 | THUMP domain containing 2 | -1.3 | 9.50E-02 |
| AI463170 | expressed sequence AI463170 | -1.3 | 4.70E-02 |
| Epb4.2 | erythrocyte protein band 4.2 | -1.3 | 9.90E-02 |
| Foxa2 | forkhead box A2 | -1.3 | 4.40E-02 |
| Slco2a1 | solute carrier organic anion transporter family, member 2a1 | -1.3 | 4.40E-02 |
| Bbs9 | Bardet-Biedl syndrome 9 (human) | -1.3 | 9.60E-02 |
| Ftsj1 | FtsJ homolog 1 (E. coli) | -1.3 | 6.80E-02 |
| Hoga1 | 4-hydroxy-2-oxoglutarate aldolase 1 | -1.3 | 5.10E-02 |
| Ccdc120 | coiled-coil domain containing 120 | -1.3 | 4.20E-02 |
| Wwox | WW domain-containing oxidoreductase | -1.3 | 9.80E-02 |
| LOC101055802 | uncharacterized LOC101055802 | -1.3 | 4.80E-02 |
| Pcnxl4 | pecanex-like 4 (Drosophila) | -1.3 | 2.60E-02 |
| Ybx3 | Y box protein 3 | -1.3 | 9.30E-02 |
| Rpl12 | ribosomal protein L12 | -1.3 | 6.60E-02 |
| Mir763 | microRNA 763 | -1.3 | 8.60E-02 |
| Ccdc18 | coiled-coil domain containing 18 | -1.3 | 9.70E-02 |
| Ahr | aryl-hydrocarbon receptor | -1.3 | 6.30E-02 |
| Psapl1 | prosaposin-like 1 | -1.3 | 4.20E-02 |
| Bcl9l | B cell CLL/lymphoma 9-like | -1.3 | 6.20E-02 |
| Sep9 | septin 9 | -1.3 | 9.60E-02 |
| Amn1 | antagonist of mitotic exit network 1 | -1.3 | 6.70E-02 |
| Zfp612 | zinc finger protein 612 | -1.3 | 5.20E-02 |
| Whrn | whirlin | -1.3 | 4.10E-02 |
| Mast1 | microtubule associated serine/threonine kinase 1 | -1.3 | 4.30E-02 |
| Cep83os | centrosomal protein 83, opposite strand | -1.3 | 7.40E-02 |
| Prx | periaxin | -1.3 | 6.40E-02 |
| Cradd | CASP2 and RIPK1 domain containing adaptor with death domain | -1.3 | 9.20E-02 |
| Amotl1 | angiomotin-like 1 | -1.3 | 3.90E-02 |
| Rny3 | RNA, Y3 small cytoplasmic (associated with Ro protein) | -1.3 | 7.10E-02 |
| Myo1e | myosin IE | -1.3 | 6.10E-02 |
| Celf4 | CUGBP, Elav-like family member 4 | -1.3 | 6.80E-02 |
| Rpl17 | ribosomal protein L17 | -1.3 | 9.60E-02 |
| Eef1a1 | eukaryotic translation elongation factor 1 alpha 1 | -1.3 | 8.70E-02 |
| Cobl | cordon-bleu WH2 repeat | -1.3 | 9.70E-02 |
| Mospd4 | motile sperm domain containing 4 | -1.3 | 3.60E-02 |
| Tceal8 | transcription elongation factor A (SII)-like 8 | -1.3 | 3.20E-02 |
| Smad3 | SMAD family member 3 | -1.3 | 5.70E-02 |
| AW554918 | expressed sequence AW554918 | -1.3 | 6.70E-02 |
| Pacs2 | phosphofurin acidic cluster sorting protein 2 | -1.3 | 3.50E-02 |
| Nmnat3 | nicotinamide nucleotide adenylyltransferase 3 | -1.3 | 4.10E-02 |
| Efnb1 | ephrin B1 | -1.3 | 5.30E-02 |
| Ephb3 | Eph receptor B3 | -1.3 | 8.80E-02 |
| Cnn2 | calponin 2 | -1.3 | 9.10E-02 |
| Cenpf | centromere protein F | -1.3 | 7.20E-02 |
| Atad2b | ATPase family, AAA domain containing 2B | -1.3 | 8.00E-02 |
| LOC101055759 | uncharacterized LOC101055759 | -1.3 | 8.20E-02 |
| Phf14 | PHD finger protein 14 | -1.3 | 5.00E-02 |
| Ppil6 | peptidylprolyl isomerase (cyclophilin)-like 6 | -1.3 | 9.40E-02 |
| Slc31a2 | solute carrier family 31, member 2 | -1.3 | 8.90E-02 |
| Tusc2 | tumor suppressor candidate 2 | -1.3 | 6.00E-02 |
| Dmxl1 | Dmx-like 1 | -1.2 | 8.80E-02 |
| Amigo3 | adhesion molecule with Ig like domain 3 | -1.2 | 6.00E-02 |
| Vamp1 | vesicle-associated membrane protein 1 | -1.2 | 9.30E-02 |
| Saysd1 | SAYSVFN motif domain containing 1 | -1.2 | 5.60E-02 |
| Zfp362 | zinc finger protein 362 | -1.2 | 8.20E-02 |
| Hist1h2bb | histone cluster 1, H2bb | -1.2 | 9.60E-02 |
| LOC102634683 | uncharacterized LOC102634683 | -1.2 | 5.80E-02 |
| Arrdc1 | arrestin domain containing 1 | -1.2 | 4.10E-02 |
| Myo5b | myosin VB | -1.2 | 1.00E-01 |
| Exoc6b | exocyst complex component 6B | -1.2 | 7.30E-02 |
| Hnrnpf | heterogeneous nuclear ribonucleoprotein F | -1.2 | 5.40E-02 |
| Sepn1 | selenoprotein N, 1 | -1.2 | 7.60E-02 |
| Pde7a | phosphodiesterase 7A | -1.2 | 6.90E-02 |
| Rpl7a | ribosomal protein L7A | -1.2 | 7.60E-02 |
| Pcbp3 | poly(rC) binding protein 3 | -1.2 | 8.40E-02 |
| Tubg2 | tubulin, gamma 2 | -1.2 | 6.30E-02 |
| Lrrc8d | leucine rich repeat containing 8D | -1.2 | 9.70E-02 |
| Tmem14a | transmembrane protein 14A | -1.2 | 9.00E-02 |
| LOC102634941 | putative tubulin-like protein alpha-4B-like | -1.2 | 8.10E-02 |
| Ccdc91 | coiled-coil domain containing 91 | -1.2 | 7.80E-02 |
| Hbb-b2 | hemoglobin, beta adult minor chain | -1.2 | 5.80E-02 |
| Pex26 | peroxisomal biogenesis factor 26 | -1.2 | 5.00E-02 |
| Gsdmcl2 | gasdermin C-like 2 | -1.2 | 7.80E-02 |
| Plxna4os1 | plexin A4, opposite strand 1 | -1.2 | 4.80E-02 |
| Muc1 | mucin 1, transmembrane | -1.2 | 9.50E-02 |
| Gpr162 | G protein-coupled receptor 162 | -1.2 | 9.10E-02 |
| Zfp319 | zinc finger protein 319 | -1.2 | 6.20E-02 |
| Cbl | Casitas B-lineage lymphoma | -1.2 | 9.40E-02 |
| Zbtb32 | zinc finger and BTB domain containing 32 | -1.2 | 7.40E-02 |
| Apex2 | apurinic/apyrimidinic endonuclease 2 | -1.2 | 9.10E-02 |
| Zkscan7 | zinc finger with KRAB and SCAN domains 7 | -1.2 | 5.20E-02 |
| Nxnl2 | nucleoredoxin-like 2 | -1.2 | 8.40E-02 |
| Tgif2 | TGFB-induced factor homeobox 2 | -1.2 | 7.10E-02 |
| Epb4.1l5 | erythrocyte protein band 4.1-like 5 | -1.2 | 6.80E-02 |
| Atl1 | atlastin GTPase 1 | -1.2 | 6.70E-02 |
| Ccdc83 | coiled-coil domain containing 83 | -1.2 | 8.70E-02 |
| Msh5 | mutS homolog 5 (E. coli) | -1.2 | 5.80E-02 |
| Pigl | phosphatidylinositol glycan anchor biosynthesis, class L | -1.2 | 7.10E-02 |
| Blk | B lymphoid kinase | -1.2 | 9.20E-02 |
| Npdc1 | neural proliferation, differentiation and control 1 | -1.2 | 9.70E-02 |
| Amigo1 | adhesion molecule with Ig like domain 1 | -1.2 | 8.30E-02 |
| Ppm1n | protein phosphatase, Mg2+/Mn2+ dependent, 1N (putative) | -1.2 | 5.60E-02 |
| Fam168b | family with sequence similarity 168, member B | -1.2 | 8.80E-02 |
| LOC102635645 | uncharacterized LOC102635645 | -1.2 | 7.00E-02 |
| Casc1 | cancer susceptibility candidate 1 | -1.2 | 8.80E-02 |
| Zfr2 | zinc finger RNA binding protein 2 | -1.2 | 8.80E-02 |
| Eif4g3 | eukaryotic translation initiation factor 4 gamma, 3 | -1.2 | 9.10E-02 |
| Zfhx3 | zinc finger homeobox 3 | -1.2 | 7.80E-02 |
| Prss41 | protease, serine 41 | -1.2 | 9.20E-02 |
| Slc29a2 | solute carrier family 29 (nucleoside transporters), member 2 | -1.2 | 8.50E-02 |
| LOC102636650 | uncharacterized LOC102636650 | -1.2 | 6.30E-02 |
| Snai2 | snail family zinc finger 2 | -1.2 | 9.30E-02 |
| Zfp182 | zinc finger protein 182 | -1.2 | 9.20E-02 |
| Klf7 | Kruppel-like factor 7 (ubiquitous) | -1.2 | 6.70E-02 |
| Tdrp | testis development related protein | -1.2 | 5.80E-02 |
| Clcnkb | chloride channel Kb | -1.2 | 7.90E-02 |
| Plk4 | polo-like kinase 4 | -1.2 | 9.40E-02 |
| Adat2 | adenosine deaminase, tRNA-specific 2 | -1.2 | 9.10E-02 |
| Tmem206 | transmembrane protein 206 | -1.2 | 9.40E-02 |
| Tpk1 | thiamine pyrophosphokinase | -1.2 | 9.10E-02 |
| Fam131c | family with sequence similarity 131, member C | -1.2 | 8.30E-02 |
| Pik3ip1 | phosphoinositide-3-kinase interacting protein 1 | -1.2 | 8.40E-02 |

*Fc:* fold change, *OE:* overexpression.
