# Supplementary material for: Snail mediates repression of the Dlk1-Dio3 locus in lung tumor-infiltrating immune cells
Source: Oncotarget. 2018 Aug 17;9(64):32331–45. doi: 10.18632/oncotarget.25965 (PMC6122344; doi:10.18632/oncotarget.25965)
Supplement: Supplementary file 3 [file oncotarget-09-32331-s003.docx]

**Supplementary Table 2: List of 830 upregulated genes in Snail KD tumors from Figure 1A**

| **Symbol** | **Description** | **fc** | **p-value** |
| --- | --- | --- | --- |
| Ldoc1 | leucine zipper, down-regulated in cancer 1 | 1.1 | 9.00E-02 |
| Nudt13 | nudix (nucleoside diphosphate linked moiety X)-type motif 13 | 1.1 | 7.90E-02 |
| Capns1 | calpain, small subunit 1 | 1.1 | 8.20E-02 |
| Snora47 | small nucleolar RNA, H/ACA box 47 | 1.1 | 4.20E-02 |
| Klk9 | kallikrein related-peptidase 9 | 1.1 | 7.40E-02 |
| Tmem39a | transmembrane protein 39a | 1.1 | 9.70E-02 |
| Nup43 | nucleoporin 43 | 1.1 | 8.30E-02 |
| Hiat1 | hippocampus abundant gene transcript 1 | 1.1 | 1.00E-01 |
| Fabp3 | fatty acid binding protein 3, muscle and heart | 1.1 | 9.00E-02 |
| LOC102636355 | uncharacterized LOC102636355 | 1.1 | 7.10E-02 |
| Xrcc2 | X-ray repair complementing defective repair in Chinese hamster cells 2 | 1.1 | 9.30E-02 |
| Neurl2 | neuralized-like 2 (Drosophila) | 1.1 | 6.70E-02 |
| Lin7c | lin-7 homolog C (C. elegans) | 1.1 | 7.80E-02 |
| S100a7a | S100 calcium binding protein A7A | 1.1 | 8.60E-02 |
| Ssna1 | Sjogrens syndrome nuclear autoantigen 1 | 1.1 | 9.50E-02 |
| Polr3k | polymerase (RNA) III (DNA directed) polypeptide K | 1.1 | 6.00E-02 |
| Dnajc19-ps // Dnajc19 | DnaJ (Hsp40) homolog, subfamily C, member 19, pseudogene // DnaJ (Hsp40) homolog, subfamily C, member 19 | 1.1 | 5.80E-02 |
| Fam78b | family with sequence similarity 78, member B | 1.1 | 7.80E-02 |
| Blmh | bleomycin hydrolase | 1.1 | 9.20E-02 |
| Tk1 | thymidine kinase 1 | 1.1 | 8.10E-02 |
| Immp1l | IMP1 inner mitochondrial membrane peptidase-like (S. cerevisiae) | 1.1 | 9.80E-02 |
| Rgn | regucalcin | 1.1 | 8.30E-02 |
| Urb2 | URB2 ribosome biogenesis 2 homolog (S. cerevisiae) | 1.1 | 9.60E-02 |
| Mrps22 | mitochondrial ribosomal protein S22 | 1.1 | 8.10E-02 |
| Dnajc21 | DnaJ (Hsp40) homolog, subfamily C, member 21 | 1.1 | 9.80E-02 |
| Deb1 | differentially expressed in B16F10 1 | 1.1 | 9.50E-02 |
| Hhat | hedgehog acyltransferase | 1.2 | 8.70E-02 |
| Cep97 | centrosomal protein 97 | 1.2 | 7.40E-02 |
| Pop4 | processing of precursor 4, ribonuclease P/MRP family, (S. cerevisiae) | 1.2 | 9.30E-02 |
| Dedd | death effector domain-containing | 1.2 | 7.10E-02 |
| Psmd6 | proteasome (prosome, macropain) 26S subunit, non-ATPase, 6 | 1.2 | 7.00E-02 |
| Tram1 | translocating chain-associating membrane protein 1 | 1.2 | 4.50E-02 |
| Kifap3 | kinesin-associated protein 3 | 1.2 | 9.60E-02 |
| Cmss1 | cms small ribosomal subunit 1 | 1.2 | 3.40E-02 |
| Oxnad1 | oxidoreductase NAD-binding domain containing 1 | 1.2 | 7.20E-02 |
| Adamts8 | a disintegrin-like and metallopeptidase (reprolysin type) with thrombospondin type 1 motif, 8 | 1.2 | 7.80E-02 |
| Atp5g3 | ATP synthase, H+ transporting, mitochondrial F0 complex, subunit C3 (subunit 9) | 1.2 | 9.00E-02 |
| Mgat5b | mannoside acetylglucosaminyltransferase 5, isoenzyme B | 1.2 | 7.70E-02 |
| Lig4 | ligase IV, DNA, ATP-dependent | 1.2 | 3.50E-02 |
| Mtrf1 | mitochondrial translational release factor 1 | 1.2 | 8.60E-02 |
| Vwa7 | von Willebrand factor A domain containing 7 | 1.2 | 9.70E-02 |
| Asb13 | ankyrin repeat and SOCS box-containing 13 | 1.2 | 7.50E-02 |
| Mrps31 | mitochondrial ribosomal protein S31 | 1.2 | 8.10E-02 |
| Larp4 | La ribonucleoprotein domain family, member 4 | 1.2 | 5.30E-02 |
| Rwdd4a | RWD domain containing 4A | 1.2 | 7.30E-02 |
| Polr2i | polymerase (RNA) II (DNA directed) polypeptide I | 1.2 | 7.00E-02 |
| Nipa2 | non imprinted in Prader-Willi/Angelman syndrome 2 homolog (human) | 1.2 | 8.50E-02 |
| Oxa1l | oxidase assembly 1-like | 1.2 | 7.80E-02 |
| Orc6 | origin recognition complex, subunit 6 | 1.2 | 8.70E-02 |
| Mtif3 | mitochondrial translational initiation factor 3 | 1.2 | 7.60E-02 |
| Arl13b | ADP-ribosylation factor-like 13B | 1.2 | 7.30E-02 |
| Pcna | proliferating cell nuclear antigen | 1.2 | 3.90E-02 |
| Stac3 | SH3 and cysteine rich domain 3 | 1.2 | 9.00E-02 |
| Uqcrc2 | ubiquinol cytochrome c reductase core protein 2 | 1.2 | 4.60E-02 |
| Nckap1 | NCK-associated protein 1 | 1.2 | 5.00E-02 |
| Harbi1 | harbinger transposase derived 1 | 1.2 | 9.10E-02 |
| Tusc3 | tumor suppressor candidate 3 | 1.2 | 8.40E-02 |
| Ptrh2 | peptidyl-tRNA hydrolase 2 | 1.2 | 8.40E-02 |
| Sri | sorcin | 1.2 | 9.80E-02 |
| Pgam5 | phosphoglycerate mutase family member 5 | 1.2 | 6.00E-02 |
| Hhipl1 | hedgehog interacting protein-like 1 | 1.2 | 5.10E-02 |
| Dnajc24 | DnaJ (Hsp40) homolog, subfamily C, member 24 | 1.2 | 9.80E-02 |
| Dpcd | deleted in primary ciliary dyskinesia | 1.2 | 1.00E-01 |
| Tyms | thymidylate synthase | 1.2 | 8.60E-02 |
| Pddc1 | Parkinson disease 7 domain containing 1 | 1.2 | 6.60E-02 |
| Fem1c | fem-1 homolog c (C.elegans) | 1.2 | 7.60E-02 |
| Fis1 | fission 1 (mitochondrial outer membrane) homolog (yeast) | 1.2 | 9.00E-02 |
| LOC102638888 | uncharacterized LOC102638888 | 1.2 | 6.50E-02 |
| Bahcc1 | BAH domain and coiled-coil containing 1 | 1.2 | 7.20E-02 |
| Actg1 | actin, gamma, cytoplasmic 1 | 1.2 | 6.90E-02 |
| Zfp385b | zinc finger protein 385B | 1.2 | 8.40E-02 |
| Cdc7 | cell division cycle 7 (S. cerevisiae) | 1.2 | 4.00E-02 |
| Rtn3 | reticulon 3 | 1.2 | 8.50E-02 |
| Setmar | SET domain without mariner transposase fusion | 1.2 | 9.40E-02 |
| Pacrgl | PARK2 co-regulated-like | 1.2 | 6.40E-02 |
| LOC102637788 | uncharacterized LOC102637788 | 1.2 | 7.20E-02 |
| Sepp1 | selenoprotein P, plasma, 1 | 1.2 | 6.90E-02 |
| Dusp19 | dual specificity phosphatase 19 | 1.2 | 7.20E-02 |
| Pnpt1 | polyribonucleotide nucleotidyltransferase 1 | 1.2 | 7.00E-02 |
| Gnpda2 | glucosamine-6-phosphate deaminase 2 | 1.2 | 8.30E-02 |
| Zcchc10 | zinc finger, CCHC domain containing 10 | 1.2 | 9.90E-02 |
| Atpaf1 | ATP synthase mitochondrial F1 complex assembly factor 1 | 1.2 | 6.20E-02 |
| Npm3 | nucleoplasmin 3 | 1.2 | 6.50E-02 |
| Drc1 | dynein regulatory complex subunit 1 | 1.2 | 9.10E-02 |
| Apex1 | apurinic/apyrimidinic endonuclease 1 | 1.2 | 9.10E-02 |
| LOC102632739 | dolichyl-diphosphooligosaccharide--protein glycosyltransferase subunit 4-like | 1.2 | 4.70E-02 |
| Ndufc2 | NADH dehydrogenase (ubiquinone) 1, subcomplex unknown, 2 | 1.2 | 8.40E-02 |
| Farsb | phenylalanyl-tRNA synthetase, beta subunit | 1.2 | 3.90E-02 |
| Sqrdl | sulfide quinone reductase-like (yeast) | 1.2 | 7.00E-02 |
| Timmdc1 | translocase of inner mitochondrial membrane domain containing 1 | 1.2 | 3.10E-02 |
| Psmd12 | proteasome (prosome, macropain) 26S subunit, non-ATPase, 12 | 1.2 | 3.10E-02 |
| Tpp2 | tripeptidyl peptidase II | 1.2 | 5.70E-02 |
| Nit1 | nitrilase 1 | 1.2 | 5.30E-02 |
| Wdfy1 | WD repeat and FYVE domain containing 1 | 1.2 | 9.50E-02 |
| Pask | PAS domain containing serine/threonine kinase | 1.2 | 5.30E-02 |
| Sp3os | trans-acting transcription factor 3, opposite strand | 1.2 | 4.20E-02 |
| Peli1 | pellino 1 | 1.2 | 9.70E-02 |
| Abhd14a | abhydrolase domain containing 14A | 1.2 | 6.20E-02 |
| Sap18 | Sin3-associated polypeptide 18 | 1.2 | 5.30E-02 |
| Mapk1 | mitogen-activated protein kinase 1 | 1.2 | 4.60E-02 |
| Sertad1 | SERTA domain containing 1 | 1.2 | 8.00E-02 |
| Kctd9 | potassium channel tetramerisation domain containing 9 | 1.2 | 9.10E-02 |
| Gemin4 | gem (nuclear organelle) associated protein 4 | 1.2 | 2.80E-02 |
| Gtl3 | gene trap locus 3 | 1.2 | 3.40E-02 |
| Pcdh1 | protocadherin 1 | 1.2 | 9.50E-02 |
| Fbxo45 | F-box protein 45 | 1.2 | 7.00E-02 |
| Ssr3 | signal sequence receptor, gamma | 1.2 | 7.20E-02 |
| Banf1 | barrier to autointegration factor 1 | 1.2 | 3.20E-02 |
| Atp5j | ATP synthase, H+ transporting, mitochondrial F0 complex, subunit F | 1.2 | 5.20E-02 |
| Ccne1 | cyclin E1 | 1.2 | 7.70E-02 |
| Nr1h3 | nuclear receptor subfamily 1, group H, member 3 | 1.2 | 3.50E-02 |
| Erich1 | glutamate rich 1 | 1.2 | 4.80E-02 |
| Senp7 | SUMO1/sentrin specific peptidase 7 | 1.2 | 8.10E-02 |
| Tmx2 | thioredoxin-related transmembrane protein 2 | 1.2 | 2.90E-02 |
| Dars2 | aspartyl-tRNA synthetase 2 (mitochondrial) | 1.2 | 7.00E-02 |
| Wdr75 | WD repeat domain 75 | 1.2 | 6.50E-02 |
| Rnf219 | ring finger protein 219 | 1.2 | 8.90E-02 |
| Slc25a4 | solute carrier family 25 (mitochondrial carrier, adenine nucleotide translocator), member 4 | 1.2 | 9.60E-02 |
| Ube3c | ubiquitin protein ligase E3C | 1.2 | 7.30E-02 |
| Cdadc1 | cytidine and dCMP deaminase domain containing 1 | 1.2 | 9.90E-02 |
| LOC102642233 | 60S ribosomal protein L29-like | 1.2 | 3.00E-02 |
| Pds5a | PDS5, regulator of cohesion maintenance, homolog A (S. cerevisiae) | 1.2 | 9.20E-02 |
| Zfp937 | zinc finger protein 937 | 1.2 | 7.20E-02 |
| Tmem107 | transmembrane protein 107 | 1.2 | 9.30E-02 |
| Tmem138 | transmembrane protein 138 | 1.2 | 9.30E-02 |
| n-R5s160 | nuclear encoded rRNA 5S 160 | 1.2 | 8.70E-02 |
| Tada1 | transcriptional adaptor 1 | 1.2 | 1.00E-01 |
| Rpl10a | ribosomal protein L10A | 1.2 | 3.30E-02 |
| Wibg | within bgcn homolog (Drosophila) | 1.2 | 5.80E-02 |
| Mkln1os | muskelin 1, intracellular mediator containing kelch motifs, opposite strand | 1.2 | 2.00E-02 |
| Mtx2 | metaxin 2 | 1.2 | 4.80E-02 |
| Dcaf11 | DDB1 and CUL4 associated factor 11 | 1.2 | 5.70E-02 |
| Tab2 | TGF-beta activated kinase 1/MAP3K7 binding protein 2 | 1.2 | 4.90E-02 |
| Sft2d2 | SFT2 domain containing 2 | 1.2 | 6.70E-02 |
| Fbxo30 | F-box protein 30 | 1.2 | 5.40E-02 |
| Parp16 | poly (ADP-ribose) polymerase family, member 16 | 1.2 | 5.80E-02 |
| Hist1h4m | histone cluster 1, H4m | 1.2 | 1.40E-02 |
| Cep162 | centrosomal protein 162 | 1.2 | 9.70E-02 |
| Get4 | golgi to ER traffic protein 4 homolog (S. cerevisiae) | 1.2 | 9.70E-02 |
| Commd3 | COMM domain containing 3 | 1.2 | 7.40E-02 |
| Htatsf1 | HIV TAT specific factor 1 | 1.2 | 6.30E-02 |
| Dnajc28 | DnaJ (Hsp40) homolog, subfamily C, member 28 | 1.2 | 6.00E-02 |
| Dyrk1a | dual-specificity tyrosine-(Y)-phosphorylation regulated kinase 1a | 1.2 | 9.30E-02 |
| Siah1a | seven in absentia 1A | 1.2 | 5.00E-02 |
| Rab4b | RAB4B, member RAS oncogene family | 1.2 | 3.90E-02 |
| Snora43 | small nucleolar RNA, H/ACA box 43 | 1.2 | 2.40E-02 |
| Ankrd32 | ankyrin repeat domain 32 | 1.2 | 9.70E-02 |
| Slc10a7 | solute carrier family 10 (sodium/bile acid cotransporter family), member 7 | 1.2 | 3.90E-02 |
| Imp4 | IMP4, U3 small nucleolar ribonucleoprotein, homolog (yeast) | 1.2 | 5.00E-02 |
| Capn7 | calpain 7 | 1.2 | 4.40E-02 |
| Twf1 | twinfilin, actin-binding protein, homolog 1 (Drosophila) | 1.2 | 6.80E-02 |
| Mrpl39 | mitochondrial ribosomal protein L39 | 1.2 | 2.00E-02 |
| Armc8 | armadillo repeat containing 8 | 1.2 | 7.10E-02 |
| Ntf5 | neurotrophin 5 | 1.2 | 8.80E-02 |
| Trove2 | TROVE domain family, member 2 | 1.2 | 8.70E-02 |
| Zfp28 | zinc finger protein 28 | 1.2 | 6.00E-02 |
| Asgr1 | asialoglycoprotein receptor 1 | 1.2 | 6.90E-02 |
| Zw10 | zw10 kinetochore protein | 1.2 | 8.90E-02 |
| Pigg | phosphatidylinositol glycan anchor biosynthesis, class G | 1.2 | 6.80E-02 |
| Wdr5b | WD repeat domain 5B | 1.2 | 6.50E-02 |
| Lins | lines homolog (Drosophila) | 1.2 | 3.60E-02 |
| Rbm34 | RNA binding motif protein 34 | 1.2 | 8.70E-02 |
| Cops8 | COP9 (constitutive photomorphogenic) homolog, subunit 8 (Arabidopsis thaliana) | 1.2 | 6.40E-02 |
| Ust | uronyl-2-sulfotransferase | 1.2 | 5.70E-02 |
| Defb21 | defensin beta 21 | 1.2 | 4.50E-02 |
| Sirt3 | sirtuin 3 | 1.2 | 2.90E-02 |
| Cdk4 | cyclin-dependent kinase 4 | 1.2 | 8.20E-02 |
| Umps | uridine monophosphate synthetase | 1.2 | 4.80E-02 |
| Idh3g | isocitrate dehydrogenase 3 (NAD+), gamma | 1.2 | 7.40E-02 |
| Exoc5 | exocyst complex component 5 | 1.2 | 7.10E-02 |
| Ube2g1 | ubiquitin-conjugating enzyme E2G 1 | 1.2 | 5.50E-02 |
| Ech1 | enoyl coenzyme A hydratase 1, peroxisomal | 1.2 | 8.00E-02 |
| Lypla1 | lysophospholipase 1 | 1.2 | 6.60E-02 |
| Eri2 | exoribonuclease 2 | 1.2 | 4.30E-02 |
| Rnf6 | ring finger protein (C3H2C3 type) 6 | 1.2 | 6.90E-02 |
| Vdac1 | voltage-dependent anion channel 1 | 1.2 | 2.60E-02 |
| Orc5 | origin recognition complex, subunit 5 | 1.2 | 2.80E-02 |
| Utp20 | UTP20, small subunit (SSU) processome component, homolog (yeast) | 1.2 | 8.50E-02 |
| Rpp40 | ribonuclease P 40 subunit | 1.2 | 6.30E-02 |
| Wbp4 | WW domain binding protein 4 | 1.2 | 8.50E-02 |
| Nfe2l2 | nuclear factor, erythroid derived 2, like 2 | 1.2 | 9.90E-03 |
| Abhd10 | abhydrolase domain containing 10 | 1.2 | 8.90E-02 |
| Tmem55a | transmembrane protein 55A | 1.2 | 6.90E-02 |
| Anapc5 | anaphase-promoting complex subunit 5 | 1.2 | 9.70E-02 |
| Xpo7 | exportin 7 | 1.2 | 6.30E-02 |
| Klc2 | kinesin light chain 2 | 1.2 | 9.60E-02 |
| Ap1s1 | adaptor protein complex AP-1, sigma 1 | 1.2 | 6.90E-02 |
| Olfr374 | olfactory receptor 374 | 1.2 | 1.80E-02 |
| Mrpl34 | mitochondrial ribosomal protein L34 | 1.2 | 5.60E-02 |
| Nudt7 | nudix (nucleoside diphosphate linked moiety X)-type motif 7 | 1.2 | 6.10E-02 |
| Dbf4 | DBF4 homolog (S. cerevisiae) | 1.2 | 7.60E-02 |
| Nme1 | NME/NM23 nucleoside diphosphate kinase 1 | 1.2 | 4.60E-02 |
| Atp13a3 | ATPase type 13A3 | 1.2 | 7.40E-02 |
| Cops2 | COP9 (constitutive photomorphogenic) homolog, subunit 2 (Arabidopsis thaliana) | 1.2 | 3.50E-02 |
| Atp6v1h | ATPase, H+ transporting, lysosomal V1 subunit H | 1.2 | 4.60E-02 |
| Mdh2 | malate dehydrogenase 2, NAD (mitochondrial) | 1.2 | 3.10E-02 |
| Ppdpf | pancreatic progenitor cell differentiation and proliferation factor homolog (zebrafish)RIKEN cDNA 2700038C09 gene | 1.2 | 7.40E-02 |
| Vps37c | vacuolar protein sorting 37C (yeast) | 1.2 | 6.30E-02 |
| Prpsap2 | phosphoribosyl pyrophosphate synthetase-associated protein 2 | 1.2 | 9.40E-02 |
| Gpx1 | glutathione peroxidase 1 | 1.2 | 8.50E-02 |
| Eif3e | eukaryotic translation initiation factor 3, subunit E | 1.2 | 5.40E-02 |
| Tra2b | transformer 2 beta homolog (Drosophila) | 1.2 | 5.60E-02 |
| Zfp148 | zinc finger protein 148 | 1.2 | 4.30E-02 |
| Ube2w | ubiquitin-conjugating enzyme E2W (putative) | 1.2 | 5.80E-02 |
| Ufd1l | ubiquitin fusion degradation 1 like | 1.2 | 4.10E-02 |
| Abi2 | abl-interactor 2 | 1.2 | 7.10E-02 |
| Tmem216 | transmembrane protein 216 | 1.2 | 9.10E-02 |
| Dbi | diazepam binding inhibitor | 1.2 | 2.50E-02 |
| Rnls | renalase, FAD-dependent amine oxidase | 1.2 | 5.90E-02 |
| Tcta | T cell leukemia translocation altered gene | 1.2 | 5.70E-02 |
| Dctpp1 | dCTP pyrophosphatase 1 | 1.2 | 8.70E-02 |
| Morc1 | microrchidia 1 | 1.2 | 4.60E-02 |
| Sbf2 | SET binding factor 2 | 1.2 | 6.00E-02 |
| Zbtb11 | zinc finger and BTB domain containing 11 | 1.2 | 8.50E-02 |
| Zswim1 | zinc finger SWIM-type containing 1 | 1.2 | 6.40E-02 |
| Mpi | mannose phosphate isomerase | 1.2 | 8.70E-02 |
| Lrrc40 | leucine rich repeat containing 40 | 1.2 | 9.50E-02 |
| Abhd11 | abhydrolase domain containing 11 | 1.2 | 2.80E-02 |
| Wdr12 | WD repeat domain 12 | 1.2 | 5.80E-03 |
| LOC102633065 | uncharacterized LOC102633065 | 1.2 | 8.00E-02 |
| Eri1 | exoribonuclease 1 | 1.2 | 5.50E-02 |
| Mtm1 | X-linked myotubular myopathy gene 1 | 1.2 | 2.90E-02 |
| Cdip1 | cell death inducing Trp53 target 1 | 1.2 | 1.00E-01 |
| Agps | alkylglycerone phosphate synthase | 1.2 | 8.70E-02 |
| Chchd5 | coiled-coil-helix-coiled-coil-helix domain containing 5 | 1.2 | 3.90E-02 |
| Gnb2l1 | guanine nucleotide binding protein (G protein), beta polypeptide 2 like 1 | 1.2 | 6.00E-02 |
| Zfp329 | zinc finger protein 329 | 1.2 | 5.60E-02 |
| Mrpl16 | mitochondrial ribosomal protein L16 | 1.2 | 9.60E-02 |
| Mrpl57 | mitochondrial ribosomal protein L53 | 1.2 | 3.70E-02 |
| Mllt11 | myeloid/lymphoid or mixed-lineage leukemia (trithorax homolog, Drosophila); translocated to, 11 | 1.2 | 5.60E-02 |
| Rpl21-ps13 | ribosomal protein L21-ps13 | 1.2 | 6.80E-02 |
| Det1 | de-etiolated homolog 1 (Arabidopsis) | 1.2 | 1.70E-02 |
| Mir30d | microRNA 30d | 1.2 | 5.80E-02 |
| Cops5 | COP9 (constitutive photomorphogenic) homolog, subunit 5 (Arabidopsis thaliana) | 1.2 | 5.40E-02 |
| Gtf2e1 | general transcription factor II E, polypeptide 1 (alpha subunit) | 1.2 | 6.20E-02 |
| Heatr2 | HEAT repeat containing 2 | 1.2 | 6.90E-02 |
| Ube2d1 | ubiquitin-conjugating enzyme E2D 1 | 1.2 | 9.40E-02 |
| Krr1 | KRR1, small subunit (SSU) processome component, homolog (yeast) | 1.2 | 7.70E-02 |
| Orc2 | origin recognition complex, subunit 2 | 1.2 | 6.00E-02 |
| Idh2 | isocitrate dehydrogenase 2 (NADP+), mitochondrial | 1.2 | 9.30E-02 |
| Ccz1 | CCZ1 vacuolar protein trafficking and biogenesis associated | 1.2 | 4.60E-02 |
| Pcnp | PEST proteolytic signal containing nuclear protein | 1.2 | 5.60E-02 |
| Pisd | phosphatidylserine decarboxylase | 1.2 | 2.00E-02 |
| Rchy1 | ring finger and CHY zinc finger domain containing 1 | 1.2 | 9.40E-02 |
| Haus4 | HAUS augmin-like complex, subunit 4 | 1.2 | 4.50E-02 |
| Thyn1 | thymocyte nuclear protein 1 | 1.2 | 5.50E-02 |
| Dtymk | deoxythymidylate kinase | 1.2 | 8.30E-02 |
| Tmem254b | transmembrane protein 254b | 1.2 | 4.70E-02 |
| Ddx39 | DEAD (Asp-Glu-Ala-Asp) box polypeptide 39 | 1.2 | 7.30E-02 |
| Pls3 | plastin 3 (T-isoform) | 1.2 | 8.90E-02 |
| Gpn3 | GPN-loop GTPase 3 | 1.2 | 1.90E-02 |
| Gtf3c3 | general transcription factor IIIC, polypeptide 3 | 1.2 | 7.30E-02 |
| Rnaseh2a | ribonuclease H2, large subunit | 1.2 | 4.90E-02 |
| Casp8 | caspase 8 | 1.2 | 5.80E-02 |
| Zcchc8 | zinc finger, CCHC domain containing 8 | 1.2 | 7.90E-02 |
| BC003331 | cDNA sequence BC003331 | 1.2 | 4.40E-02 |
| Tmem129 | transmembrane protein 129 | 1.2 | 3.40E-02 |
| Stk16 | serine/threonine kinase 16 | 1.2 | 5.10E-02 |
| Heatr3 | HEAT repeat containing 3 | 1.2 | 3.20E-02 |
| Med31 | mediator complex subunit 31 | 1.2 | 8.30E-02 |
| Rqcd1 | rcd1 (required for cell differentiation) homolog 1 (S. pombe) | 1.2 | 9.20E-02 |
| Dgcr8 | DiGeorge syndrome critical region gene 8 | 1.2 | 2.60E-02 |
| Sdhd | succinate dehydrogenase complex, subunit D, integral membrane protein | 1.2 | 3.40E-02 |
| Trmt112 | tRNA methyltransferase 11-2 | 1.2 | 3.20E-02 |
| Asf1b | anti-silencing function 1B histone chaperone | 1.2 | 4.70E-02 |
| Dopey1 | dopey family member 1 | 1.2 | 6.30E-02 |
| Naa50 | N(alpha)-acetyltransferase 50, NatE catalytic subunit | 1.2 | 4.70E-02 |
| Tbcel | tubulin folding cofactor E-like | 1.2 | 4.30E-02 |
| Wrb | tryptophan rich basic protein | 1.2 | 4.60E-02 |
| Rabl3 | RAB, member RAS oncogene family-like 3 | 1.2 | 2.10E-02 |
| Cdk14 | cyclin-dependent kinase 14 | 1.2 | 9.20E-02 |
| Zbtb41 | zinc finger and BTB domain containing 41 homolog | 1.2 | 6.40E-02 |
| Fdft1 | farnesyl diphosphate farnesyl transferase 1 | 1.2 | 3.30E-02 |
| Rrp15 | ribosomal RNA processing 15 homolog (S. cerevisiae) | 1.2 | 4.90E-02 |
| Adhfe1 | alcohol dehydrogenase, iron containing, 1 | 1.2 | 5.40E-02 |
| Rfc4 | replication factor C (activator 1) 4 | 1.2 | 5.30E-02 |
| Pbx1 | pre B cell leukemia homeobox 1 | 1.2 | 6.40E-02 |
| Nudt16l1 | nudix (nucleoside diphosphate linked moiety X)-type motif 16-like 1 | 1.2 | 1.70E-02 |
| Copb2 | coatomer protein complex, subunit beta 2 (beta prime) | 1.2 | 8.60E-02 |
| Ercc6l | excision repair cross-complementing rodent repair deficiency complementation group 6 like | 1.2 | 2.30E-02 |
| Chmp2b | charged multivesicular body protein 2B | 1.2 | 7.90E-02 |
| Naa16 | N(alpha)-acetyltransferase 16, NatA auxiliary subunit | 1.2 | 5.90E-02 |
| Ctps2 | cytidine 5-triphosphate synthase 2 | 1.2 | 7.90E-02 |
| Cks1b | CDC28 protein kinase 1b | 1.2 | 7.90E-02 |
| Lactb2 | lactamase, beta 2 | 1.2 | 6.20E-02 |
| Neto2 | neuropilin (NRP) and tolloid (TLL)-like 2 | 1.2 | 7.80E-02 |
| Opa1 | optic atrophy 1 | 1.2 | 2.80E-02 |
| Ints10 | integrator complex subunit 10 | 1.2 | 4.30E-02 |
| Pan3 | PAN3 polyA specific ribonuclease subunit homolog (S. cerevisiae) | 1.2 | 9.00E-02 |
| Sumo1 | SMT3 suppressor of mif two 3 homolog 1 (yeast) | 1.2 | 3.80E-02 |
| Tomm70a | translocase of outer mitochondrial membrane 70 homolog A (yeast) | 1.2 | 5.70E-02 |
| Cox7c | cytochrome c oxidase subunit VIIc | 1.2 | 3.20E-02 |
| Ufsp1 | UFM1-specific peptidase 1 | 1.2 | 5.50E-02 |
| Usp3 | ubiquitin specific peptidase 3 | 1.2 | 7.00E-02 |
| Rcbtb1 | regulator of chromosome condensation (RCC1) and BTB (POZ) domain containing protein 1 | 1.2 | 6.50E-02 |
| Smim20 | small integral membrane protein 20 | 1.2 | 8.50E-02 |
| Ryk | receptor-like tyrosine kinase | 1.2 | 7.50E-02 |
| Hist1h2bn | histone cluster 1, H2bn | 1.2 | 5.60E-02 |
| Cdc40 | cell division cycle 40 | 1.2 | 3.40E-02 |
| Zfp113 | zinc finger protein 113 | 1.2 | 4.40E-02 |
| Cdt1 | chromatin licensing and DNA replication factor 1 | 1.2 | 8.30E-02 |
| Parn | poly(A)-specific ribonuclease (deadenylation nuclease) | 1.2 | 5.50E-02 |
| Afp | alpha fetoprotein | 1.2 | 2.70E-02 |
| Ndufa4 | NADH dehydrogenase (ubiquinone) 1 alpha subcomplex, 4 | 1.2 | 2.40E-02 |
| Gin1 | gypsy retrotransposon integrase 1 | 1.2 | 3.60E-02 |
| Ubald1 | UBA-like domain containing 1 | 1.2 | 7.90E-02 |
| Nsun3 | NOL1/NOP2/Sun domain family member 3 | 1.2 | 5.80E-02 |
| Gbas | glioblastoma amplified sequence | 1.2 | 6.00E-02 |
| Rpl3 | ribosomal protein L3 | 1.2 | 1.90E-02 |
| Ranbp6 | RAN binding protein 6 | 1.2 | 8.60E-02 |
| Mob4 | MOB family member 4, phocein | 1.2 | 5.50E-02 |
| Fgd4 | FYVE, RhoGEF and PH domain containing 4 | 1.2 | 4.30E-02 |
| Mrpl42 | mitochondrial ribosomal protein L42 | 1.2 | 9.20E-02 |
| Tmem60 | transmembrane protein 60 | 1.2 | 5.20E-02 |
| Cdk3-ps | cyclin-dependent kinase 3, pseudogene | 1.2 | 8.00E-02 |
| Mrpl1 | mitochondrial ribosomal protein L1 | 1.2 | 7.50E-02 |
| Osgep | O-sialoglycoprotein endopeptidase | 1.2 | 4.50E-02 |
| Coa3 | cytochrome C oxidase assembly factor 3 | 1.2 | 6.10E-02 |
| Gtf2a1l | general transcription factor IIA, 1-like | 1.2 | 9.50E-02 |
| Adss | adenylosuccinate synthetase, non muscle | 1.2 | 8.60E-02 |
| Psd3 | pleckstrin and Sec7 domain containing 3 | 1.2 | 7.80E-02 |
| Alg8 | asparagine-linked glycosylation 8 (alpha-1,3-glucosyltransferase) | 1.2 | 4.10E-02 |
| Fam45a | family with sequence similarity 45, member A | 1.2 | 8.10E-02 |
| Vps54 | vacuolar protein sorting 54 (yeast) | 1.2 | 5.00E-02 |
| Mak16 | MAK16 homolog (S. cerevisiae) | 1.2 | 4.20E-02 |
| Ddx18 | DEAD (Asp-Glu-Ala-Asp) box polypeptide 18 | 1.2 | 8.40E-02 |
| Idi1 | isopentenyl-diphosphate delta isomerase | 1.2 | 9.20E-02 |
| Cbx4 | chromobox 4 | 1.2 | 8.70E-02 |
| Ttc26 | tetratricopeptide repeat domain 26 | 1.2 | 9.70E-02 |
| Hps5 | Hermansky-Pudlak syndrome 5 homolog (human) | 1.2 | 6.60E-02 |
| Cnih4 | cornichon homolog 4 (Drosophila) | 1.2 | 8.10E-02 |
| Naa60 | N(alpha)-acetyltransferase 60, NatF catalytic subunit | 1.2 | 9.80E-02 |
| Cyp2d13 | cytochrome P450, family 2, subfamily d, polypeptide 13 | 1.2 | 3.10E-02 |
| Ccdc14 | coiled-coil domain containing 14 | 1.2 | 3.80E-02 |
| Nup35 | nucleoporin 35 | 1.2 | 5.80E-02 |
| Rpl35 | ribosomal protein L35 | 1.2 | 1.30E-02 |
| Pnkd | paroxysmal nonkinesiogenic dyskinesia | 1.2 | 6.00E-02 |
| Tgds | TDP-glucose 4,6-dehydratase | 1.2 | 5.00E-02 |
| Polr3gl | polymerase (RNA) III (DNA directed) polypeptide G like | 1.2 | 7.40E-02 |
| Tbc1d15 | TBC1 domain family, member 15 | 1.2 | 8.90E-02 |
| Pmpcb | peptidase (mitochondrial processing) beta | 1.2 | 1.90E-02 |
| Zfp760 | zinc finger protein 760 | 1.2 | 9.60E-02 |
| Zfp658 | zinc finger protein 658 | 1.2 | 2.90E-02 |
| Aldh2 | aldehyde dehydrogenase 2, mitochondrial | 1.2 | 3.40E-02 |
| Spice1 | spindle and centriole associated protein 1 | 1.2 | 1.30E-02 |
| Hus1 | Hus1 homolog (S. pombe) | 1.2 | 3.70E-02 |
| Son | Son DNA binding protein | 1.2 | 7.20E-02 |
| LOC102640679 | 60S ribosomal protein L29-like | 1.2 | 2.40E-02 |
| Rif1 | Rap1 interacting factor 1 homolog (yeast) | 1.2 | 4.60E-02 |
| Mrpl49 | mitochondrial ribosomal protein L49 | 1.2 | 5.40E-02 |
| Hdac2 | histone deacetylase 2 | 1.2 | 9.40E-02 |
| Bach1 | BTB and CNC homology 1 | 1.2 | 1.30E-02 |
| Sdr39u1 | short chain dehydrogenase/reductase family 39U, member 1 | 1.2 | 2.20E-02 |
| Fbxo8 | F-box protein 8 | 1.2 | 5.70E-03 |
| Yaf2 | YY1 associated factor 2 | 1.2 | 5.20E-02 |
| Echdc1 | enoyl Coenzyme A hydratase domain containing 1 | 1.2 | 3.30E-02 |
| Manf | mesencephalic astrocyte-derived neurotrophic factor | 1.2 | 4.30E-02 |
| Mier3 | mesoderm induction early response 1, family member 3 | 1.2 | 5.20E-02 |
| Prkrip1 | Prkr interacting protein 1 (IL11 inducible) | 1.2 | 5.50E-02 |
| Rrn3 | RRN3 RNA polymerase I transcription factor homolog (yeast) | 1.2 | 8.70E-03 |
| Qars | glutaminyl-tRNA synthetase | 1.2 | 9.20E-02 |
| Msrb2 | methionine sulfoxide reductase B2 | 1.2 | 7.00E-02 |
| Rsl1d1 | ribosomal L1 domain containing 1 | 1.2 | 4.40E-02 |
| Ube3a | ubiquitin protein ligase E3A | 1.2 | 2.90E-02 |
| Ranbp1 | RAN binding protein 1 | 1.2 | 2.50E-02 |
| Cenpu | centromere protein U | 1.2 | 3.30E-02 |
| Snora19 | small nucleolar RNA, H/ACA box 19 | 1.2 | 6.30E-02 |
| Mir1933 | microRNA 1933 | 1.2 | 4.80E-02 |
| Xrcc5 | X-ray repair complementing defective repair in Chinese hamster cells 5 | 1.2 | 4.30E-02 |
| Usp42 | ubiquitin specific peptidase 42 | 1.2 | 5.40E-02 |
| Dusp18 | dual specificity phosphatase 18 | 1.2 | 9.20E-02 |
| Cand1 | cullin associated and neddylation disassociated 1 | 1.2 | 7.80E-02 |
| Ccnyl1 | cyclin Y-like 1 | 1.2 | 7.80E-02 |
| Anapc13 | anaphase promoting complex subunit 13 | 1.2 | 3.70E-02 |
| Rheb | Ras homolog enriched in brain | 1.2 | 4.50E-02 |
| Rasal2 | RAS protein activator like 2 | 1.2 | 3.70E-02 |
| Tmpo | thymopoietin | 1.2 | 8.10E-02 |
| Vamp4 | vesicle-associated membrane protein 4 | 1.2 | 4.90E-02 |
| Birc5 | baculoviral IAP repeat-containing 5 | 1.2 | 8.70E-02 |
| Gart | phosphoribosylglycinamide formyltransferase | 1.2 | 7.60E-02 |
| Slc2a10 | solute carrier family 2 (facilitated glucose transporter), member 10 | 1.2 | 5.30E-02 |
| Ccdc104 | coiled-coil domain containing 104 | 1.2 | 3.70E-02 |
| Arl6 | ADP-ribosylation factor-like 6 | 1.2 | 4.30E-02 |
| Zfp296 | zinc finger protein 296 | 1.2 | 1.40E-02 |
| Snora41 | small nucleolar RNA, H/ACA box 41 | 1.2 | 7.50E-02 |
| Crls1 | cardiolipin synthase 1 | 1.2 | 3.20E-02 |
| LOC102639577 | up-regulated during skeletal muscle growth protein 5-like | 1.2 | 6.10E-02 |
| Nxt2 | nuclear transport factor 2-like export factor 2 | 1.2 | 9.30E-02 |
| Tfam | transcription factor A, mitochondrial | 1.2 | 3.70E-02 |
| Ubr1 | ubiquitin protein ligase E3 component n-recognin 1 | 1.2 | 3.80E-02 |
| Emc6 | ER membrane protein complex subunit 6 | 1.2 | 1.10E-02 |
| Mlh1 | mutL homolog 1 (E. coli) | 1.2 | 8.30E-02 |
| Sdf2l1 | stromal cell-derived factor 2-like 1 | 1.2 | 7.80E-02 |
| Fh1 | fumarate hydratase 1 | 1.3 | 1.70E-02 |
| Chchd3 | coiled-coil-helix-coiled-coil-helix domain containing 3 | 1.3 | 4.20E-02 |
| Cluap1 | clusterin associated protein 1 | 1.3 | 6.40E-02 |
| D1Ertd622e | DNA segment, Chr 1, ERATO Doi 622, expressed | 1.3 | 2.80E-02 |
| Tmem80 | transmembrane protein 80 | 1.3 | 1.30E-02 |
| Zranb2 | zinc finger, RAN-binding domain containing 2 | 1.3 | 4.70E-02 |
| Tapt1 | transmembrane anterior posterior transformation 1 | 1.3 | 6.10E-02 |
| Prmt5 | protein arginine N-methyltransferase 5 | 1.3 | 4.20E-02 |
| Them4 | thioesterase superfamily member 4 | 1.3 | 1.70E-02 |
| Lsm4 | LSM4 homolog, U6 small nuclear RNA associated (S. cerevisiae) | 1.3 | 3.50E-02 |
| Epm2aip1 | EPM2A (laforin) interacting protein 1 | 1.3 | 8.30E-02 |
| Leprel1 | leprecan-like 1 | 1.3 | 8.30E-02 |
| Cep55 | centrosomal protein 55 | 1.3 | 9.60E-02 |
| Vps36 | vacuolar protein sorting 36 (yeast) | 1.3 | 5.60E-02 |
| Gnrh1 | gonadotropin releasing hormone 1 | 1.3 | 8.20E-02 |
| Spata6 | spermatogenesis associated 6 | 1.3 | 5.80E-02 |
| Snord98 | small nucleolar RNA, C/D box 98 | 1.3 | 8.60E-02 |
| Gtf3c6 | general transcription factor IIIC, polypeptide 6, alpha | 1.3 | 6.10E-02 |
| Rpp30 | ribonuclease P/MRP 30 subunit | 1.3 | 2.00E-02 |
| AU015336 | expressed sequence AU015336 | 1.3 | 4.30E-02 |
| l7Rn6 | lethal, Chr 7, Rinchik 6 | 1.3 | 5.20E-02 |
| Rhbdd2 | rhomboid domain containing 2 | 1.3 | 2.40E-02 |
| Fdxacb1 | ferredoxin-fold anticodon binding domain containing 1 | 1.3 | 1.50E-02 |
| Spcs3 | signal peptidase complex subunit 3 homolog (S. cerevisiae) | 1.3 | 5.50E-02 |
| Atic | 5-aminoimidazole-4-carboxamide ribonucleotide formyltransferase/IMP cyclohydrolase | 1.3 | 1.70E-02 |
| Zfp68 | zinc finger protein 68 | 1.3 | 3.20E-02 |
| Ddx59 | DEAD (Asp-Glu-Ala-Asp) box polypeptide 59 | 1.3 | 2.90E-02 |
| Lmbr1 | limb region 1 | 1.3 | 1.60E-02 |
| Dzip3 | DAZ interacting protein 3, zinc finger | 1.3 | 6.60E-02 |
| Ckap2 | cytoskeleton associated protein 2 | 1.3 | 5.10E-02 |
| Lrrc58 | leucine rich repeat containing 58 | 1.3 | 2.40E-02 |
| Zfp654 | zinc finger protein 654 | 1.3 | 2.90E-02 |
| Tmem62 | transmembrane protein 62 | 1.3 | 7.10E-02 |
| Rnaseh2b | ribonuclease H2, subunit B | 1.3 | 5.90E-02 |
| Hist1h4k | histone cluster 1, H4k | 1.3 | 9.70E-03 |
| Sde2 | SDE2 telomere maintenance homolog (S. pombe) | 1.3 | 4.40E-02 |
| BC021614 | cDNA sequence BC021614 | 1.3 | 8.00E-02 |
| Ltn1 | listerin E3 ubiquitin protein ligase 1 | 1.3 | 2.00E-02 |
| Prps1 | phosphoribosyl pyrophosphate synthetase 1 | 1.3 | 1.40E-02 |
| Olfr1198 | olfactory receptor 1198 | 1.3 | 8.40E-02 |
| Comtd1 | catechol-O-methyltransferase domain containing 1 | 1.3 | 4.90E-02 |
| Spidr | scaffolding protein involved i DNA repair | 1.3 | 1.50E-02 |
| Snhg1 | small nucleolar RNA host gene (non-protein coding) 1 | 1.3 | 5.90E-02 |
| Atp2c2 | ATPase, Ca++ transporting, type 2C, member 2 | 1.3 | 4.80E-02 |
| Zfp879 | zinc finger protein 879 | 1.3 | 2.30E-02 |
| Eif2b1 | eukaryotic translation initiation factor 2B, subunit 1 (alpha) | 1.3 | 3.40E-02 |
| Far1 | fatty acyl CoA reductase 1 | 1.3 | 4.60E-02 |
| Oser1 | oxidative stress responsive serine rich 1 | 1.3 | 6.20E-02 |
| Rpf2 | ribosome production factor 2 homolog (S. cerevisiae) | 1.3 | 4.30E-02 |
| Hspa4l | heat shock protein 4 like | 1.3 | 6.50E-02 |
| Tuba4a | tubulin, alpha 4A | 1.3 | 2.10E-02 |
| Alg11 | asparagine-linked glycosylation 11 (alpha-1,2-mannosyltransferase) | 1.3 | 2.80E-02 |
| Plcxd2 | phosphatidylinositol-specific phospholipase C, X domain containing 2 | 1.3 | 1.40E-02 |
| Dact3 | dapper homolog 3, antagonist of beta-catenin (xenopus) | 1.3 | 3.00E-02 |
| Ppil3 | peptidylprolyl isomerase (cyclophilin)-like 3 | 1.3 | 2.70E-02 |
| Sec22a | SEC22 vesicle trafficking protein homolog A (S. cerevisiae) | 1.3 | 2.20E-02 |
| Znrf2 | zinc and ring finger 2 | 1.3 | 7.10E-02 |
| Prr11 | proline rich 11 | 1.3 | 4.90E-02 |
| Yars2 | tyrosyl-tRNA synthetase 2 (mitochondrial) | 1.3 | 6.30E-02 |
| AI839979 | expressed sequence AI839979 | 1.3 | 3.20E-02 |
| Ireb2 | iron responsive element binding protein 2 | 1.3 | 8.90E-02 |
| Rps27 | ribosomal protein S27 | 1.3 | 7.90E-02 |
| Atp2a2 | ATPase, Ca++ transporting, cardiac muscle, slow twitch 2 | 1.3 | 2.90E-02 |
| Dnajc15 | DnaJ (Hsp40) homolog, subfamily C, member 15 | 1.3 | 6.90E-02 |
| Sc5d | sterol-C5-desaturase (fungal ERG3, delta-5-desaturase) homolog (S. cerevisae) | 1.3 | 9.70E-02 |
| Gkap1 | G kinase anchoring protein 1 | 1.3 | 8.30E-02 |
| Tfg | Trk-fused gene | 1.3 | 1.60E-02 |
| Kntc1 | kinetochore associated 1 | 1.3 | 2.90E-02 |
| Mzt2 | mitotic spindle organizing protein 2 | 1.3 | 1.30E-02 |
| Nup160 | nucleoporin 160 | 1.3 | 1.90E-02 |
| Ppap2c | phosphatidic acid phosphatase type 2C | 1.3 | 5.60E-02 |
| Fancl | Fanconi anemia, complementation group L | 1.3 | 6.40E-03 |
| Dpp3 | dipeptidylpeptidase 3 | 1.3 | 5.10E-02 |
| Acss1 | acyl-CoA synthetase short-chain family member 1 | 1.3 | 5.80E-02 |
| Zfp12 | zinc finger protein 12 | 1.3 | 5.90E-02 |
| Insig1 | insulin induced gene 1 | 1.3 | 8.90E-02 |
| Rnf168 | ring finger protein 168 | 1.3 | 1.40E-02 |
| Bckdhb | branched chain ketoacid dehydrogenase E1, beta polypeptide | 1.3 | 5.10E-02 |
| Cycs | cytochrome c, somatic | 1.3 | 9.80E-02 |
| Maoa | monoamine oxidase A | 1.3 | 4.30E-02 |
| Usp9x | ubiquitin specific peptidase 9, X chromosome | 1.3 | 4.80E-02 |
| Perp | PERP, TP53 apoptosis effector | 1.3 | 4.90E-02 |
| Fzd3 | frizzled homolog 3 (Drosophila) | 1.3 | 8.00E-02 |
| Nt5c3 | 5-nucleotidase, cytosolic III | 1.3 | 7.90E-02 |
| Ostc | oligosaccharyltransferase complex subunit | 1.3 | 6.50E-02 |
| Cct2 | chaperonin containing Tcp1, subunit 2 (beta) | 1.3 | 4.90E-02 |
| Top2a | topoisomerase (DNA) II alpha | 1.3 | 8.30E-02 |
| Galnt11 | UDP-N-acetyl-alpha-D-galactosamine:polypeptide N-acetylgalactosaminyltransferase 11 | 1.3 | 6.00E-02 |
| Nifk | nucleolar protein interacting with the FHA domain of MKI67 | 1.3 | 3.50E-02 |
| Eid1 | EP300 interacting inhibitor of differentiation 1 | 1.3 | 5.30E-02 |
| Fam133b | family with sequence similarity 133, member B | 1.3 | 9.60E-02 |
| Gtf2h1 | general transcription factor II H, polypeptide 1 | 1.3 | 5.20E-02 |
| Sod1 | superoxide dismutase 1, soluble | 1.3 | 1.90E-02 |
| Wdr19 | WD repeat domain 19 | 1.3 | 7.70E-02 |
| Ceacam1 | carcinoembryonic antigen-related cell adhesion molecule 1 | 1.3 | 7.60E-02 |
| Fgfbp1 | fibroblast growth factor binding protein 1 | 1.3 | 4.50E-02 |
| Kpna1 | karyopherin (importin) alpha 1 | 1.3 | 1.80E-02 |
| Slc25a35 | solute carrier family 25, member 35 | 1.3 | 7.50E-02 |
| Hspe1 | heat shock protein 1 (chaperonin 10) | 1.3 | 4.40E-02 |
| Rsrc2 | arginine/serine-rich coiled-coil 2 | 1.3 | 8.20E-02 |
| Mbtps2 | membrane-bound transcription factor peptidase, site 2 | 1.3 | 5.90E-02 |
| Bpnt1 | bisphosphate 3-nucleotidase 1 | 1.3 | 1.70E-02 |
| Baiap2l1 | BAI1-associated protein 2-like 1 | 1.3 | 5.70E-02 |
| Tmem41a | transmembrane protein 41a | 1.3 | 2.50E-02 |
| Rpl21-ps3 | ribosomal protein L21, pseudogene 3 | 1.3 | 3.60E-02 |
| Pccb | propionyl Coenzyme A carboxylase, beta polypeptide | 1.3 | 4.70E-02 |
| Thap2 | THAP domain containing, apoptosis associated protein 2 | 1.3 | 1.00E-01 |
| Cuedc2 | CUE domain containing 2 | 1.3 | 3.30E-02 |
| Wdr82 | WD repeat domain containing 82 | 1.3 | 5.50E-02 |
| Rab28 | RAB28, member RAS oncogene family | 1.3 | 9.90E-03 |
| Aldh1a7 | aldehyde dehydrogenase family 1, subfamily A7 | 1.3 | 9.40E-02 |
| Ak3 | adenylate kinase 3 | 1.3 | 9.90E-03 |
| BC016579 | cDNA sequence, BC016579 | 1.3 | 9.10E-02 |
| Avpi1 | arginine vasopressin-induced 1 | 1.3 | 4.00E-02 |
| Eif1ax | eukaryotic translation initiation factor 1A, X-linked | 1.3 | 8.40E-02 |
| Qtrtd1 | queuine tRNA-ribosyltransferase domain containing 1 | 1.3 | 1.90E-02 |
| LOC102639915 | 60S ribosomal protein L29-like | 1.3 | 1.70E-02 |
| Nusap1 | nucleolar and spindle associated protein 1 | 1.3 | 3.60E-02 |
| Fam86 | family with sequence similarity 86 | 1.3 | 2.20E-02 |
| Ap5m1 | adaptor-related protein complex 5, mu 1 subunit | 1.3 | 1.60E-02 |
| Rnf141 | ring finger protein 141 | 1.3 | 5.20E-02 |
| Rnase2b | ribonuclease, RNase A family, 2B (liver, eosinophil-derived neurotoxin) | 1.3 | 3.10E-02 |
| Zfp759 | zinc finger protein 759 | 1.3 | 4.70E-02 |
| Slc7a7 | solute carrier family 7 (cationic amino acid transporter, y+ system), member 7 | 1.3 | 2.80E-02 |
| LOC102636905 | uncharacterized LOC102636905 | 1.3 | 4.80E-02 |
| Gstt1 | glutathione S-transferase, theta 1 | 1.3 | 5.20E-02 |
| Rpap2 | RNA polymerase II associated protein 2 | 1.3 | 1.40E-02 |
| Rbmxl1 | RNA binding motif protein, X linked-like-1 | 1.3 | 2.30E-02 |
| Glmn | glomulin, FKBP associated protein | 1.3 | 3.30E-02 |
| Ccpg1os | cell cycle progression 1, opposite strand | 1.3 | 6.30E-02 |
| Naa25 | N(alpha)-acetyltransferase 25, NatB auxiliary subunit | 1.3 | 8.10E-03 |
| Satb2 | special AT-rich sequence binding protein 2 | 1.3 | 6.60E-02 |
| Nup205 | nucleoporin 205 | 1.3 | 6.50E-02 |
| Thumpd1 | THUMP domain containing 1 | 1.3 | 4.90E-02 |
| Bbs5 | Bardet-Biedl syndrome 5 (human) | 1.3 | 2.30E-02 |
| Rbm6 | RNA binding motif protein 6 | 1.3 | 5.50E-02 |
| Acat1 | acetyl-Coenzyme A acetyltransferase 1 | 1.3 | 4.30E-02 |
| Chchd4 | coiled-coil-helix-coiled-coil-helix domain containing 4 | 1.3 | 1.20E-02 |
| Bambi | BMP and activin membrane-bound inhibitor | 1.3 | 6.40E-02 |
| Fastkd2 | FAST kinase domains 2 | 1.3 | 6.70E-03 |
| Ulbp1 | UL16 binding protein 1 | 1.3 | 9.10E-02 |
| Tipin | timeless interacting protein | 1.3 | 9.30E-02 |
| Paics | phosphoribosylaminoimidazole carboxylase, phosphoribosylaminoribosylaminoimidazole, succinocarboxamide synthetase | 1.3 | 1.60E-02 |
| Irf6 | interferon regulatory factor 6 | 1.3 | 2.30E-02 |
| Sgms1 | sphingomyelin synthase 1 | 1.3 | 4.70E-02 |
| Dse | dermatan sulfate epimerase | 1.3 | 7.70E-02 |
| Galnt3 | UDP-N-acetyl-alpha-D-galactosamine:polypeptide N-acetylgalactosaminyltransferase 3 | 1.3 | 9.10E-02 |
| Atg4a | autophagy related 4A, cysteine peptidase | 1.3 | 7.30E-02 |
| Rpl29 | ribosomal protein L29 | 1.3 | 9.60E-03 |
| Lipt1 | lipoyltransferase 1 | 1.3 | 2.60E-03 |
| Atr | ataxia telangiectasia and Rad3 related | 1.3 | 1.40E-02 |
| Timm8b | translocase of inner mitochondrial membrane 8B | 1.3 | 2.90E-02 |
| Casc5 | cancer susceptibility candidate 5 | 1.3 | 3.80E-02 |
| Gstm4 | glutathione S-transferase, mu 4 | 1.3 | 5.60E-02 |
| Mkks | McKusick-Kaufman syndrome | 1.3 | 2.70E-02 |
| Napepld | N-acyl phosphatidylethanolamine phospholipase D | 1.3 | 5.50E-02 |
| Dkc1 | dyskeratosis congenita 1, dyskerin | 1.3 | 9.00E-02 |
| Rnf32 | ring finger protein 32 | 1.3 | 5.60E-02 |
| Prb1 | proline-rich protein BstNI subfamily 1 | 1.3 | 8.10E-02 |
| Sox3 | SRY (sex determining region Y)-box 3 | 1.3 | 6.20E-02 |
| Atp8a2 | ATPase, aminophospholipid transporter-like, class I, type 8A, member 2 | 1.3 | 8.40E-02 |
| Gpr125 | G protein-coupled receptor 125 | 1.3 | 9.50E-02 |
| Poglut1 | protein O-glucosyltransferase 1 | 1.3 | 1.90E-02 |
| Trmt2b | TRM2 tRNA methyltransferase 2B | 1.3 | 4.60E-02 |
| Mir383 | microRNA 383 | 1.3 | 4.70E-02 |
| Rad9b | RAD9 homolog B | 1.3 | 2.70E-02 |
| Hibch | 3-hydroxyisobutyryl-Coenzyme A hydrolase | 1.3 | 6.20E-03 |
| Slc25a32 | solute carrier family 25, member 32 | 1.3 | 3.80E-03 |
| Slc15a2 | solute carrier family 15 (H+/peptide transporter), member 2 | 1.3 | 6.70E-02 |
| Tceb1 | transcription elongation factor B (SIII), polypeptide 1 | 1.3 | 7.90E-02 |
| Cct6a | chaperonin containing Tcp1, subunit 6a (zeta) | 1.3 | 3.80E-02 |
| Lancl2 | LanC (bacterial lantibiotic synthetase component C)-like 2 | 1.3 | 2.70E-02 |
| Mbl1 | mannose-binding lectin (protein A) 1 | 1.3 | 2.70E-02 |
| Dnm1l | dynamin 1-like | 1.3 | 1.50E-02 |
| Pbk | PDZ binding kinase | 1.3 | 6.30E-02 |
| Apool | apolipoprotein O-like | 1.3 | 4.40E-02 |
| Kitl | kit ligand | 1.3 | 2.40E-02 |
| Bbip1 | BBSome interacting protein 1 | 1.3 | 7.80E-02 |
| Cers3 | ceramide synthase 3 | 1.3 | 9.60E-02 |
| Stip1 | stress-induced phosphoprotein 1 | 1.3 | 7.30E-02 |
| Mrpl32 | mitochondrial ribosomal protein L32 | 1.3 | 4.60E-02 |
| Ttk | Ttk protein kinase | 1.3 | 4.70E-02 |
| Ppp2r3a | protein phosphatase 2, regulatory subunit B, alpha | 1.3 | 2.70E-02 |
| Fgfrl1 | fibroblast growth factor receptor-like 1 | 1.3 | 2.20E-02 |
| Idh1 | isocitrate dehydrogenase 1 (NADP+), soluble | 1.3 | 1.20E-02 |
| Abhd5 | abhydrolase domain containing 5 | 1.3 | 6.50E-02 |
| Ahsa2 | AHA1, activator of heat shock protein ATPase 2 | 1.3 | 4.30E-02 |
| Neu2 | neuraminidase 2 | 1.3 | 1.60E-02 |
| D16Ertd472e | DNA segment, Chr 16, ERATO Doi 472, expressed | 1.3 | 4.60E-02 |
| Tmem33 | transmembrane protein 33 | 1.3 | 4.00E-03 |
| Ube4a | ubiquitination factor E4A | 1.3 | 1.50E-02 |
| Kcnh7 | potassium voltage-gated channel, subfamily H (eag-related), member 7 | 1.3 | 6.50E-02 |
| Hnf1a | HNF1 homeobox A | 1.3 | 9.80E-02 |
| Olfr1424 | olfactory receptor 1424 | 1.3 | 8.70E-02 |
| Bora | bora, aurora kinase A activator | 1.3 | 2.00E-02 |
| Mis18a | MIS18 kinetochore protein homolog A (S. pombe) | 1.3 | 5.60E-03 |
| Tlr3 | toll-like receptor 3 | 1.3 | 3.90E-02 |
| Hist4h4 | histone cluster 4, H4 | 1.3 | 5.90E-03 |
| Vprbp | Vpr (HIV-1) binding protein | 1.3 | 5.40E-03 |
| Snx16 | sorting nexin 16 | 1.3 | 5.20E-02 |
| Nkap | NFKB activating protein | 1.3 | 1.80E-02 |
| Zfp119b | zinc finger protein 119b | 1.3 | 7.90E-02 |
| Abhd6 | abhydrolase domain containing 6 | 1.3 | 8.60E-02 |
| Mcm4 | minichromosome maintenance deficient 4 homolog (S. cerevisiae) | 1.3 | 3.00E-02 |
| Prr16 | proline rich 16 | 1.3 | 3.30E-02 |
| Actr6 | ARP6 actin-related protein 6 | 1.3 | 4.50E-02 |
| Cc2d2a | coiled-coil and C2 domain containing 2A | 1.3 | 4.70E-02 |
| Tma7 | translational machinery associated 7 homolog (S. cerevisiae) | 1.3 | 5.10E-02 |
| Yes1 | Yamaguchi sarcoma viral (v-yes) oncogene homolog 1 | 1.3 | 2.90E-02 |
| Vmn2r79 | vomeronasal 2, receptor 79 | 1.3 | 6.70E-03 |
| Gtpbp8 | GTP-binding protein 8 (putative) | 1.3 | 1.30E-02 |
| Tfb2m | transcription factor B2, mitochondrial | 1.3 | 1.40E-02 |
| Gtf3a | general transcription factor III A | 1.3 | 1.60E-02 |
| Zfp54 | zinc finger protein 54 | 1.3 | 5.70E-02 |
| Fgfr2 | fibroblast growth factor receptor 2 | 1.3 | 5.20E-02 |
| Mcm5 | minichromosome maintenance deficient 5, cell division cycle 46 (S. cerevisiae) | 1.3 | 1.50E-02 |
| Abcb7 | ATP-binding cassette, sub-family B (MDR/TAP), member 7 | 1.3 | 5.50E-03 |
| Ncaph | non-SMC condensin I complex, subunit H | 1.3 | 8.10E-02 |
| Hyal2 | hyaluronoglucosaminidase 2 | 1.3 | 7.00E-02 |
| Rab34 | RAB34, member RAS oncogene family | 1.3 | 4.20E-02 |
| Rundc3a | RUN domain containing 3A | 1.3 | 1.90E-02 |
| Tceal1 | transcription elongation factor A (SII)-like 1 | 1.3 | 7.10E-02 |
| Adam22 | a disintegrin and metallopeptidase domain 22 | 1.3 | 1.70E-02 |
| Uggt2 | UDP-glucose glycoprotein glucosyltransferase 2 | 1.3 | 9.50E-02 |
| Fgfr3 | fibroblast growth factor receptor 3 | 1.3 | 3.30E-02 |
| Ptplb | protein tyrosine phosphatase-like (proline instead of catalytic arginine), member b | 1.3 | 1.70E-02 |
| Trim35 | tripartite motif-containing 35 | 1.3 | 5.60E-02 |
| Slamf9 | SLAM family member 9 | 1.3 | 3.40E-02 |
| Olfr310 | olfactory receptor 310 | 1.3 | 1.30E-02 |
| Myh7 | myosin, heavy polypeptide 7, cardiac muscle, beta | 1.3 | 4.30E-02 |
| Pkib | protein kinase inhibitor beta, cAMP dependent, testis specific | 1.3 | 4.50E-03 |
| Opn3 | opsin 3 | 1.3 | 2.10E-02 |
| Zfp873 | zinc finger protein 873 | 1.3 | 8.20E-02 |
| Zfp677 | zinc finger protein 677 | 1.3 | 9.50E-02 |
| Mir1931 | microRNA 1931 | 1.3 | 7.30E-02 |
| Slc5a3 | solute carrier family 5 (inositol transporters), member 3 | 1.3 | 6.70E-02 |
| Taf1d | TATA box binding protein (Tbp)-associated factor, RNA polymerase I, D | 1.3 | 9.90E-02 |
| Rfx3 | regulatory factor X, 3 (influences HLA class II expression) | 1.3 | 2.10E-03 |
| Myef2 | myelin basic protein expression factor 2, repressor | 1.3 | 4.40E-02 |
| Rrm1 | ribonucleotide reductase M1 | 1.4 | 6.20E-03 |
| Mir3962 | microRNA 3962 | 1.4 | 4.70E-02 |
| Mthfd2l | methylenetetrahydrofolate dehydrogenase (NADP+ dependent) 2-like | 1.4 | 6.30E-02 |
| Ift22 | intraflagellar transport 22 | 1.4 | 9.80E-03 |
| Fundc1 | FUN14 domain containing 1 | 1.4 | 2.10E-02 |
| Gtpbp10 | GTP-binding protein 10 (putative) | 1.4 | 4.70E-03 |
| Guf1 | GUF1 GTPase homolog (S. cerevisiae) | 1.4 | 1.80E-02 |
| Bet1 | blocked early in transport 1 homolog (S. cerevisiae) | 1.4 | 3.70E-02 |
| Chordc1 | cysteine and histidine-rich domain (CHORD)-containing, zinc-binding protein 1 | 1.4 | 6.70E-02 |
| Pkd2 | polycystic kidney disease 2 | 1.4 | 4.40E-02 |
| Gpt2 | glutamic pyruvate transaminase (alanine aminotransferase) 2 | 1.4 | 6.40E-03 |
| Zfp157 | zinc finger protein 157 | 1.4 | 2.50E-02 |
| Chaf1b | chromatin assembly factor 1, subunit B (p60) | 1.4 | 7.50E-03 |
| Ccdc122 | coiled-coil domain containing 122 | 1.4 | 3.00E-03 |
| Tbc1d19 | TBC1 domain family, member 19 | 1.4 | 5.90E-03 |
| Esd | esterase D/formylglutathione hydrolase | 1.4 | 6.70E-02 |
| Pigx | phosphatidylinositol glycan anchor biosynthesis, class X | 1.4 | 4.20E-02 |
| Zbtb39 | zinc finger and BTB domain containing 39 | 1.4 | 8.40E-02 |
| Zfp747 | zinc finger protein 747 | 1.4 | 2.10E-02 |
| Cd200 | CD200 antigen | 1.4 | 4.80E-02 |
| Tob1 | transducer of ErbB-2.1 | 1.4 | 4.00E-03 |
| Ddo | D-aspartate oxidase | 1.4 | 3.70E-02 |
| Cct8 | chaperonin containing Tcp1, subunit 8 (theta) | 1.4 | 5.60E-03 |
| Oaz1 | ornithine decarboxylase antizyme 1 | 1.4 | 1.50E-02 |
| Met | met proto-oncogene | 1.4 | 2.10E-02 |
| Rp9 | retinitis pigmentosa 9 (human) | 1.4 | 2.60E-02 |
| Ugp2 | UDP-glucose pyrophosphorylase 2 | 1.4 | 1.70E-02 |
| Hibadh | 3-hydroxyisobutyrate dehydrogenase | 1.4 | 3.20E-02 |
| Frk | fyn-related kinase | 1.4 | 2.40E-02 |
| Snord14a | small nucleolar RNA, C/D box 14A | 1.4 | 5.00E-02 |
| Hist2h2bb | histone cluster 2, H2bb | 1.4 | 3.00E-02 |
| Cd36 | CD36 antigen | 1.4 | 9.00E-02 |
| Iqcb1 | IQ calmodulin-binding motif containing 1 | 1.4 | 3.80E-03 |
| Ddx26b | DEAD/H (Asp-Glu-Ala-Asp/His) box polypeptide 26B | 1.4 | 3.70E-02 |
| Snora69 | small nucleolar RNA, H/ACA box 69 | 1.4 | 5.50E-02 |
| E2f8 | E2F transcription factor 8 | 1.4 | 2.20E-02 |
| Padi4 | peptidyl arginine deiminase, type IV | 1.4 | 7.70E-02 |
| Nit2 | nitrilase family, member 2 | 1.4 | 2.40E-03 |
| Mcee | methylmalonyl CoA epimerase | 1.4 | 3.90E-03 |
| Aim1 | absent in melanoma 1 | 1.4 | 8.90E-02 |
| Ibtk | inhibitor of Bruton agammaglobulinemia tyrosine kinase | 1.4 | 4.50E-02 |
| Hells | helicase, lymphoid specific | 1.4 | 5.40E-03 |
| Imp3 | IMP3, U3 small nucleolar ribonucleoprotein, homolog (yeast) | 1.4 | 4.10E-02 |
| C1galt1 | core 1 synthase, glycoprotein-N-acetylgalactosamine 3-beta-galactosyltransferase, 1 | 1.4 | 9.40E-03 |
| Fmod | fibromodulin | 1.4 | 3.90E-02 |
| Cyb5r4 | cytochrome b5 reductase 4 | 1.4 | 6.20E-03 |
| Man1a | mannosidase 1, alpha | 1.4 | 9.50E-02 |
| Cldn25 | claudin 25 | 1.4 | 1.70E-02 |
| Cdk1 | cyclin-dependent kinase 1 | 1.4 | 2.50E-02 |
| Gstm1 | glutathione S-transferase, mu 1 | 1.4 | 2.10E-02 |
| Aasdhppt | aminoadipate-semialdehyde dehydrogenase-phosphopantetheinyl transferase | 1.4 | 1.50E-02 |
| Abhd14b | abhydrolase domain containing 14b | 1.4 | 5.20E-03 |
| Micu3 | mitochondrial calcium uptake family, member 3 | 1.4 | 2.60E-02 |
| Pole | polymerase (DNA directed), epsilon | 1.4 | 5.20E-02 |
| Rpl21-ps12 | ribosomal protein L21, pseudogene 12 | 1.4 | 5.60E-02 |
| Cideb | cell death-inducing DNA fragmentation factor, alpha subunit-like effector B | 1.4 | 4.20E-02 |
| Dtl | denticleless homolog (Drosophila) | 1.4 | 1.70E-02 |
| Jag1 | jagged 1 | 1.4 | 8.80E-02 |
| Cox20 | COX20 Cox2 chaperone | 1.4 | 7.30E-03 |
| Sik2 | salt inducible kinase 2 | 1.4 | 9.70E-02 |
| Zfp708 | zinc finger protein 708 | 1.4 | 7.50E-03 |
| Tsr2 | TSR2 20S rRNA accumulation | 1.4 | 1.10E-02 |
| Mir218-1 | microRNA 218-1 | 1.4 | 7.40E-02 |
| Pter | phosphotriesterase related | 1.4 | 4.90E-02 |
| Bub1 | budding uninhibited by benzimidazoles 1 homolog (S. cerevisiae) | 1.4 | 2.80E-02 |
| Mir181b-2 | microRNA 181b-2 | 1.4 | 2.90E-02 |
| Hspd1 | heat shock protein 1 (chaperonin) | 1.4 | 2.00E-02 |
| Thrb | thyroid hormone receptor beta | 1.4 | 2.50E-02 |
| Mospd2 | motile sperm domain containing 2 | 1.4 | 3.50E-02 |
| Cacybp | calcyclin binding protein | 1.4 | 2.30E-02 |
| Olfr467 | olfactory receptor 467 | 1.4 | 9.50E-02 |
| Irx2 | Iroquois related homeobox 2 (Drosophila) | 1.4 | 5.40E-02 |
| Mir666 | microRNA 666 | 1.4 | 3.10E-03 |
| Mpzl3 | myelin protein zero-like 3 | 1.4 | 9.10E-02 |
| Snora81 | small nucleolar RNA, H/ACA box 81 | 1.4 | 7.30E-02 |
| Tmem126a | transmembrane protein 126A | 1.4 | 2.60E-03 |
| Rpl21-ps4 | ribosomal protein L21, pseudogene 4 | 1.4 | 5.90E-02 |
| Oog3 | oogenesin 3 | 1.4 | 3.80E-02 |
| Mki67 | antigen identified by monoclonal antibody Ki 67 | 1.4 | 3.30E-02 |
| Gsta4 | glutathione S-transferase, alpha 4 | 1.4 | 4.40E-02 |
| Cyp2r1 | cytochrome P450, family 2, subfamily r, polypeptide 1 | 1.4 | 4.40E-02 |
| Ppm1k | protein phosphatase 1K (PP2C domain containing) | 1.4 | 3.50E-02 |
| Stx19 | syntaxin 19 | 1.4 | 3.50E-02 |
| Slc25a37 | solute carrier family 25, member 37 | 1.4 | 2.00E-02 |
| Msl3 | male-specific lethal 3 homolog (Drosophila) | 1.4 | 6.90E-03 |
| Ptp4a1 | protein tyrosine phosphatase 4a1 | 1.4 | 6.00E-03 |
| Hddc2 | HD domain containing 2 | 1.4 | 1.60E-03 |
| Ttc21b | tetratricopeptide repeat domain 21B | 1.4 | 3.90E-02 |
| Mcm7 | minichromosome maintenance deficient 7 (S. cerevisiae) | 1.4 | 8.20E-02 |
| Vamp7 | vesicle-associated membrane protein 7 | 1.4 | 1.30E-02 |
| Pbx3 | pre B cell leukemia homeobox 3 | 1.4 | 1.20E-02 |
| AU041133 | expressed sequence AU041133 | 1.4 | 1.60E-02 |
| Ivns1abp | influenza virus NS1A binding protein | 1.4 | 3.70E-02 |
| Rcan1 | regulator of calcineurin 1 | 1.4 | 3.20E-02 |
| Tfap4 | transcription factor AP4 | 1.4 | 1.60E-02 |
| Peg3os | Peg3 opposite strand | 1.4 | 9.90E-02 |
| Pls1 | plastin 1 (I-isoform) | 1.4 | 5.20E-02 |
| Gimap9 | GTPase, IMAP family member 9 | 1.4 | 9.30E-02 |
| Ift81 | intraflagellar transport 81 | 1.4 | 3.30E-03 |
| Ice2 | interactor of little elongation complex ELL subunit 2 | 1.4 | 3.60E-02 |
| Mfap3l | microfibrillar-associated protein 3-like | 1.4 | 4.00E-02 |
| Plch1 | phospholipase C, eta 1 | 1.5 | 4.50E-02 |
| Mum1l1 | melanoma associated antigen (mutated) 1-like 1 | 1.5 | 4.50E-02 |
| Sytl2 | synaptotagmin-like 2 | 1.5 | 1.70E-02 |
| Bin1 | bridging integrator 1 | 1.5 | 5.10E-02 |
| Prdm5 | PR domain containing 5 | 1.5 | 7.20E-02 |
| Cdca7 | cell division cycle associated 7 | 1.5 | 5.30E-02 |
| Mrps36-ps1 | mitichondrial ribosomal protein S36, pseudogene 1 | 1.5 | 2.50E-02 |
| Ly96 | lymphocyte antigen 96 | 1.5 | 6.10E-03 |
| Ace2 | angiotensin I converting enzyme (peptidyl-dipeptidase A) 2 | 1.5 | 5.10E-02 |
| Psat1 | phosphoserine aminotransferase 1 | 1.5 | 2.30E-02 |
| Gpam | glycerol-3-phosphate acyltransferase, mitochondrial | 1.5 | 4.60E-02 |
| Slc18b1 | solute carrier family 18, subfamily B, member 1 | 1.5 | 1.50E-03 |
| Serpina7 | serine (or cysteine) peptidase inhibitor, clade A (alpha-1 antiproteinase, antitrypsin), member 7 | 1.5 | 6.10E-02 |
| Mzt1 | mitotic spindle organizing protein 1 | 1.5 | 7.30E-04 |
| Smim6 | small integral membrane protein 6 | 1.5 | 5.50E-02 |
| Gyk | glycerol kinase | 1.5 | 5.30E-03 |
| Ces1d | carboxylesterase 1D | 1.5 | 3.20E-02 |
| Cenpn | centromere protein N | 1.5 | 2.60E-03 |
| Rps19-ps3 | ribosomal protein S19, pseudogene 3 | 1.5 | 1.40E-02 |
| Snora15 | small nucleolar RNA, H/ACA box 15 | 1.5 | 5.00E-02 |
| Cpox | coproporphyrinogen oxidase | 1.5 | 7.30E-02 |
| Cks2 | CDC28 protein kinase regulatory subunit 2 | 1.5 | 5.20E-03 |
| Stambpl1 | STAM binding protein like 1 | 1.5 | 2.90E-02 |
| Sgsm1 | small G protein signaling modulator 1 | 1.5 | 1.20E-02 |
| Mybpc2 | myosin binding protein C, fast-type | 1.5 | 8.40E-02 |
| Gas6 | growth arrest specific 6 | 1.5 | 1.40E-02 |
| Mir19a | microRNA 19a | 1.5 | 8.20E-02 |
| Hist1h2ab | histone cluster 1, H2ab | 1.5 | 4.20E-02 |
| Tst | thiosulfate sulfurtransferase, mitochondrial | 1.5 | 3.80E-03 |
| Npas2 | neuronal PAS domain protein 2 | 1.5 | 9.80E-02 |
| Ssbp2 | single-stranded DNA binding protein 2 | 1.5 | 8.30E-02 |
| Naaladl2 | N-acetylated alpha-linked acidic dipeptidase-like 2 | 1.5 | 8.60E-03 |
| Igfbp6 | insulin-like growth factor binding protein 6 | 1.5 | 7.70E-02 |
| Slc16a1 | solute carrier family 16 (monocarboxylic acid transporters), member 1 | 1.5 | 5.80E-03 |
| Mbnl3 | muscleblind-like 3 (Drosophila) | 1.5 | 1.50E-02 |
| Slc30a4 | solute carrier family 30 (zinc transporter), member 4 | 1.5 | 6.20E-03 |
| Prps2 | phosphoribosyl pyrophosphate synthetase 2 | 1.5 | 1.40E-02 |
| Gprc5b | G protein-coupled receptor, family C, group 5, member B | 1.5 | 1.00E-02 |
| Morc4 | microrchidia 4 | 1.5 | 6.80E-02 |
| Akr1c18 | aldo-keto reductase family 1, member C18 | 1.5 | 1.20E-02 |
| Bgn | biglycan | 1.6 | 2.10E-02 |
| Scgb1c1 | secretoglobin, family 1C, member 1 | 1.6 | 6.10E-02 |
| Fam13a | family with sequence similarity 13, member A | 1.6 | 2.60E-02 |
| Fmo4 | flavin containing monooxygenase 4 | 1.6 | 3.40E-02 |
| Adh1 | alcohol dehydrogenase 1 (class I) | 1.6 | 9.30E-02 |
| Mgst2 | microsomal glutathione S-transferase 2 | 1.6 | 4.50E-02 |
| Rps13 | ribosomal protein S13 | 1.6 | 8.20E-03 |
| Gpr128 | G protein-coupled receptor 128 | 1.6 | 9.40E-02 |
| Muc5ac | mucin 5, subtypes A and C, tracheobronchial/gastric | 1.6 | 4.40E-02 |
| Ankrd22 | ankyrin repeat domain 22 | 1.6 | 8.20E-02 |
| Uhrf2 | ubiquitin-like, containing PHD and RING finger domains 2 | 1.6 | 7.60E-03 |
| St3gal6 | ST3 beta-galactoside alpha-2,3-sialyltransferase 6 | 1.6 | 4.10E-02 |
| Wee1 | WEE 1 homolog 1 (S. pombe) | 1.7 | 2.20E-03 |
| Suclg2 | succinate-Coenzyme A ligase, GDP-forming, beta subunit | 1.7 | 3.10E-02 |
| Mtmr11 | myotubularin related protein 11 | 1.7 | 9.00E-02 |
| Akr1c19 | aldo-keto reductase family 1, member C19 | 1.7 | 2.60E-02 |
| Vmn2r8 | vomeronasal 2, receptor 8 | 1.7 | 8.60E-02 |
| Snord16a | small nucleolar RNA, C/D box 16A | 1.7 | 6.10E-03 |
| Cd38 | CD38 antigen | 1.7 | 1.20E-03 |
| Gsto1 | glutathione S-transferase omega 1 | 1.7 | 7.00E-02 |
| Hsph1 | heat shock 105kDa/110kDa protein 1 | 1.7 | 6.40E-02 |
| Pof1b | premature ovarian failure 1B | 1.7 | 1.60E-02 |
| Slc16a7 | solute carrier family 16 (monocarboxylic acid transporters), member 7 | 1.7 | 5.60E-03 |
| Fam83b | family with sequence similarity 83, member B | 1.7 | 2.10E-02 |
| Chml | choroideremia-like | 1.7 | 4.60E-02 |
| Bmp3 | bone morphogenetic protein 3 | 1.7 | 1.20E-03 |
| Klhl13 | kelch-like 13 | 1.8 | 2.10E-02 |
| Ptprz1 | protein tyrosine phosphatase, receptor type Z, polypeptide 1 | 1.8 | 4.60E-02 |
| LOC102633750 | zinc finger protein 14-like | 1.8 | 3.80E-03 |
| Pde7b | phosphodiesterase 7B | 1.8 | 9.30E-02 |
| Slc35g2 | solute carrier family 35, member G2 | 1.8 | 2.70E-03 |
| Tmem171 | transmembrane protein 171 | 1.8 | 9.90E-03 |
| Aass | aminoadipate-semialdehyde synthase | 1.8 | 7.30E-02 |
| Tox3 | TOX high mobility group box family member 3 | 1.8 | 4.00E-02 |
| Cyp3a13 | cytochrome P450, family 3, subfamily a, polypeptide 13 | 1.8 | 5.00E-02 |
| Hist2h2ab | histone cluster 2, H2ab | 1.8 | 4.20E-03 |
| Urah | urate (5-hydroxyiso-) hydrolase | 1.8 | 5.70E-03 |
| Pipox | pipecolic acid oxidase | 1.8 | 6.00E-02 |
| Fam20a | family with sequence similarity 20, member A | 1.8 | 3.80E-02 |
| Fmo2 | flavin containing monooxygenase 2 | 1.8 | 5.10E-02 |
| Akr1c13 | aldo-keto reductase family 1, member C13 | 1.8 | 1.90E-02 |
| Slc35f1 | solute carrier family 35, member F1 | 1.8 | 9.30E-02 |
| Etv1 | ets variant 1 | 1.8 | 4.20E-02 |
| Gsta3 | glutathione S-transferase, alpha 3 | 1.9 | 8.00E-03 |
| Prg4 | proteoglycan 4 (megakaryocyte stimulating factor, articular superficial zone protein) | 1.9 | 4.80E-02 |
| Rbp4 | retinol binding protein 4, plasma | 1.9 | 2.20E-02 |
| Zfp97 | zinc finger protein 97 | 1.9 | 1.40E-02 |
| Slc40a1 | solute carrier family 40 (iron-regulated transporter), member 1 | 2.1 | 3.40E-02 |
| Cyp2c68 | cytochrome P450, family 2, subfamily c, polypeptide 68 | 2.1 | 4.10E-02 |
| Pdcd4 | programmed cell death 4 | 2.2 | 7.90E-02 |
| LOC102642047 | uncharacterized LOC102642047 | 2.4 | 5.00E-02 |
| Cldn8 | claudin 8 | 2.4 | 3.00E-03 |
| Serpinb5 | serine (or cysteine) peptidase inhibitor, clade B, member 5 | 2.4 | 1.50E-02 |
| Mir493 | microRNA 493 | 2.6 | 3.60E-02 |
| Cd24a | CD24a antigen | 2.6 | 5.50E-04 |
| Muc5b | mucin 5, subtype B, tracheobronchial | 3.1 | 6.80E-02 |
| Alb | albumin | 3.1 | 4.30E-02 |

*fc:* fold change, *KD:* overexpression
