# Supplementary material for: Snail mediates repression of the Dlk1-Dio3 locus in lung tumor-infiltrating immune cells
Source: Oncotarget. 2018 Aug 17;9(64):32331–45. doi: 10.18632/oncotarget.25965 (PMC6122344; doi:10.18632/oncotarget.25965)
Supplement: Supplementary file 4 [file oncotarget-09-32331-s004.docx]

**Supplementary Table 3: List of 114 genes in the intersection of “Snail repressed” genes from Figure 1A**

| **Symbol** | **Description** | **OE: fc** | **OE: p-value** | **KD: fc** | **KD: p-value** |
| --- | --- | --- | --- | --- | --- |
| Mir377 | microRNA 377 | -3.4 | 4.00E-02 | 2.2 | 9.10E-02 |
| Fras1 | Fraser syndrome 1 homolog (human) | -3.2 | 4.10E-02 | 1.8 | 7.70E-02 |
| Mir485 | microRNA 485 | -3 | 4.50E-02 | 2.3 | 3.30E-02 |
| Mir453 | microRNA 453 | -3 | 1.60E-02 | 2.2 | 2.20E-02 |
| Mir323 | microRNA 323 | -3 | 7.10E-02 | 2.6 | 3.70E-02 |
| Mir300 | microRNA 300 | -2.5 | 6.00E-02 | 2.2 | 8.90E-02 |
| Mir382 | microRNA 382 | -2.4 | 6.30E-02 | 2.1 | 5.10E-02 |
| AF357425 | snoRNA AF357425 | -2.4 | 2.80E-02 | 2.1 | 2.90E-02 |
| Mir487b | microRNA 487b | -2.4 | 1.30E-02 | 2 | 3.80E-02 |
| Mir544 | microRNA 544 | -2.4 | 4.30E-02 | 2.1 | 2.90E-02 |
| AF357426 | snoRNA AF357426 | -2.3 | 5.60E-02 | 2.9 | 5.70E-03 |
| Rasd2 | RASD family, member 2 | -2.3 | 5.20E-02 | 1.7 | 5.20E-02 |
| DQ267102 | snoRNA DQ267102 | -2.2 | 2.00E-02 | 2 | 3.00E-02 |
| DQ267100 | snoRNA DQ267100 | -2.2 | 2.00E-02 | 2 | 2.00E-02 |
| Cldn2 | claudin 2 | -2.2 | 1.50E-04 | 1.3 | 6.60E-02 |
| Mir668 | microRNA 668 | -2.2 | 3.00E-02 | 1.5 | 9.40E-02 |
| Mir1193 | microRNA 1193 | -2.1 | 3.30E-02 | 1.7 | 3.60E-02 |
| Arhgef38 | Rho guanine nucleotide exchange factor (GEF) 38 | -2.1 | 2.10E-02 | 1.4 | 2.70E-02 |
| DQ267101 | snoRNA DQ267101 | -2.1 | 3.40E-02 | 1.7 | 5.00E-02 |
| Rian | RNA imprinted and accumulated in nucleus | -2 | 3.50E-02 | 2 | 4.00E-02 |
| Itih2 | inter-alpha trypsin inhibitor, heavy chain 2 | -2 | 9.40E-02 | 2.1 | 3.20E-02 |
| Mir3070b | microRNA 3070b | -2 | 6.60E-02 | 1.5 | 9.70E-02 |
| Meg3 | maternally expressed 3 | -1.9 | 4.20E-02 | 1.9 | 5.20E-02 |
| Mir654 | microRNA 654 | -1.9 | 8.90E-02 | 1.7 | 4.10E-02 |
| AF357355 | snoRNA AF357355 | -1.9 | 4.20E-02 | 1.8 | 3.50E-02 |
| Hist1h2bk | histone cluster 1, H2bk | -1.9 | 3.70E-02 | 1.5 | 8.40E-03 |
| Pla2g1b | phospholipase A2, group IB, pancreas | -1.8 | 6.10E-02 | 1.5 | 6.80E-03 |
| Shank2 | SH3/ankyrin domain gene 2 | -1.8 | 4.50E-02 | 1.5 | 7.50E-02 |
| Cldn3 | claudin 3 | -1.8 | 1.30E-02 | 1.4 | 4.50E-03 |
| Prrg3 | proline rich Gla (G-carboxyglutamic acid) 3 (transmembrane) | -1.8 | 4.20E-02 | 1.5 | 2.90E-02 |
| Hmgn5 | high-mobility group nucleosome binding domain 5 | -1.8 | 3.10E-02 | 1.3 | 4.80E-02 |
| Plagl1 | pleiomorphic adenoma gene-like 1 | -1.8 | 7.10E-02 | 1.7 | 2.00E-02 |
| Atp13a4 | ATPase type 13A4 | -1.7 | 8.20E-02 | 2 | 3.50E-02 |
| Mir341 | microRNA 341 | -1.7 | 8.90E-02 | 1.7 | 6.30E-02 |
| Exph5 | exophilin 5 | -1.7 | 8.70E-02 | 1.3 | 9.80E-02 |
| Sema3c | sema domain, immunoglobulin domain (Ig), short basic domain, secreted, (semaphorin) 3C | -1.7 | 3.60E-02 | 1.7 | 3.60E-02 |
| Uprt | uracil phosphoribosyltransferase (FUR1) homolog (S. cerevisiae) | -1.7 | 6.20E-03 | 1.4 | 4.20E-02 |
| Fancb | Fanconi anemia, complementation group B | -1.7 | 9.90E-03 | 1.4 | 1.30E-02 |
| Prrg4 | proline rich Gla (G-carboxyglutamic acid) 4 (transmembrane) | -1.7 | 3.20E-02 | 1.4 | 6.50E-02 |
| Pcbd1 | pterin 4 alpha carbinolamine dehydratase/dimerization cofactor of hepatocyte nuclear factor 1 alpha (TCF1) 1 | -1.7 | 5.10E-02 | 1.3 | 4.50E-02 |
| Muc20 | mucin 20 | -1.7 | 9.00E-02 | 1.7 | 2.20E-02 |
| Bex1 | brain expressed gene 1 | -1.7 | 2.40E-02 | 1.4 | 8.10E-02 |
| Rbms3 | RNA binding motif, single stranded interacting protein | -1.6 | 2.50E-02 | 1.3 | 7.90E-02 |
| Acss3 | acyl-CoA synthetase short-chain family member 3 | -1.6 | 8.20E-03 | 1.6 | 2.60E-02 |
| Zxda | zinc finger, X-linked, duplicated A | -1.6 | 6.20E-02 | 1.5 | 3.40E-02 |
| Mir432 | microRNA 432 | -1.6 | 6.40E-02 | 1.3 | 8.90E-02 |
| Cpne4 | copine IV | -1.6 | 4.20E-02 | 2.2 | 2.00E-02 |
| Gca | grancalcin | -1.6 | 2.30E-02 | 1.2 | 6.50E-02 |
| Kif11 | kinesin family member 11 | -1.6 | 7.10E-02 | 1.4 | 1.40E-02 |
| Mad2l1 | MAD2 mitotic arrest deficient-like 1 | -1.6 | 4.90E-02 | 1.3 | 8.80E-02 |
| Gstt2 | glutathione S-transferase, theta 2 | -1.6 | 9.80E-02 | 1.3 | 3.20E-02 |
| Mir134 | microRNA 134 | -1.6 | 8.90E-02 | 1.6 | 3.70E-02 |
| Zwilch | zwilch kinetochore protein | -1.5 | 2.40E-02 | 1.3 | 7.30E-02 |
| R3hdml | R3H domain containing-like | -1.5 | 8.10E-02 | 1.5 | 6.20E-02 |
| Fbxo21 | F-box protein 21 | -1.5 | 2.00E-02 | 1.3 | 3.90E-02 |
| Dpagt1 | dolichyl-phosphate (UDP-N-acetylglucosamine) acetylglucosaminephosphotransferase 1 (GlcNAc-1-P transferase) | -1.5 | 4.30E-02 | 1.2 | 8.10E-02 |
| Sult2b1 | sulfotransferase family, cytosolic, 2B, member 1 | -1.5 | 5.00E-02 | 1.3 | 7.30E-02 |
| Anln | anillin, actin binding protein | -1.5 | 7.90E-02 | 1.4 | 6.20E-02 |
| Fut2 | fucosyltransferase 2 | -1.5 | 6.10E-02 | 1.4 | 3.20E-02 |
| Ica1 | islet cell autoantigen 1 | -1.5 | 6.60E-02 | 1.3 | 7.50E-02 |
| Dock9 | dedicator of cytokinesis 9 | -1.5 | 4.80E-02 | 1.2 | 4.10E-02 |
| Nsun7 | NOL1/NOP2/Sun domain family, member 7 | -1.5 | 3.80E-02 | 1.5 | 2.70E-02 |
| Sidt1 | SID1 transmembrane family, member 1 | -1.5 | 8.90E-02 | 1.4 | 3.20E-02 |
| Rpgr | retinitis pigmentosa GTPase regulator | -1.5 | 5.30E-02 | 1.2 | 6.50E-02 |
| Zfp781 | zinc finger protein 781 | -1.5 | 5.80E-02 | 1.4 | 9.80E-02 |
| Cutal | cutA divalent cation tolerance homolog-like | -1.4 | 9.30E-02 | 1.2 | 5.70E-02 |
| Cmtm8 | CKLF-like MARVEL transmembrane domain containing 8 | -1.4 | 9.10E-02 | 1.2 | 8.80E-02 |
| Echdc3 | enoyl Coenzyme A hydratase domain containing 3 | -1.4 | 5.50E-02 | 1.3 | 2.50E-02 |
| Nbea | neurobeachin | -1.4 | 3.00E-02 | 1.2 | 6.10E-02 |
| Slc7a4 | solute carrier family 7 (cationic amino acid transporter, y+ system), member 4 | -1.4 | 7.50E-02 | 1.4 | 9.90E-03 |
| Pgap1 | post-GPI attachment to proteins 1 | -1.4 | 5.10E-02 | 1.3 | 9.20E-02 |
| Fgfr4 | fibroblast growth factor receptor 4 | -1.4 | 6.80E-02 | 1.5 | 6.80E-02 |
| Cdca2 | cell division cycle associated 2 | -1.4 | 6.60E-02 | 1.2 | 4.40E-02 |
| Tmlhe | trimethyllysine hydroxylase, epsilon | -1.4 | 4.10E-02 | 1.5 | 1.00E-01 |
| Slc17a5 | solute carrier family 17 (anion/sugar transporter), member 5 | -1.4 | 9.80E-02 | 1.4 | 1.30E-02 |
| Trim2 | tripartite motif-containing 2 | -1.4 | 7.80E-02 | 1.3 | 4.00E-02 |
| Sytl5 | synaptotagmin-like 5 | -1.4 | 9.20E-02 | 1.7 | 5.40E-02 |
| Nsdhl | NAD(P) dependent steroid dehydrogenase-like | -1.4 | 9.50E-02 | 1.2 | 8.30E-02 |
| Srl | sarcalumenin | -1.4 | 9.90E-02 | 1.2 | 9.20E-02 |
| Lrig1 | leucine-rich repeats and immunoglobulin-like domains 1 | -1.4 | 5.50E-02 | 1.4 | 5.30E-02 |
| Ptprg | protein tyrosine phosphatase, receptor type, G | -1.4 | 8.30E-02 | 1.5 | 3.20E-02 |
| Fcrls | Fc receptor-like S, scavenger receptor | -1.4 | 5.40E-02 | 1.2 | 8.70E-02 |
| Zfp280c | zinc finger protein 280C | -1.4 | 4.20E-02 | 1.7 | 6.40E-03 |
| Tmem184a | transmembrane protein 184a | -1.4 | 8.60E-02 | 1.3 | 4.40E-02 |
| Nme6 | NME/NM23 nucleoside diphosphate kinase 6 | -1.4 | 9.70E-02 | 1.3 | 8.60E-03 |
| Cenpi | centromere protein I | -1.3 | 2.30E-02 | 1.2 | 8.60E-02 |
| Svip | small VCP/p97-interacting protein | -1.3 | 5.60E-02 | 1.2 | 3.90E-02 |
| Apoo | apolipoprotein O | -1.3 | 8.30E-02 | 1.5 | 1.00E-02 |
| Rpl10 | ribosomal protein L10 | -1.3 | 4.80E-02 | 1.2 | 9.40E-02 |
| Rpl29-ps2 | ribosomal protein L29, pseudogene 2 | -1.3 | 7.10E-02 | 1.3 | 2.00E-02 |
| Serpine2 | serine (or cysteine) peptidase inhibitor, clade E, member 2 | -1.3 | 5.40E-02 | 1.3 | 1.70E-02 |
| Gabrq | gamma-aminobutyric acid (GABA) A receptor, subunit theta | -1.3 | 6.40E-02 | 1.4 | 6.30E-02 |
| Tmtc2 | transmembrane and tetratricopeptide repeat containing 2 | -1.3 | 4.30E-02 | 1.4 | 2.80E-03 |
| Fgf1 | fibroblast growth factor 1 | -1.3 | 4.10E-02 | 1.3 | 3.70E-02 |
| Mcts1 | malignant T cell amplified sequence 1 | -1.3 | 4.20E-02 | 1.2 | 4.90E-02 |
| Tmem29 | transmembrane protein 29 | -1.3 | 2.10E-02 | 1.4 | 4.00E-03 |
| Dalrd3 | DALR anticodon binding domain containing 3 | -1.3 | 3.10E-02 | 1.4 | 2.90E-02 |
| Vbp1 | von Hippel-Lindau binding protein 1 | -1.3 | 5.40E-02 | 1.2 | 7.50E-02 |
| Aspm | asp (abnormal spindle)-like, microcephaly associated (Drosophila) | -1.3 | 6.90E-02 | 1.2 | 4.80E-02 |
| Aurkb | aurora kinase B | -1.3 | 7.90E-02 | 1.3 | 3.90E-02 |
| Mtcp1 | mature T cell proliferation 1 | -1.3 | 6.70E-02 | 1.4 | 1.10E-02 |
| AU019823 | expressed sequence AU019823 | -1.3 | 7.50E-02 | 1.3 | 2.70E-02 |
| Alg13 | asparagine-linked glycosylation 13 | -1.3 | 5.10E-02 | 1.2 | 7.90E-02 |
| BC023829 | cDNA sequence BC023829 | -1.3 | 4.10E-02 | 1.2 | 8.20E-02 |
| Brcc3 | BRCA1/BRCA2-containing complex, subunit 3 | -1.3 | 6.80E-02 | 1.2 | 7.80E-02 |
| Cep70 | centrosomal protein 70 | -1.3 | 8.60E-02 | 1.3 | 4.10E-02 |
| Poc1b | POC1 centriolar protein homolog B (Chlamydomonas) | -1.3 | 8.80E-02 | 1.2 | 9.40E-02 |
| Proser3 | proline and serine rich 3 | -1.3 | 8.60E-02 | 1.3 | 8.10E-02 |
| Zfp229 | zinc finger protein 229 | -1.3 | 4.60E-02 | 1.3 | 4.90E-02 |
| Parpbp | PARP1 binding protein | -1.3 | 2.80E-02 | 1.2 | 9.00E-02 |
| Snora52 | small nucleolar RNA, H/ACA box 52 | -1.3 | 9.00E-02 | 1.3 | 4.10E-02 |
| Bivm | basic, immunoglobulin-like variable motif containing | -1.3 | 8.00E-02 | 1.3 | 3.30E-03 |
| Plp2 | proteolipid protein 2 | -1.2 | 8.40E-02 | 1.2 | 4.30E-02 |
| Zfp202 | zinc finger protein 202 | -1.2 | 8.80E-02 | 1.2 | 7.00E-02 |

*fc:* fold change, *OE:* overexpression, *KD:* knockdown
